# Systematic Fluorination Is a Powerful Design Strategy toward Fluid Molecular Ferroelectrics

**DOI:** 10.1021/jacs.4c16555

**Published:** 2025-01-24

**Authors:** Calum J. Gibb, Jordan Hobbs, Richard J. Mandle

**Affiliations:** † School of Chemistry, University of Leeds, Leeds LS2 9JT, U.K.; ‡ School of Physics and Astronomy, University of Leeds, Leeds LS2 9JT, U.K.

## Abstract

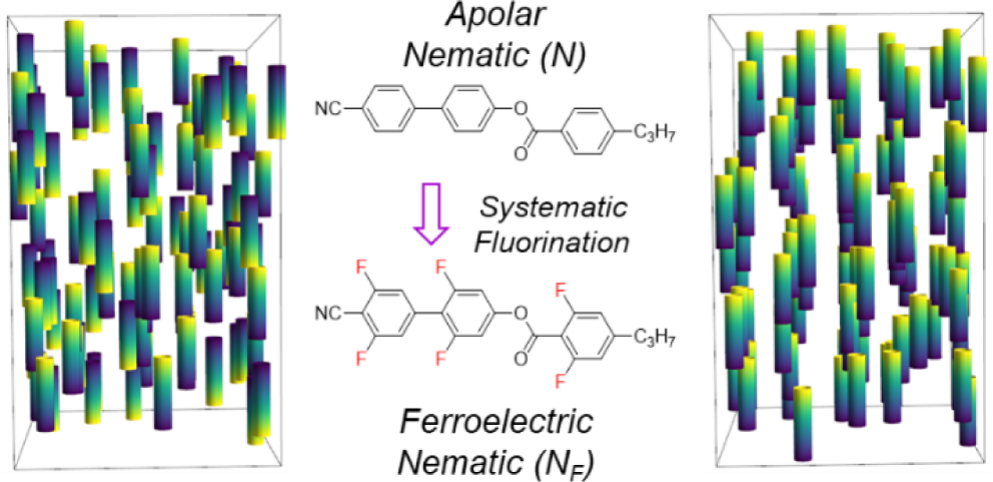

Ferroelectric nematic (N_F_) liquid crystals combine liquid-like fluidity and orientational order of conventional nematics with macroscopic electric polarization comparable in magnitude to solid-state ferroelectric materials. Here, we present a systematic study of twenty-seven homologous materials with various fluorination patterns, giving new insight into the molecular origins of spontaneous polar ordering in fluid ferroelectric nematics. Beyond our initial expectations, we find the highest stability of the N_F_ phase to be in materials with specific fluorination patterns rather than the maximal fluorination, which might be expected based on simple models. We find a delicate balance between polar and apolar nematics, which is entirely dictated by the substitution of the fluorine atoms. Aided by electronic structure calculations, we show this to have its origins in the radial distribution of charge across the molecular surface, with molecules possessing a more oscillatory distribution of electrons across their surfaces and possessing a higher propensity to form polar nematic phases. This work provides a new set of ground rules and design principles that can inform the synthesis of future ferroelectric nematogens.

## Introduction

**Figure 1:**
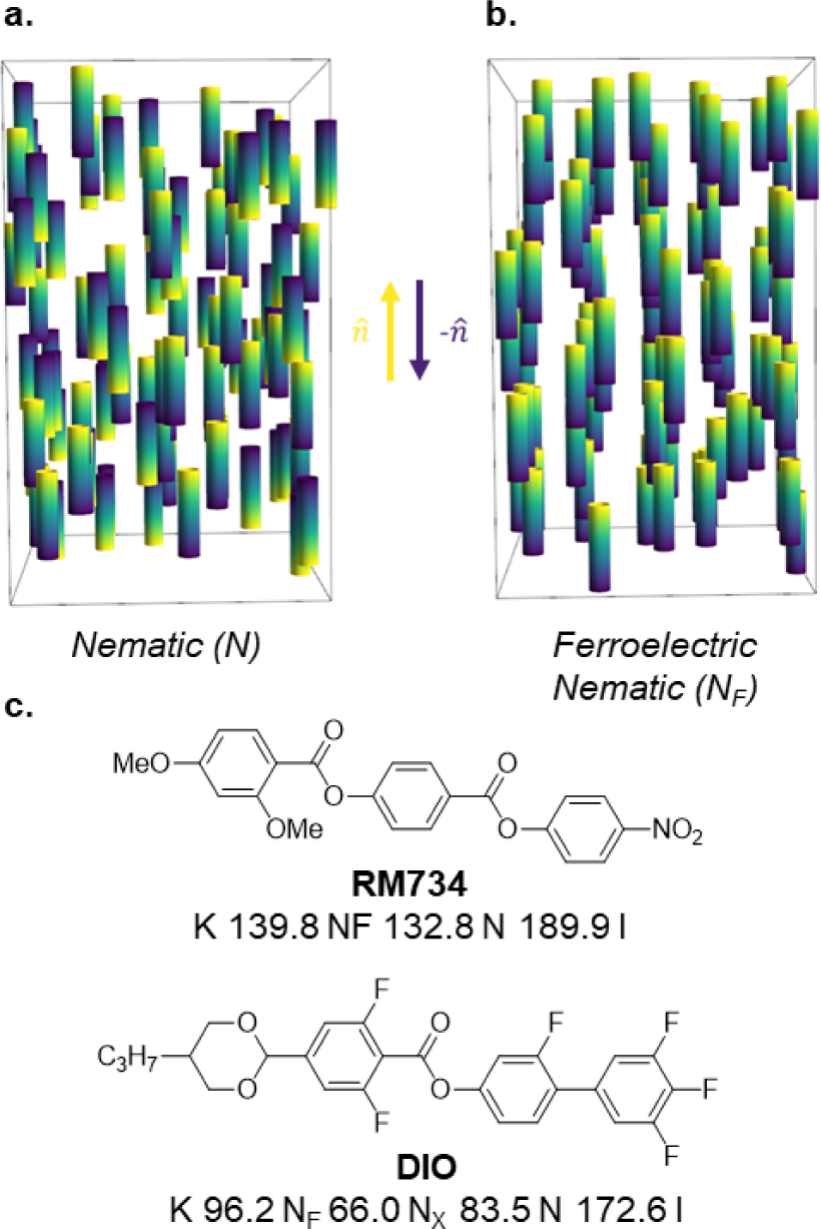
Schematic representations of (a) as the apolar nematic (N) phase and (b) as the ferroelectric nematic (N_F_) phase. Both phases only have orientational ordering with molecules aligning along a unit vector termed the director (*n̂*). In the polar, N_F_ case, the molecular electric dipole moments of the molecules spontaneously align, resulting in a phase possessing a macroscopic polarization (i.e., −*n̂* ≠ *n̂*)—this is not observed in the conventional, apolar N phase where molecules can freely rotate about the short molecular axis (i.e., *n̂* = *n̂*); and (c) the chemical structures of the archetypal ferroelectric nematic materials, **RM734**(1) and **DIO**,^2^ with their associated transition temperatures (°C).

Through its applications in display devices, the conventional nematic (N) phase (Figure 1a) underpinned a revolution in display technology since the mid-1980s. The ferroelectric nematic (N_F_) phase was recently discovered in 2017^1,2^ and combines the orientational order of conventional nematic liquid crystals with polar ordering, resulting in a 3D fluid with bulk electric polarization whose magnitude is comparable to solid-state ferroelectric materials (Figure 1b).^3,4^ The discovery of the N_F_ phase at equilibrium has garnered significant scientific interest due to its potential to “remake science and technology”.^5−10^ The N_F_ phase combines fluidity with a large spontaneous polarization value, resulting in nonlinear optical properties^11,12^ and significant electric field screening potential.^13^ Together, these point to a plethora of possible end-uses including electrooptic devices,^14−16^ production of entangled photon pairs,^17^ tunable lasers,^18^ and reflectors^19^ to name but a few possible applications.

While the rich physics of the N_F_ phase is rightly celebrated, the molecular basis of this new state of matter is often overlooked. Archetypal materials, such as **RM734**(1) and **DIO**(2) (Figure 1c), have typically been the subjects of most physical investigations but are nonideal for practical applications due to their propensity to suffer from irreversible structural changes at moderate temperatures.^20−22^ The scope of studies into the structure–property relationship within the context of the N_F_ phase to date has been narrow, largely focusing on changes to molecular length, terminal chain length, and small changes in fluorination of the two archetypal materials.^4,23,24^ We considered that by presenting an exhaustive study into fluorination patterns in a simple biphenyl benzoate liquid crystal, we could generate a new structure space that shows the N_F_ phase while also probing the delicate balance between polar and apolar ordering.

**Scheme 1:**
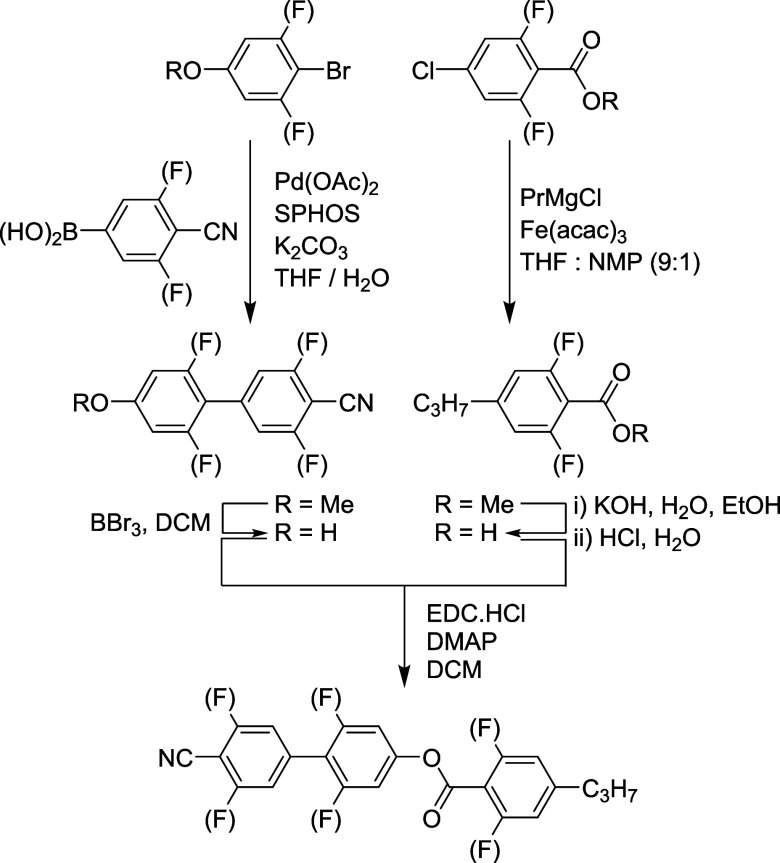
Synthetic Route Used in the Synthesis of Materials **1**–**27**

The chemical structure–property relationships governing the molecular origins of the N_F_ phase are still relatively unknown. To date, most molecules which exhibit the N_F_ phase all possess significant molecular electric dipole moments (μ) (circa 8 D),^12,25^ although there is still debate about the role dipole moments play in the formation of the N_F_ phase.^4,12,25−28^ To this end, we elected to design a new chemical structure space such that the positions of all fluorine atoms are additive to the overall longitudinal molecular electric dipole moment, systematically increasing the number and position of the substituents in order to screen all possible fluorination patterns of our chosen structure type (Scheme 1). This culminated in the systematic synthesis of twenty-seven homologues, which possess moderate to large values of μ (**1**–**27**). Full synthetic details, including spectroscopic and purity data, can be found in the Supporting Information. For simplicity, we refer to compounds **1**–**27** by the acronym *X*.*Y*.*Z* where *X*, *Y*, and *Z* refer to the number of fluorine substituents on each aromatic ring, beginning with the nitrile-bearing ring and ending with the benzoate (for example, the most fluorinated material synthesized (**1**) is given the acronym **2.2.2**).

## Results and Discussion

**Figure 2:**
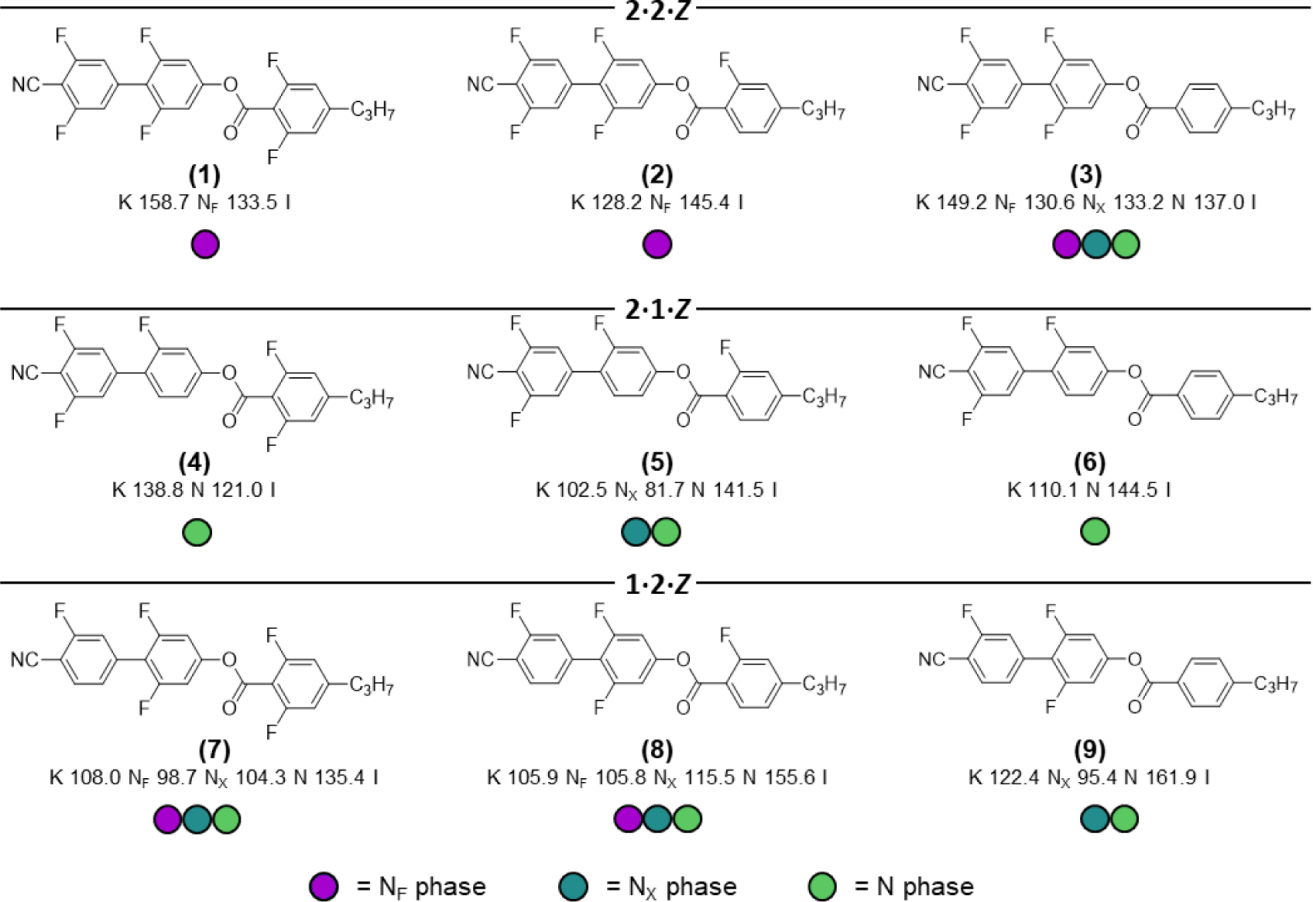
Chemical structures, phase sequences, and associated transition temperatures (°C) of materials **1**–**9**. The analogous data for **10**–**27** may be found in the Supporting Information. *K* = melting point; N_F_ = ferroelectric nematic; N_X_ = antiferroelectric nematic; N = nematic; I = isotropic liquid.

**Figure 3:**
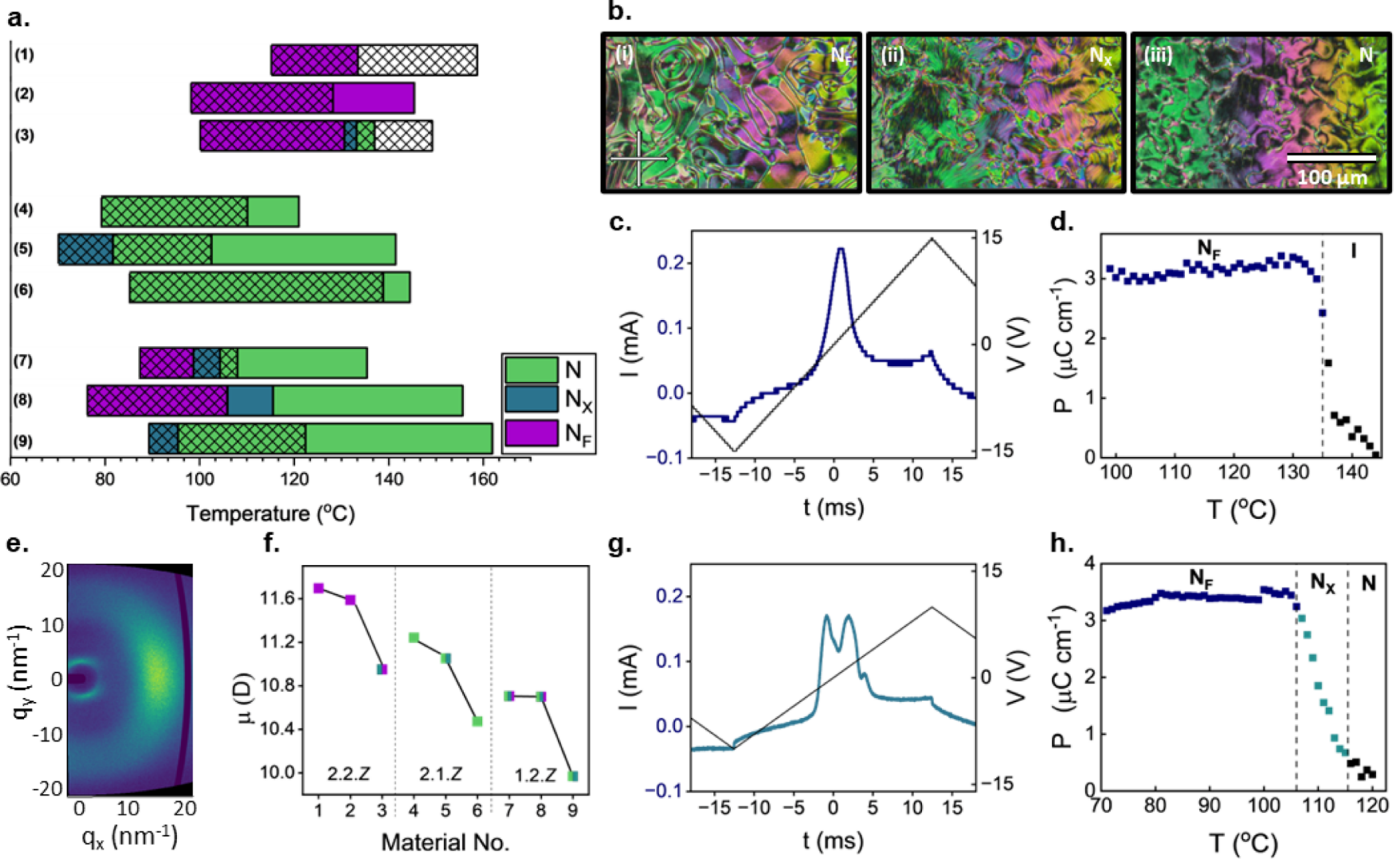
(a) The dependence of the transition temperatures on systematic fluorination for materials **1**–**9**. The hash bar indicates the meting point of the material; any transition within the hashed region is supercooled below the melting point; (b) POM micrographs depicting the (i) N_F_, (ii) N_X_, and (iii) N phases observed for **2.2.0** (**3**) at 137, 134, and 131 °C, respectively. Images were taken of a thin sample sandwiched between untreated glass slides; (c) current response trace measured for **2.2.2** (**1**) measured at 20 Hz in the N_F_ phase at 105 °C; (d) temperature dependence of spontaneous polarization (*P*_S_) measured for **2.2.2** (**1**); (e) 2D X-ray scattering pattern obtained for **1.2.1** (**8**) at 98 °C in the N_F_ phase showing the nematic-like ordering of molecules; (f) the dependence on the magnitude of the longitudinal molecular dipole moment (μ) on systematic fluorination. The color of each data point indicates the phase sequence exhibited by the homologue; (g) current response trace measured for **1.2.1** (**8**) measured at 20 Hz in the N_X_ phase at 110 °C; and (h) temperature dependence of spontaneous polarization (*P*_S_) measured for **1.2.1** (**8**).

Materials **10**–**27** contain the least F atoms of the materials studied (*F* < 4) and exhibit solely conventional nematic behavior with the exception of **26**, which also exhibits an SmA phase (Table S1). A simple inspection of the nematic to isotropic (I) phase transition temperature (*T*_N–I_) reveals the expected trend whereby increasing the number of F atoms generally leads to a decrease in the values of *T*_N–I_. This simply reflects the changes in free volume afforded by additional F atoms, inhibiting the efficient packing of the molecules into the N phase. Gratifyingly, increasing the number of fluorine substituents yields materials with more interesting mesomorphic behavior (**1**–**9**, Figures 2 and 3a). **2.2.2** (**1**) displays a monotropic N_F_ phase at 133.5 °C, which forms directly from the isotropic liquid. The N_F_ phase was identified first by polarized optical microscopy (POM) by the appearance of a characteristic banded texture (for example, see Figure 3b(i)) followed by the conformation of the transition temperature by differential scanning calorimetry (DSC) (Figure S1). The polar nature of the N_F_ phase was confirmed by a single peak in the current response (Figure 3c). Specifically, for the direct I–N_F_ phase transition, in the isotropic phase, a pretransition field-induced I–N_F_ phase transition is seen from the double peaks in the current trace due to the critical-like first-order nature of the I–N_F_ transition (Figure S2).^29^**2.2.2** also shows an immediate saturation of the spontaneous polarization, indicating a strongly first-order transition from complete isotropy to an N_F_ phase (Figure 3d). X-ray scattering measurements confirmed the assignment of the N_F_ phase, where diffuse signals are seen in both the wide and small angle regions, indicating orientational ordering of the molecules with no positional order, respectively, across the entire phase range (for example, see Figure 3e).

Surprisingly, removal of a single fluorine atom (to afford **2.2.1** (**2**)) leads to a significant increase in the N_F_–I transition temperature (*T*_NF–I_) resulting in the N_F_ phase observed for **2.2.1** being enantiotropic, despite this modification leading to a decrease in molecular electric dipole moment (μ) (Figure 3f). Interestingly, this appears to be a general trend when comparing **1**–**9** whereby, regardless of the fluorination pattern and the phase sequence of the material, homologues with ***X***.***Y***.**1** fluorination patterns have significantly higher transition temperatures associated with polar order (i.e., N_F_–N_X_ or N_X_–N) than their more fluorinated counterparts while possessing smaller values of μ. Considering the current understanding of the molecular origins of the N_F_ phase, one might assume that maximal fluorination would result in the most desirable materials. This, therefore, makes this result rather unexpected and something that we will revisit shortly.

Further removal of a fluorine substituent from the ***Z***-ring affords **2.2.0** (**3**). For **2.2.0**, the N_F_ phase is preceded by a paraelectric N and subsequent antiferroelectric nematic (N_X_), sometimes referred to as the N_S_^30^ or SmZ_A_^31^ phase. The N_X_ phase was identified by the appearance of a distorted banded texture by POM (Figure 1b(ii)) and a double peak in the current response on either side of voltage polarity reversal under an applied electrical field (Figure 1g). The spontaneous polarization also saturates almost immediately at the N_X_–N_F_ phase transition rather than showing a continuous increase toward the saturation value of *P*_S_, which is more generally observed for ferroelectric nematogens (for example, see Figure 3h).^2,32−35^ Despite the increase in *T*_NF–I_ observed when removing a single fluorine substituent, removal of a further fluorine (**2.2.0**) results in a significant decrease in the transition temperatures associated with polar order, in this case the N_X_–N transition, compared with the most fluorinated homologue. This modification also decreases the value of μ.

Decreasing the number of F substituents on either the ***X***- or ***Y***-rings yields two pairs of isomeric structures, **2.1.*****Z*** (**4**–**6**) and **1.2.*****Z*** (**7**–**9**). Although the **2.1.*****Z*** materials possess notably higher molecular electrical dipole moments (Figure 3f), the three **1.2.*****Z*** homologues exhibit a greater number of polar LC phases, with their associated transitions to polar order occurring at higher temperatures (i.e., higher values of *T*_NX–N_). This is perhaps a surprising observation that reinforces the emerging observation that, beyond molecules possessing a sufficient molecular electrical dipole moment such that a polar nematic phase may form, practically, the magnitude of μ does not appear to impact the thermal stability of polar nematic phases. Following on from this, when considering the fluorination pattern of the ***Z***-ring in materials **4**–**9**, the values of *T*_N–I_ behave similarly to **10**–**27** discussed above, whereby increasing the number of F atoms leads to a decrease in the nematic to isotropic transition temperatures. Despite this expected behavior in *T*_N–I_, we still observed that homologues with ***Z*** = 1 have more stable polar phases, evidenced by their higher N_X_–N transition temperatures (*T*_NX–N_). When taken together, these two rather surprising observations indicate that the molecular origins of polar nematic phase behavior are clearly different from those describing the formation of the conventional nematic phase. A complete model describing the formation of polar nematic phases would clearly be highly complex, more so than one describing the formation of conventional nematic materials, and such a model would clearly have to go beyond the basic idea of molecules possessing large molecular dipole moments.

Madhusudana proposed a model in which polar order is suggested to arise from side-to-side electrostatic interactions between molecules.^36^ For the conventional, apolar nematic phase, molecules tend to preferentially adopt antiparallel conformations relative to their closest neighbors, as this helps minimize the dipolar energy of the system.^37,38^ Madhusudana suggests that it is possible for molecules to adopt parallel orientations if the electrostatic potential (ESP) along the long molecular axis oscillates between areas of positive and negative potential, as this results in attractive interactions between parallel neighbors.^36^ More specifically, the suggestion was that low charge densities at the tops and tails of the molecule would be beneficial for the promotion of parallel-promoting interactions. The model has been applied to a variety of known ferroelectric nematogens,^39−42^ to explain changes in polar LC phase behavior. While a model based solely on surface charge interactions alone likely cannot completely account for the formation of the N_F_ phase, an opinion also supported by considering how these electrostatic interactions actually contribute to the free energy of these systems,^26,27^ electrostatic interactions are likely a significant factor in stabilizing longitudinally polar LC phases and are intrinsically linked to the molecular structure of these polar LCs.

**Figure 4:**
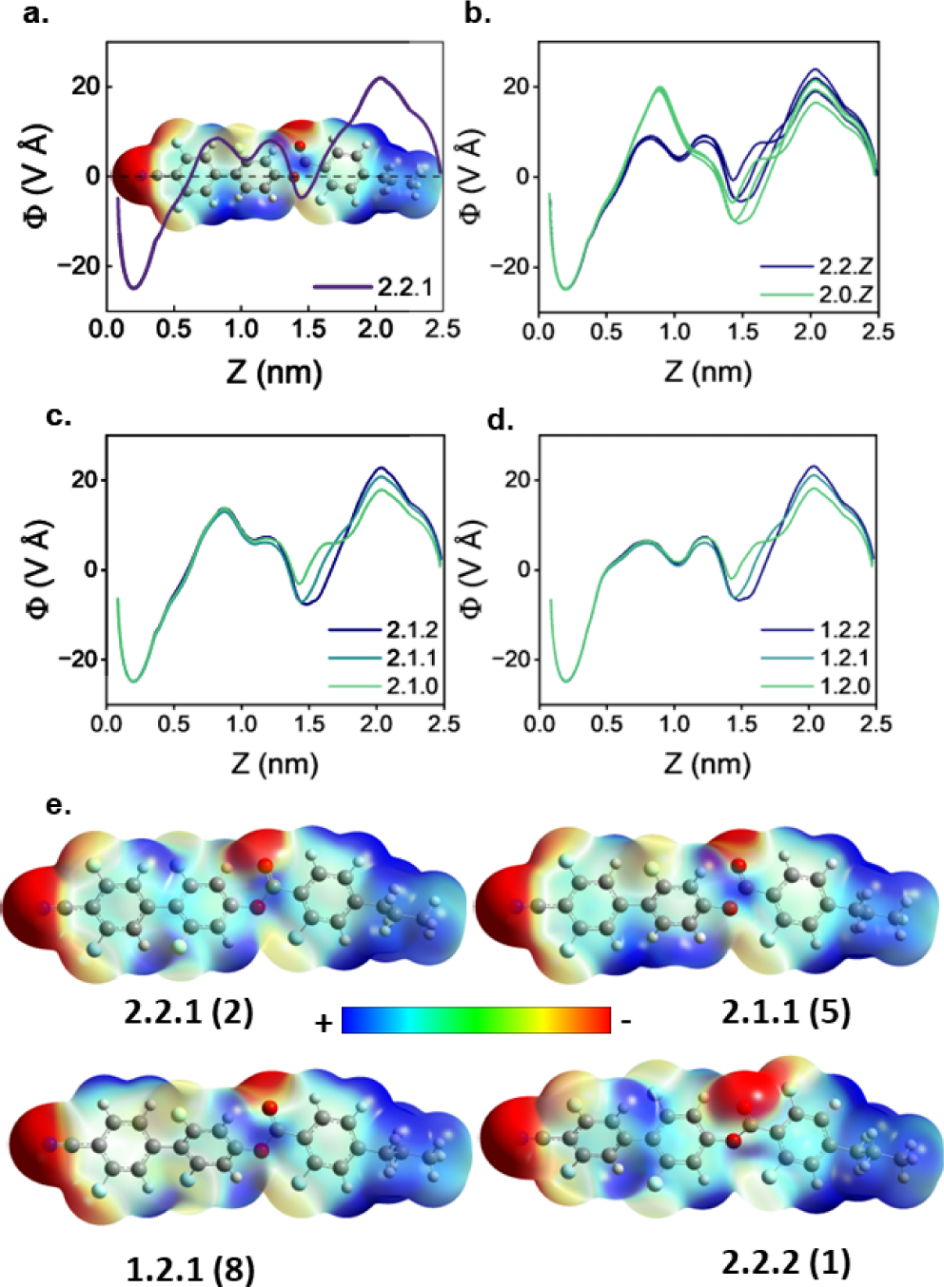
(a) 3D ESP surface and the resulting 1D longitudinal charge density wave calculated for 2.1.1; (b) 1D longitudinal charge density waves calculated for the three 2.2.*Z* and 2.0.*Z* homologues showing the more uniform oscillatory structure of the charge density wave for the 2.2.*Z* homologues, which results in the formation of the polar nematic phases. The greater amplitude of the charge density wave for 2.0.*Z* promotes antiparallel associations between the molecules resulting in the solely conventional nematic behavior observed experimentally; a comparison of the 1D longitudinal charge density waves of the (c) **2.1.*Z*** (**4**–**6**) and (d) **1.2.*Z*** (**7**–**9**) (ii) homologues, indicating the more oscillatory structure of the latter structures, which exhibit more polar nematic phases despite having lower values of μ; and (e) 3D ESP surfaces for **2.2.1** (**2**) (top left), **2.1.1** (**5**) (top right), **1.2.1** (**8**) (bottom left), and **2.2.2** (**1**) (bottom right). For *Z* = 1, the 3D ESP surface is more spatially uniform due to the position of the appended F atom complimenting the position of the oxygen atom of the ester carbonyl.

The systematic approach to selective fluorination undertaken in this work provides us with the unique opportunity to apply the model proposed by Madhusudana to an entire series of homologues, where we have a number of homologues exhibiting both polar and apolar nematic phases. To do this, we compute the 3D molecular ESP isosurface (at the DFT:B3LYP-GD3BJ/cc-pVTZ level)^43−^ and radially average the ESP at an electron density isovalue of 0.0004 as a function of the long molecular axis for all **1**–**27**, allowing the longitudinal ESP surface to be visualized in 1D space (Figure 4a). The resultant 1D ESP plots provide insight into potential, favorable lateral interactions which stabilize the polar nematic phase behavior observed for a select number of **1**–**27**. We provide further complete 3D ESP surfaces and the resulting 1D reduced data in the Supporting Information (Figures S3–S5) as well as further details of this method.

Inspection of these plots for homologues exhibiting polar phase behavior (for example, **2.2.***Z*, Figure 4b, purple) and those that exhibit solely conventional nematic behavior (**2.0.***Z*, Figure 4c, green) reveal stark differences in the longitudinal surface charge density across the biphenyl structure. For the three **2.2.*****Z*** homologues, the charge density oscillates almost sinusoidally across the biphenyl structure with only small changes in the amplitude of the oscillations. In contrast, the variation in charge density across the **2.0.*****Z*** homologues is more pronounced, lacking a clear oscillatory structure. We stress that although the radially averaged ESP of the biphenyl region is overall always positive, regardless of the fluorination pattern, appended fluorine atoms tend to induce regions of more negative ESP—leading to more favorable, lateral interactions between parallel molecules. The uniformity of the oscillations for 2.2.Z, 1.2.Z (and to a lesser extent 2.1.*Z*) is indicative of the spatial uniformity of these positive and negative regions of the 3D ESP surface, where the regions of positive and negative potential all correspond to regions of similar size, something not observed for homologues **10**–**27**, which contain fewer F atoms (Figures S4, S6).

Probing more deeply, when considering the spatial uniformity of the oppositely charged regions on the ESP surface, the greater electronegativity of the nitrile moiety present on the *X*-ring appears to negate the effect of removing a fluorine atom (Figure 4c(i)) whereas removal of an F atom from the *Y*-ring results in a less uniform oscillatory structure of surface charge (Figure 4c(ii)) and thus correspondingly less stable polar mesophases for the **2.1.*****Z*** (**4**–**6**) molecules vs the **1.2.*****Z*** (**7**–**9**) set despite the former having larger longitudinal molecular dipole moments. Reducing fluorination of the *Z*-ring has a much smaller effect on the structure of the 1D ESP, and so fluorination of the *Z*-ring has a much smaller impact on the thermal stability of polar nematic phases, though we do note that homologues with the ***X***.***Y***.**1** fluorination patterns consistently have slightly higher polar–apolar transition temperatures (i.e., *T*_NF–I_ and *T*_NX–N_). Inspection of the 3D ESP isosurface shows that it is actually the ***X***.***Y***.**1** homologues (for example, Figure 4e [top]) that have the most spatially uniform ESP as the appended fluorine atom matches to the carbonyl atom of the ester group. Adding or removing fluorine (for example, Figure 4e [bottom]) distorts the uniformity slightly, leading to the destabilization of polar mesophases for those homologues and hence decreases the stability of the polar mesophases.

**Figure 5:**
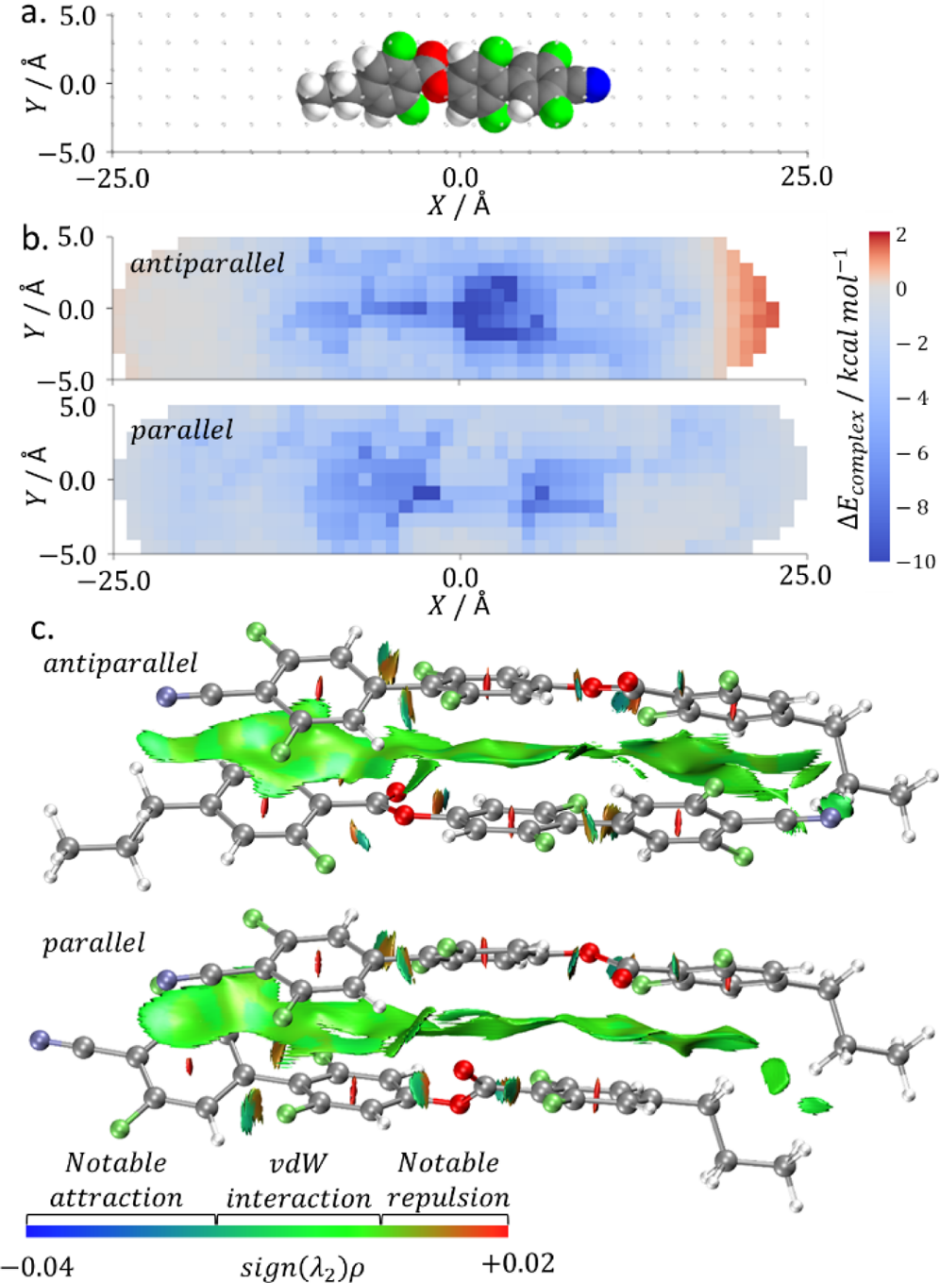
(a) Molecular structure of **2.2.2** (**27**) with the grid of translation vectors used in the bimolecular PES shown as points, (b) rigid bimolecular potential energy surfaces for two molecules of **2.2.2** (**27**) in (anti)parallel orientation; a diverging colorscale with midpoint at zero is used to highlight attractive (blue) and repulsive (red) regions, (c) interaction region indicator (IRI) isosurface (isovalue 1.0) for the global energy minimum of the antiparallel and parallel forms of **2.2.2** (**27**), identified via the bimolecular PES; the colorbar indicates the different types of noncovalent interactions present on the isosurface.

Examination of the bimolecular potential energy surface with electronic structure calculations is a logical extension of this simple model. This comprises a rigid bimolecular potential energy scan (PES), beginning from a DFT-optimized geometry, in which the position of the second molecule is translated over the *x*/*y*/*z* dimensions (Figure 5a). To simplify these calculations, we calculated only the limiting cases of two molecules in a parallel and antiparallel orientation. For each set of translation vectors, we obtain the counterpoise-corrected complexation energy (at the DFT:B3LYP-GD3BJ/cc-pVTZ level in Gaussian G16).^43−45^ The translation vectors and complexation energies are then used to produce a bimolecular PES (Figure 5b for **2.2.2** (**1**)). In the antiparallel configuration, repulsive regions are observed that arise from the close proximity of like charges, which are absent for the parallel configuration. The size and depth of the repulsive region in the antiparallel configuration are dependent on the degree of fluorination at the nitrile terminus of the molecule. Put another way, the preference for polar order arises, at least in part, from the enthalpic cost of antiparallel packing of such polar rod-like molecules.

While these calculations do give a better understanding of the lateral interaction between **1**–**27**, changes in the fluorination pattern between homologues affect the specific preferred pairing modes in ways that are difficult to infer from the rigid scans presented here. To that end, and given that both parallel and antiparallel potential energy surfaces have minima with large negative complexation energies, we elected to refine our calculations by extracting five discrete minima for each compound (in both parallel and antiparallel orientations), which we then perform optimization (at the B3LYP-GD3BJ/cc-pVTZ level). The resultant interaction region indicator (IRI)^47^ isosurface allows for the visualization of the noncovalent interactions between pairs of molecules (Figure 5c for **2.2.2** (**1**)). In the case of the molecules presented here, the dominant interaction is offset π–π stacking of the biphenyl units, with a small contribution arising from the *Z*-ring. Although for none of **1**–**27** does the global minima in complexation energy for the parallel packed molecules become lower than antiparallel, parallel packing results in multiple positions of relatively comparable energy, while antiparallel packing results in only a singular region of highly negative complexation energy as well as regions of repulsive positive complexation energy. Notably, increasing the number of appended fluorine atoms does result in the global minima for each packing mode being considerably closer in energy, particularly for the molecules showing polar phases. This may result in a situation where the increased entropy of multiple possible complexation positions counteracts the slightly increased enthalpic cost of not existing in the global minima, resulting in an overall reduced bulk free energy of parallel arrangement of the molecules.

## Conclusions

In summary, against current thinking, we have shown that the maximal fluorination of these materials does not necessarily result in maximal polar mesophase stability, with specific fluorination patterns being preferred. This also highlights that the magnitude of the longitudinal dipole moment is not the most important metric for predicting polar phase behavior even in chemically similar compounds. We have complemented our synthetic efforts with a series of computational methodologies that provide insight into the molecular origins of polar nematic phase behavior by probing the lateral interactions between molecules necessary for these phases to form. We show that rather than considering the electrostatic interactions as 1D rod-like objects, consideration must be given to the resulting 3D ESP of the molecule. In the context of the Madhusudana model,^36^ we suggest that the broader principles of the model translate physically at a molecular level (i.e., varying regions of charge density promote parallel arrangement of molecules through intermolecular interactions); however, we show that the suggestion of low charge density at the tops and tails of N_F_ mesogens is not physically representative of any of the currently discovered N_F_ materials. We further suggest that the spatial uniformity of the regions of varying charge density is an additional parameter important for the formation of the N_F_ phase and that there may possibly be unconsidered entropic contributions to the emergence of polar order. Moreover, evaluation of the bimolecular potential energy surfaces, coupled with the interaction region indicator, shows the dominant mode of interaction between molecules to be offset π–π stacking rather than the often-quoted dipole–dipole interactions.

## Supporting Information Available

Supplemental liquid crystal characterization and details of the chemical synthesis of materials **1–27**, including structural characterization (PDF)
