# Supplementary material for: Systematic Fluorination Is a Powerful Design Strategy toward Fluid Molecular Ferroelectrics
Source: J Am Chem Soc. 2025 Jan 24;147(5):4571–7. doi: 10.1021/jacs.4c16555 (PMC11803714; doi:10.1021/jacs.4c16555)
Supplement: Supplementary file 1 — ja4c16555_si_001.pdf [file ja4c16555_si_001.pdf]

Systematic Fluorination is a Powerful Design Strategy Towards Fluid Molecular Ferroelectrics

Supplemental Information

Calum J. Gibb*[1], Jordan Hobbs [2], Richard. J. Mandle [1,2]

[1]School of Chemistry, University of Leeds, Leeds, UK, LS2 9JT
[2]School of Physics and Astronomy, University of Leeds, Leeds, UK, LS2 9JT

*c.j.gibb@leeds.ac.uk

Contents

1 **Supplementary Methods**
- 1.1 Chemical synthesis
- 1.2 Chemical characterisation methods
- 1.3 Mesophase characterisation
- 1.4 X-ray scattering
- 1.5 Measurements of spontaneous polarization (P_S)
- 1.6 DFT calculations

2 **Supplemental results**

3 **Organic Synthesis**
- 3.1 Synthesis of 4-(2 fluoro-4-hydroxyphenyl)-2,6-difluorobenzonitrile.
- 3.2 4-(2,6-difluoro-4-hydroxyphenyl)-2-fluorobenzonitrile
- 3.3 Synthesis of materials **1-27**
- 3.4 Example structural characterisation

4 **Supplemental References**

1 Supplementary Methods

1.1 Chemical Synthesis

Chemicals were purchased from commercial suppliers (Fluorochem, Merck, Apollo Scientific) and used as received. Solvents were purchased from Merck and used without further purification. Reactions were performed in standard laboratory glassware at ambient temperature and atmosphere and were monitored by TLC with an appropriate eluent and visualised with 254 nm or 365 nm light. Chromatographic purification was performed using a Combiflash NextGen 300+ System (Teledyne Isco) with a silica gel stationary phase and a hexane/ethyl acetate gradient as the mobile phase, with detection made in the 200-800 nm range. Chromatographed materials subjected to re-crystallisation from an appropriate solvent system.

1.2 Chemical Characterisation Methods

NMR was performed using a Bruker Avance III HDNMR spectrometer operating at 400 MHz, 100.5 MHz or 376.4 MHz (^{1}H, ^{13}C{^{1}H} and ^{19}F, respectively). Unless otherwise stated, spectra were acquired as solutions in deuterated chloroform, coupling constants are quoted in Hz, and chemical shifts are quoted in ppm.

1.3 Mesophase Characterisation

Transition temperatures and measurement of associated latent heats were measured by differential scanning calorimetry (DSC) using a TA instruments Q2000 heat flux calorimeter with a liquid nitrogen cooling system for temperature control. Between 3-8 mg of sample was placed into T-zero aluminium DSC pans and then sealed. Samples were measured under a nitrogen atmosphere with 10 °C min^{-1} heating and cooling rates. The transition temperatures and enthalpy values reported are averages obtained for duplicate runs. In general LC phase transition temperatures are measured on cooling from the onset of the transition while melt temperatures were measured on heating to avoid crystallization loops that can occur on cooling. Phase identification by polarised optical microscopy (POM) was performed using a Leica DM 2700 P polarised optical microscope equipped with a Linkam TMS 92 heating stage. Samples were studied sandwiched between two untreated glass coverslips.

1.4 X-ray Scattering

X-ray scattering measurements, both small angle (SAXS) and wide angle (WAXS) where recorded using an Anton Paar SAXSpoint 5.0 beamline machine. This was equipped with a primux 100 Cu X-ray source with a 2D EIGER2 R detector. The X-rays had a wavelength of 0.154 nm. Samples were filled into thin-walled quartz capillaries 1 mm thick. Temperature was controlled using an Anton Paar heated sampler with a range of -10 ℃ to 107 ℃ and the samples held in a chamber with an atmospheric pressure of <1 mBar. Samples were held at 107 ℃ to allow for temperature equilibration across the sample and then slowly cooled while stopping to record the 2D scattering patterns. The 2D patterns are then radially integrated to obtain 1D patterns.

1.5 Measurement of Spontaneous Polarization (P_S)

Spontaneous polarisation measurements are undertaken using the current reversal technique [1,2]. Triangular waveform AC voltages are applied to the sample cells with an Agilent 33220A signal generator (Keysight Technologies), and the resulting current outflow is passed through a current-to-voltage amplifier and recorded on a RIGOL DHO4204 high-resolution oscilloscope (Telonic Instruments Ltd, UK). Heating and cooling of the samples during these measurements is achieved with an Instec HCS402 hot stage controlled to 10 mK stability by an Instec mK1000 temperature controller. The LC samples are held in 4µm thick cells with no alignment layer, supplied by Instec. The measurements consist of cooling the sample at a rate of 1 Kmin^{-1} and applying a set voltage at a frequency of 10 Hz. The voltage was set such that it would saturate the measured P_S and was determined before final data collection.

There are three contributions to the measured current trace: accumulation of charge in the cell (I_c), ion flow (I_i), and the current flow due to polarisation reversal (I_p). To obtain a P_S value, we extract the latter, which manifests as one or multiple peaks in the current flow, and integrate as:

$$P_S = \int \frac{I_p}{2A} dt \qquad \textbf{(1)}$$

where A is the active electrode area of the sample cell. For the N, N_X and, to a lesser extent, the N_F phase, significant amounts of ion flow is present. For materials that showed a paraelectric N phase followed by the anti-ferroelectric N_X phases, the N phase always showed some pre-transitional polarisation as well as the significant ion flow mentioned previously. The P_S of the N_X phases was obtained by integrating the peak least affected by ion flow and then doubled to get the total area under both peaks [3].

1.6 DFT Calculations

Electronic structure calculations were performed using Gaussian G16 revision C.02 [4] and with a B3LYP-GD3BJ/cc-pVTZ [5–8] basis set. Obtained structures were verified as a minimum from frequency calculations. Electrostatic potential (ESP) surfaces were calculated by using the *formchk* and *cubegen* utilities. Both the electron density and ESP cube files were calculated using “fine” data resolution. The 3D ESP surface is displayed at an electron density iso-surface of 0.0004.

The 3D data was reduced into 1D through the following steps. The electron density and ESP cube files are structured such that the long molecule axis of the molecule is centred along the z-axis of the data in each cube file. Each step in the z-axis is taken as a single plane through the molecule at that point. An iso-contour through the electron density cube file is found at some isovalue (here 0.0004 as used to mimic the 3D surfaces). The values of the ESP data that then fall on this iso-contour route are then found. These values reflect the 3D surface visualised in figure S6 exactly. We assume free rotation around the long molecule axis and so average the entire ESP data that falls along the iso-contour. This gives the average ESP value that a neighbouring molecule will “feel” for timescales longer that those of rotation around the long axis.

A further step of rescaling the values obtained by the length of the contour allows to account for the fact that at the molecular extremes the values are distorted by the reduction in molecular volume. This final step effectively gives the ESP as electric flux i.e. the strength of the electric field due to the molecular dipole through the contour.

2 Supplementary Results

Table S1. Phase sequences of **1-27** and their associated transition temperatures (℃) The fluorination pattern value indicates the number of fluorine atoms of the 4-(per)flurobenzoic acid (indicated on the structure below). **24** degraded before T_{NI} was identified

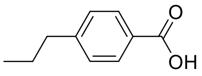

0

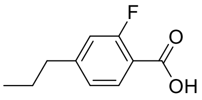

1

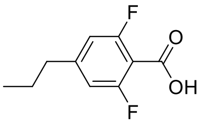

2

Compound	1-nitrile-n-n'-(per)flurobiphenyl-4'-phenol	Fluorination pattern	Phase Sequence and associated transition temperatures / ºC
**1**	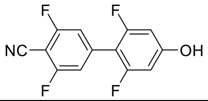	2	K 158.7 N_F 133.5 Iso
**2**		1	K 128.2 N_F 145.4 Iso
**3**		0	K 149.2 N_F 130.6 N_X 133.2 N 137.0 Iso
**4**	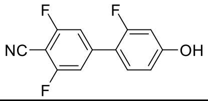	2	K 138.8 N 121.0 Iso
**5**		1	K 102.5 N_X 81.7 N 141.5 Iso
**6**		0	K 110.1 N 144.5 Iso
**7**	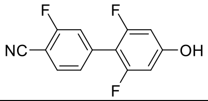	2	K 108.0 N_F 98.7 N_X 104.3 N 135.4 Iso
**8**		1	K 105.9 N_F 105.8 N_X 115.5 N 155.6 Iso
**9**		0	K 122.4 N_X 95.4 N 161.9 Iso
**10**	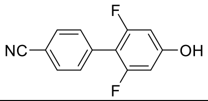	2	K 119.9 N 153.8 Iso
**11**		1	K 111.9 N 177.1 Iso
**12**		0	K 133.4 N 187.7 Iso
**13**	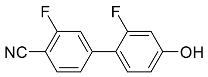	2	K 99.0 N 150.9 Iso
**14**		1	K 121.2 N 181.8 Iso
**15**		0	K 113.7 N 187.9 Iso
**16**	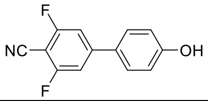	2	K 124.5 N 110.8 Iso
**17**		1	K 104.9 N 151.5 Iso
**18**		0	K 98.6 N 149.3 Iso
**19**	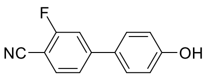	2	K 105.3 N 165.1 Iso
**20**		1	K 84.6 N 200.2 Iso
**21**		0	K 87.6 N 215.3 Iso
**22**	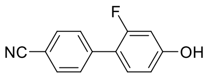	2	K 91.1 N 176.6 Iso
**23**		1	K 108.2 N 204.9 Iso
**24**		0	K 112.4 N >220.0 Iso
**25**	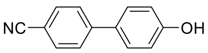	2	K 109.9 N 205.1 Iso
**26**		1	K 100.7 SmA 71.4 N 241.8 Iso
**27**		0	K 100.6 N 255.6 Iso

Table S2. Phase sequences of **1-27** and their associated enthalpy changes (kJ/mol) The fluorination pattern value indicates the number of fluorine atoms of the 4-(per)flurobenzoic acid (indicated on the structure below).

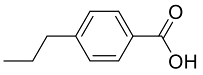

0

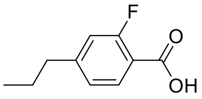

1

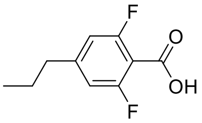

2

Cpd. No.	Biphenyl-type	Fluorination pattern	ΔH_{fus} (kJ/mol)	ΔH_{N-SmA} (kJ/mol)	ΔH_{N-N_F} (kJ/mol)	ΔH_{N-N_X} (kJ/mol)	$\Delta H_{I-N_F/N}$ (kJ/mol)
**1**	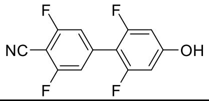	2	38.8	-	-	-	4.7
**2**		1	25.6	-	-	-	4.7
**3**		0	35.5	-	0.6	0.03	1.2
**4**	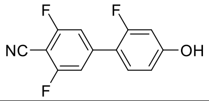	2	35.9	-	-	-	0.6
**5**		1	25.3	-	-	0.02	0.4
**6**		0	27.8	-	-	-	0.4
**7**	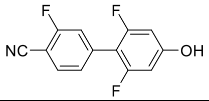	2	36.0	-	0.3	0.01	0.9
**8**		1	38.1	-	0.3	0.01	0.7
**9**		0	30.9	-	-	0.01	1.0
**10**	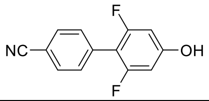	2	29.3	-	-	-	1.2
**11**		1	24.3	-	-	-	1.4
**12**		0	31.6	-	-	-	1.4
**13**	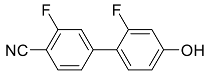	2	37.5	-	-	-	0.6
**14**		1	17.3	-	-	-	0.7
**15**		0	23.3	-	-	-	0.6
**16**	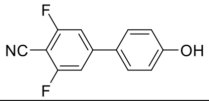	2	30.0	-	-	-	0.2
**17**		1	19.9	-	-	-	0.3
**18**		0	27.2	-	-	-	0.5
**19**	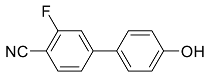	2	33.4	-	-	-	0.5
**20**		1	25.3	-	-	-	0.6
**21**		0	17.7	-	-	-	0.8
**22**	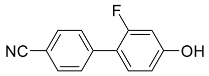	2	23.4	-	-	-	1.1
**23**		1	26.1	-	-	-	1.1
**24**		0	30.1	-	-	-	-
**25**	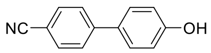	2	30.6	-	-	-	0.7
**26**		1	27.0	0.08	-	-	0.8
**27**		0	25.4	-	-	-	1.1

Table S3. DFT parameters calculated at the DFT:B3LYP-GD3BJ/cc-pVTZ level of theory for compounds **1-27.**

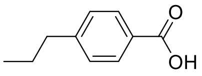

0

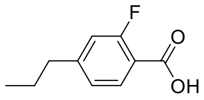

1

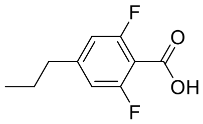

2

Cpd. No.	Biphenyl-type	Fluorination pattern	Dipole Moment / D	Dipole Angle / °	Length / nm	Width / nm	Aspect Ratio
**1**	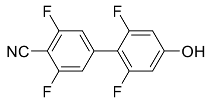	2	11.70	10.2	2.03	0.51	3.99
**2**		1	11.59	3.6	2.02	0.50	4.05
**3**		0	10.95	8.8	2.01	0.50	4.02
**4**	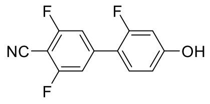	2	11.24	14.0	2.03	0.49	4.11
**5**		1	11.05	7.1	2.02	0.51	3.95
**6**		0	10.47	12.5	2.01	0.51	3.94
**7**	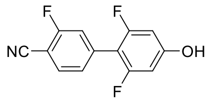	2	10.70	6.3	2.03	0.50	4.06
**8**		1	10.70	1.2	2.02	0.47	4.26
**9**		0	9.97	4.5	2.01	0.48	4.22
**10**	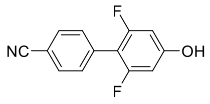	2	9.93	11.9	2.03	0.51	3.97
**11**		1	9.79	4.3	2.02	0.48	4.18
**12**		0	9.18	10.5	2.01	0.48	4.17
**13**	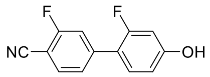	2	10.73	19.6	2.03	0.50	4.06
**14**		1	10.40	12.6	2.02	0.52	3.91
**15**		0	9.96	18.7	2.01	0.52	3.90
**16**	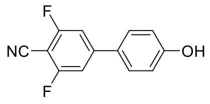	2	10.39	11.8	2.03	0.49	4.13
**17**		1	10.25	4.5	2.02	0.50	4.04
**18**		0	9.66	10.5	2.01	0.50	4.03
**19**	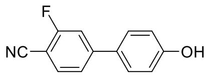	2	9.36	7.5	2.02	0.49	4.10
**20**		1	9.34	2.4	2.02	0.47	4.25
**21**		0	8.64	6.3	2.01	0.48	4.23
**22**	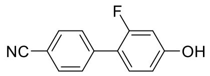	2	9.50	16.6	2.03	0.49	4.13
**23**		1	9.25	8.7	2.02	0.49	4.09
**24**		0	8.72	15.2	2.01	0.49	4.07
**25**	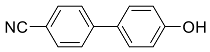	2	8.61	14.0	2.03	0.49	4.13
**26**		1	8.42	5.4	2.02	0.48	4.19
**27**		0	7.86	12.8	2.01	0.48	4.17

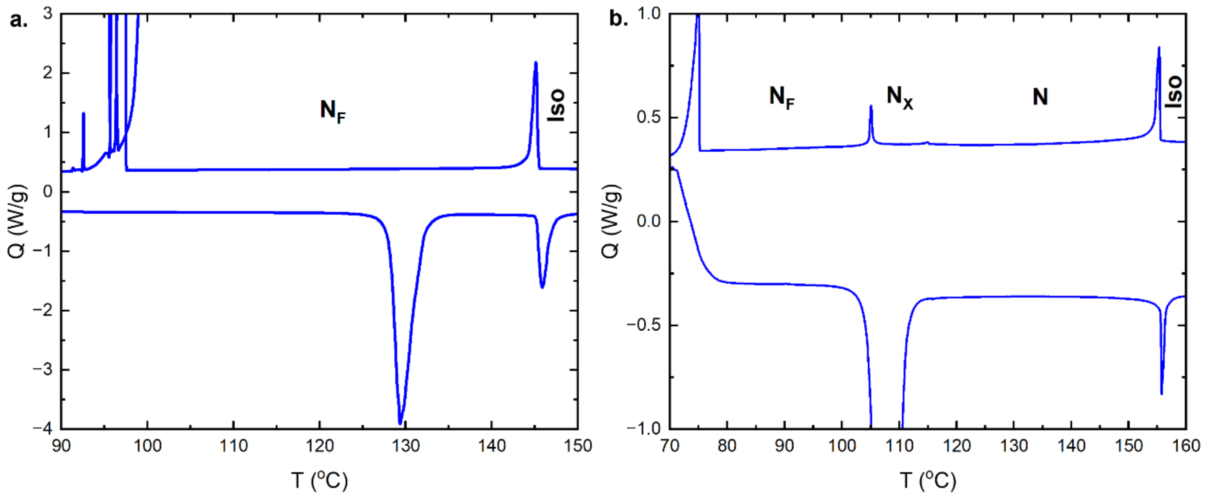

Figure S1. Example DSC traces for (a) **(2)** and (b) **(8)**; the exothermic direction is upwards.

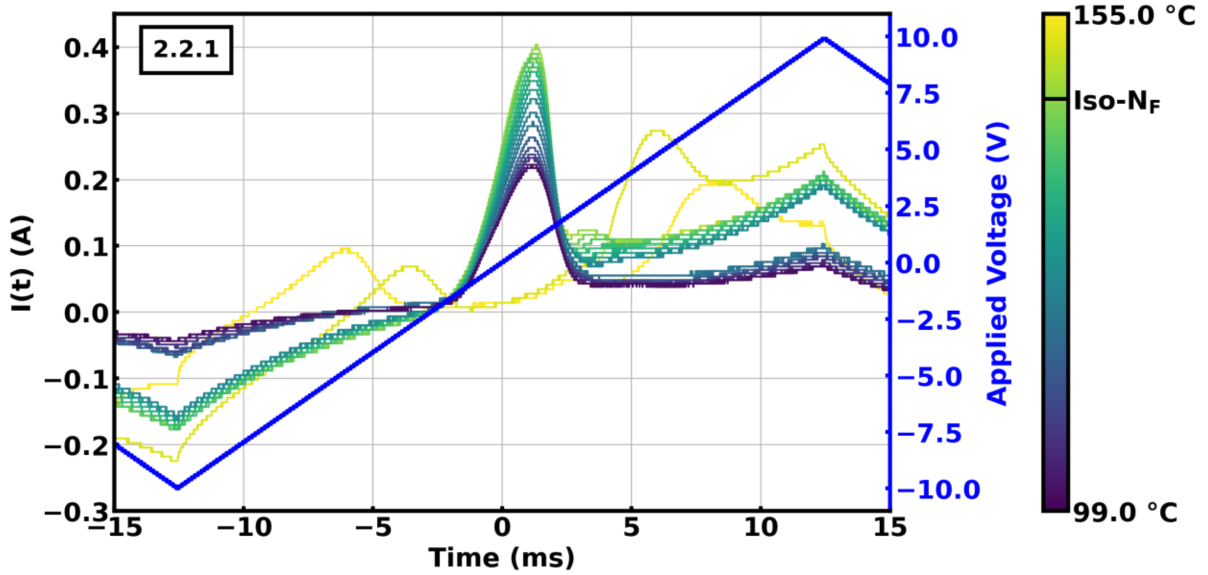

Figure S2. Current responses for compound **2** showing the pre-transitional double peaks (yellow data) associated with the field induced phase transition from the isotropic state to the N_F.

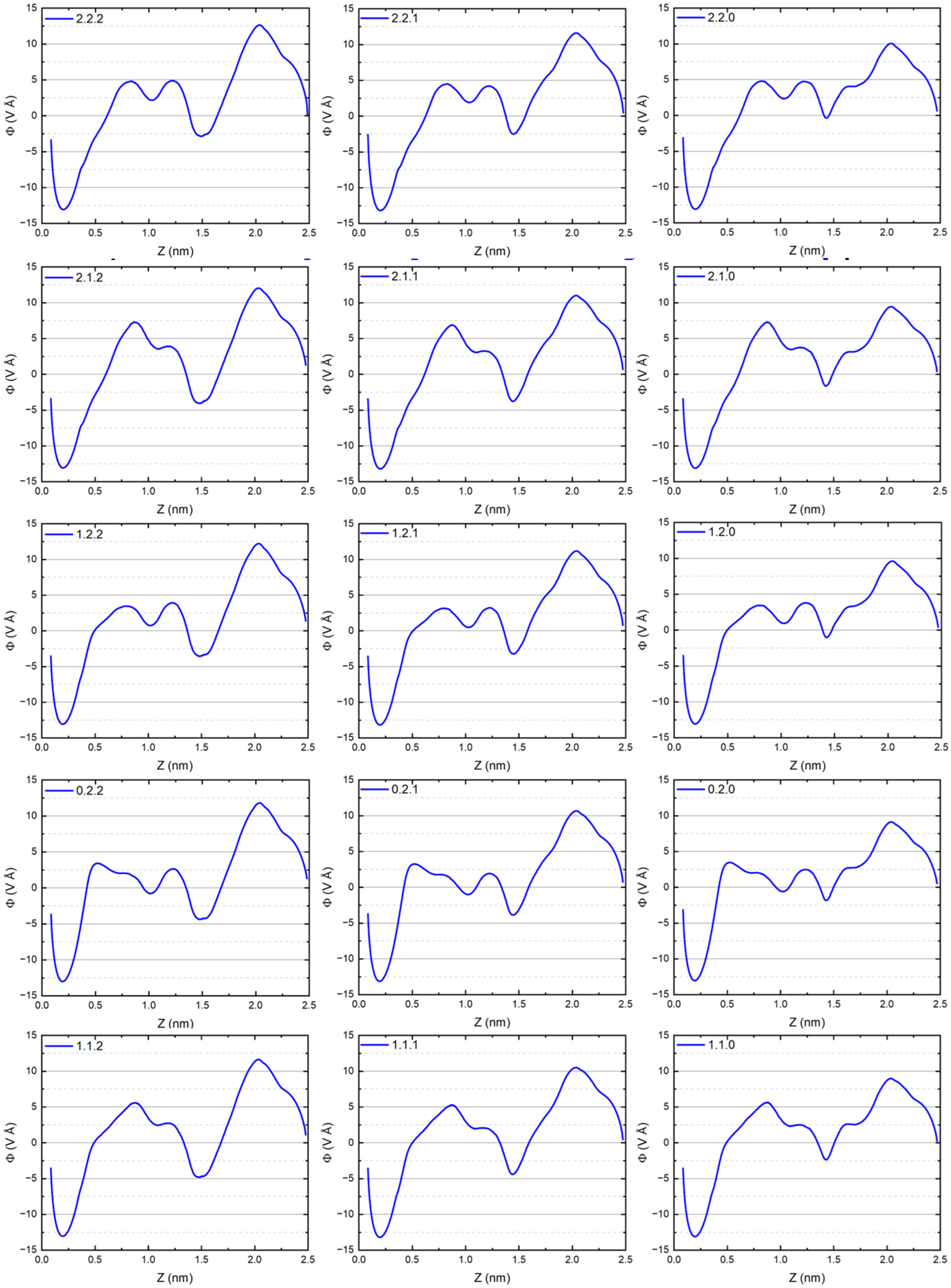
2.2.2
2.2.1
2.2.0
2.1.2
2.1.1
2.1.0
1.2.2
1.2.1
1.2.0
0.2.2
0.2.1
0.2.0
1.1.2
1.1.1
1.1.0
Φ (V Å)
Z (nm)

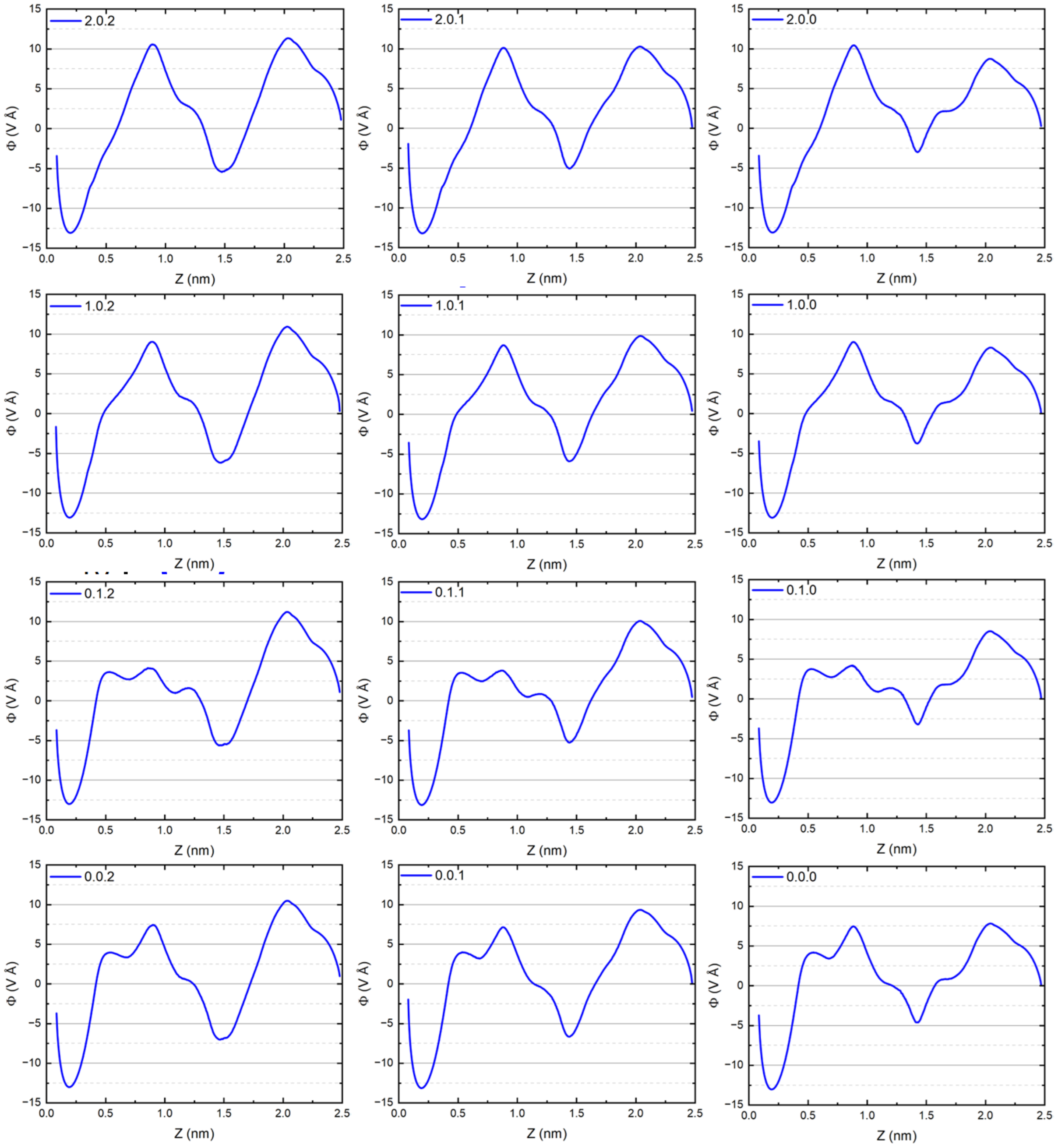

Figure S3. 1D reduced scaled ESP data for materials **1-27**.

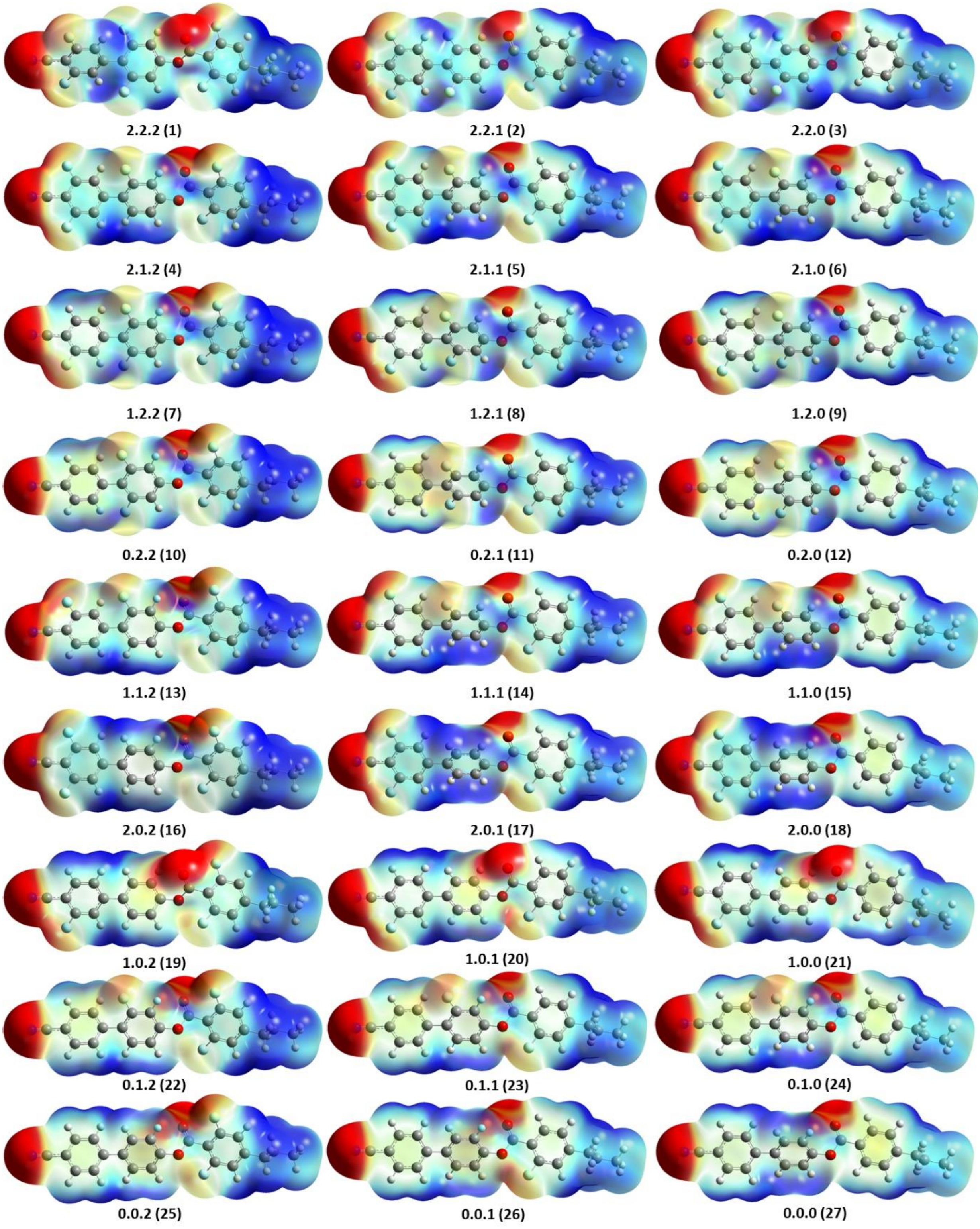

Figure S4 3D ESP data for materials **1-27**. Further blue indicates more positive while further red indicates more negative. Obtained for an electron density isovalue of 0.0004

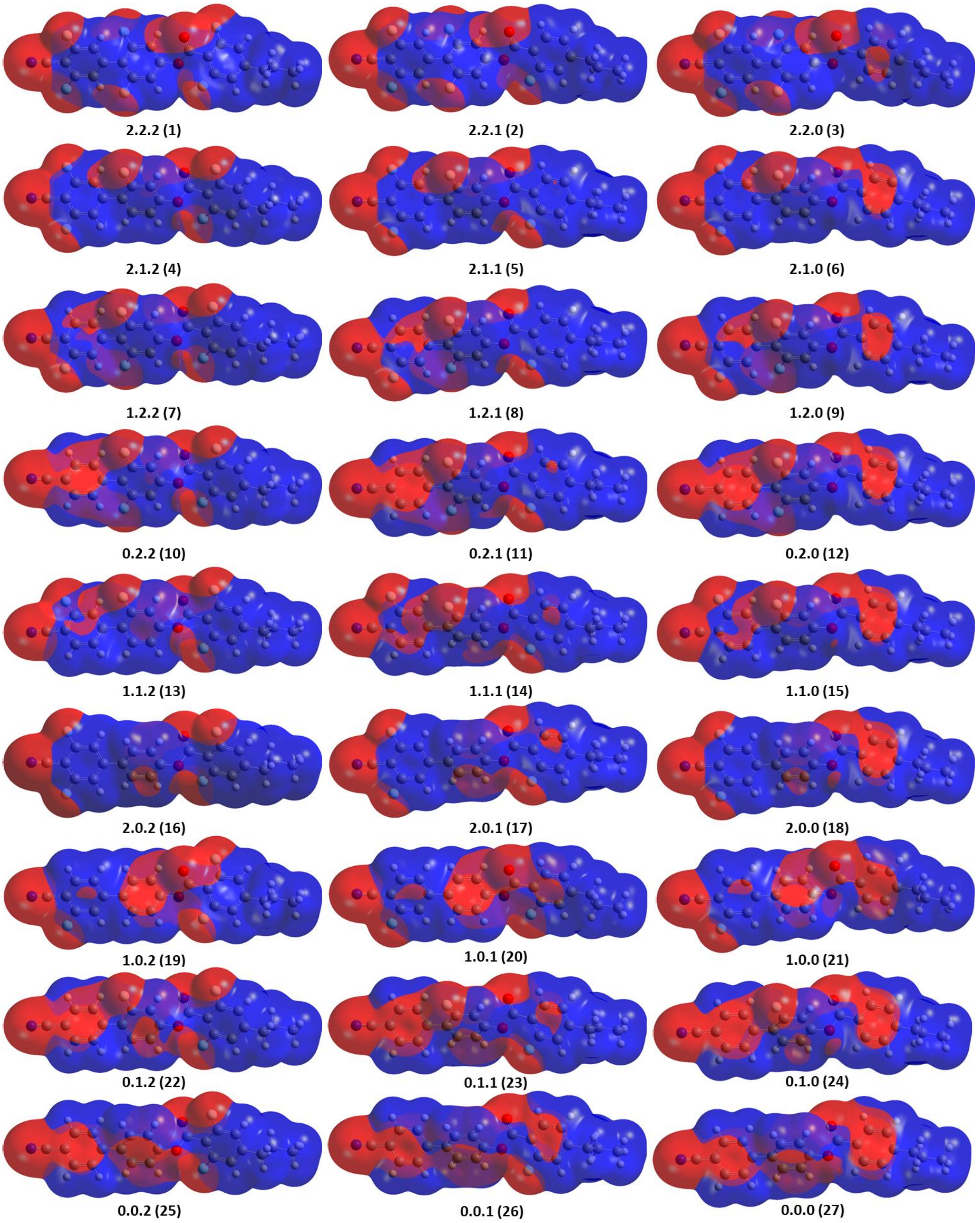

Figure S5. 3D ESP data for materials **1-27**. Obtained for an electron density isovalue of 0.0004. Here all negative regions have been block-coloured red while all the positive regions have been block coloured blue.

3 Organic synthesis

The total synthesis of materials **1-27** is outlined in **Scheme 1** (main manuscript). The synthesis of the 2-fluoro and 2,6- difluoro-4-propyl benzoic acids is described elsewhere [9], and 4-propyl benzoic acid is an article of commerce. The synthesis of some of the fluoro biphenyl phenols have also been described previously within the literature [10–13], and 4-hydroxy-4′-cyanobiphenyl is also an article of commerce.

3.1 Synthesis of 4-(2 fluoro-4-hydroxyphenyl)-2,6-difluorobenzonitrile.

A reaction flask was charged with 4-bromo-2,6-difluorobenzonitrile (8.4 g, 38.5 mmol) and 2-fluoro-4-methoxyphenylboronic acid (7.16 g, 42.3 mmol) which were dissolved in 100 mL of THF and 60 mL of 2M $Na_2CO_{3(aq)}$. The resultant solution was sparged with $N_{2(g)}$ for 20 minutes. In a separate vial, 5 mL of THF was sparged with $N_{2(g)}$ for 15 minutes before $Pd(OAc)_2$ (50 mg) and SPhos (100 mg) were added and stirred for a further 5 minutes. The reaction flask was then heated to 70 ℃ and the catalyst solution added in one portion. The reaction was monitored by TLC with the completion of the reaction being determined by the complete consumption of the bromo-sub-straight ($R_{f\ prod.}$[DCM] = 0.84). The reaction was then cooled, the aqueous and organic layers separated with the organics being dried over $MgSO_4$. The organics were then passed through a silica plug before the filtrate was concentrated under reduced pressure and the product re-crystallised from hexane.

The resultant product (4.5 g, 45%) was then immediately carried forward, dissolved in DCM (conc. ~1M) under an atmosphere of N_2. A solution of BBr_3 (1M in DCM, 30 mL, 30 mmol) was then added dropwise to the stirred solution with the progress of the reaction monitored by TLC ($R_{f\ prod.}$[DCM] ≈ 0). Once complete, the reaction mixture was quenched with water, extracted and dried over $MgSO_4$. The reaction solution was concentrated and purified by flash chromatography over silica gel with a gradient of hexane/ethyl acetate using a Combiflash NextGen300+ system using a gradient elution from hexane - ethyl acetate. The product was then recrystallized from toluene as fine white solid.

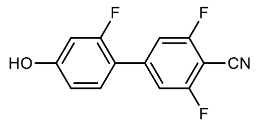

4-(2 fluoro-4-hydroxyphenyl)-2,6-difluorobenzonitrile

R_F (DCM): ≈ 0

^{1}H NMR (400 MHz, DMSO) (δ): 10.63 (s, 1H, Ar-O**H**), 7.57 – 7.45 (m, 3H, Ar-**H**)*, 6.80 – 6.71 (m, 2H, Ar-**H**)*.

*overlapping signals

^{13}C{^{1}H} NMR (101 MHz, DMSO) (δ): 163.97 (d, J = 5.6 Hz), 161.69 (d, J = 251.0 Hz), 161.42 (dd, J = 9.9, 3.0 Hz), 144.68 (t, J = 10.9 Hz), 131.86, 115.34 (d, J = 11.5 Hz), 113.14 (d, J = 2.1 Hz), 112.43 (dt, J = 20.6, 4.0 Hz), 110.17, 103.79 (d, J = 24.8 Hz), 89.27 (t, J = 19.6 Hz).

^{19}F NMR (376 MHz, DMSO) (δ): -106.13 ($d_{apparent}$, J = 10.5 Hz, 2F, Ar-**F**), -114.75 ($t_{apparent}$, J = 11.5 Hz, 1F, Ar-**F**).

3.2 Synthesis of 4-(2,6-difluoro-4-hydroxyphenyl)-2-fluorobenzonitrile

A reaction flask was charged with 4-bromo-2-fluorobenzonitrile (8.0 g, 40 mmol) and 2,6-difluoro-4-methoxyphenylboronic acid (8.4 g, 44 mmol) which were dissolved in 125 mL of THF and 20 mL of 2M $K_2CO_{3(aq)}$. The resultant solution was sparged with $N_{2(g)}$ for 20 minutes. In a separate vial, 5 mL of THF was sparged with $N_{2(g)}$ for 15 minutes before $Pd(OAc)_2$ (20 mg) and SPhos (40 mg) were added and stirred for a further 5 minutes. The reaction flask was then heated to 70 ℃ and the catalyst solution added. The reaction was monitored by TLC with the completion of the reaction being determined by the complete consumption of the bromo-sub-straight ($R_{f\ prod.}$[DCM] = 0.85). The reaction was then cooled, the aqueous and organic layers separated with the organics being dried over $MgSO_4$. The organics were then passed through a silica plug before the filtrate was concentrated under reduced pressure and the product re-crystallised from hexane.

The resultant product (6.5 g, 62%) was then immediately carried forward, dissolved in DCM (conc. ~1M) under an atmosphere of N_2. A solution of BBr_3 (1M in DCM, 30 mL, 30 mmol) was then added dropwise to the stirred solution with the progress of the reaction monitored by TLC ($R_{f\ prod.}$[DCM] ≈ 0). Once complete, the reaction mixture was quenched with water, extracted and dried over $MgSO_4$. The reaction solution was concentrated and purified by flash chromatography over silica gel with a gradient of hexane/ethyl acetate using a Combiflash NextGen300+ system. The product was then recrystallized from toluene as fine white solid.

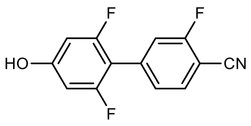

4-(2,6-difluoro-4-hydroxyphenyl)-2-fluorobenzonitrile

R_F (DCM): ≈ 0

^{1}H NMR (400 MHz, DMSO) (δ): 10.78 (s, 1H, Ar-O**H**), 7.99 (dd, J = 8.1, 7.1 Hz, 1H, Ar-**H**), 7.61 (dd, J = 10.6, 1.3 Hz, 1H, Ar-**H**), 7.45 (dd, J = 8.1, 1.5 Hz, 1H, Ar-**H**), 6.74 – 6.50 ($m_{apparent}$, 2H, Ar-**H**).

$^{13}C\{^{1}H\}$ NMR (101 MHz, DMSO) (δ): 163.85, 161.41 (dd, J = 246.0, 8.9 Hz), 161.32, 160.59 (t, J = 15.1 Hz), 137.52 (d, J = 9.2 Hz), 134.14, 127.78, 118.48 (d, J = 20.6 Hz), 114.39, 106.43 (t, J = 18.9 Hz), 100.19 (dd, J = 27.6, 6.4 Hz), 99.70 (d, J = 15.0 Hz).

^{19}F NMR (376 MHz, DMSO) (δ): -108.58 (dd, J = 10.6, 7.0 Hz, 1F, Ar-**F**), -114.55 (d, J = 10.9 Hz, 2F, Ar-**F**).

3.3 ***Synthesis of materials 1-27.***

A round bottomed flask was charged with the appropriate phenol (1 mmol, 1.0 eq), benzoic acid (1.1 mmol, 1.1 eq.), EDC.HCl (1.5 mmol, 1.5 eqv.) and DMAP (~ 2mol%). Dichloromethane was added (conc. ~ 0.1 M) and the suspension stirred until complete consumption of the phenol as judged by TLC. Once complete, the reaction solution was concentrated and purified by flash chromatography over silica gel with a gradient of hexane/DCM using a Combiflash NextGen300+ system. The chromatographed material was dissolved into the minimum quantity of DCM, filtered through a 0.2 micron PTFE filter, concentrated to dryness and finally recrystalised from EtOH.

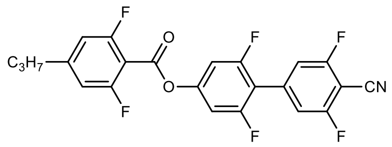

1 (**2.2.2**): *4'-Cyano-2,3',5',6 tetrafluoro-[1,1' biphenyl]4-yl 2,6 difluoro-4-propyl benzoate*

Yield: (Shiny white solid) 323 mg, 72%

R_F (DCM): 0.81

^{1}H NMR (400 MHz, $CDCl_3$) (δ): 7.22 ($d_{apparent}$, J = 9.0 Hz, 2H, Ar-**H**), 7.06 (ddd, J = 8.5, 1.4, 1.4 Hz, 2H, Ar-**H**), 6.87 ($d_{apparent}$, J = 9.8 Hz, 2H, Ar-**H**), 2.65 (t, J = 7.6 Hz, 2H, Ar-C**H₂**-CH₂), 1.69 (q, J = 7.5 Hz, 2H, CH_2-C**H₂**-CH_3), 0.98 (t, J = 7.3 Hz, 3H, CH_2-CH_3).

^{13}C{^{1}H} NMR (101 MHz, $CDCl_3$) (δ): 162.76 (dd, J = 261.6, 5.1 Hz), 160.83 (dd, J = 174.5, 5.9 Hz), 159.16 (dd, J = 168.7, 5.9 Hz), 158.96, 152.17 – 151.45 (m), 136.65 (t, J = 10.7 Hz), f114.23 (dd, J = 21.1, 2.3 Hz), 112.91 (t, J = 17.5 Hz), 112.40 (dd, J = 21.6, 3.2 Hz), 108.95, 106.89 (dd, J = 27.2, 3.0 Hz), 106.13 (t, J = 16.0 Hz), 92.24 (t, J = 19.3 Hz), 37.92, 23.61, 13.55.

^{19}F NMR (376 MHz, $CDCl_3$) (δ): -103.68 (d, J = 8.9 Hz, 2F, Ar-**F**), -108.99 (d, J = 10.4 Hz, 2F, Ar-**F**), -111.66 (d, J = 9.3 Hz, 2F, Ar-**F**).

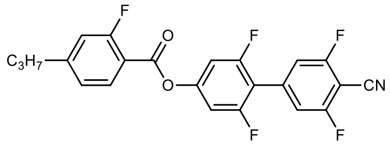

2 (**2.2.1**): *4'-Cyano-2,3',5',6 tetrafluoro-[1,1' biphenyl]4-yl 2 fluoro-4-propyl benzoate*

Yield: (Shiny white solid) 215 mg, 50%

R_F (DCM): 0.83

^{1}H NMR (400 MHz, $CDCl_3$) (δ): 7.99 (t, J = 7.8 Hz, 1H, Ar-**H**), 7.21 (ddd, J = 8.1, 1.8, 1.2 Hz, 2H, Ar-**H**), 7.11 (dd, J = 8.1, 1.6 Hz, 1H, Ar-**H**), 7.08 – 7.01 (m, 3H, Ar-**H**)*, 2.68 (t, J = 6.8 Hz, 2H, Ar-C**H₂**-CH₂), 1.70 (h, J = 7.4 Hz, 2H, CH_2-C$\mathbf{H_2}$-CH_3), 0.98 (t, J = 7.3 Hz, 3H, CH_2-C$\mathbf{H_3}$).

*Overlapping signals

$^{13}C\{^{1}H\}$ NMR (101 MHz, $CDCl_3$) (δ): 164.14 (dd, J = 261.0, 5.3 Hz), 162.05 (d, J = 262.2 Hz), 161.70 (d, J = 4.3 Hz), 159.57 (dd, J = 251.9, 8.4 Hz), 152.83 (d, J = 8.7 Hz), 152.32 (t, J = 14.4 Hz), 136.76 (t, J = 10.7 Hz), 132.44, 124.65 (d, J = 3.3 Hz), 117.16 (d, J = 21.7 Hz), 114.27 (dd, J = 21.0, 2.8 Hz), 108.98, 106.97 (dd, J = 27.0, 3.1 Hz), 92.18 (t, J = 19.3 Hz), 37.89, 23.85, 13.65.

^{19}F NMR (376 MHz, $CDCl_3$) (δ): -103.73 (d, J = 9.0 Hz, 2F, Ar-**F**), -107.90 (dd, J = 11.8, 7.5 Hz, 1F, Ar-**F**), -111.94 (d, J = 9.4 Hz, 2F, Ar-**F**).

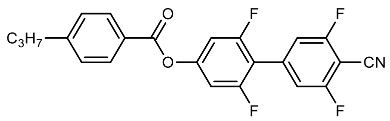

3 (**2.2.0**): *4'-Cyano-2,3',5',6 tetrafluoro-[1,1' biphenyl]4-yl 4-propyl benzoate*

Yield: (white solid) 281 mg, 68%

R_F (DCM): 0.87

^{1}H NMR (400 MHz, $CDCl_3$) (δ): 8.09 (ddd, J = 8.4, 1.8, 1.8 Hz, 2H, Ar-**H**), 7.34 (ddd, J = 8.4, 1.9, 1.9 Hz, 2H, Ar-**H**), 7.22 (ddd, J = 8.2, 1.6, 1.6 Hz, 2H, Ar-**H**), 7.06 – 6.99 (m, 2H, Ar-**H**), 2.70 (t, J = 7.3 Hz, 2H, Ar-C$\mathbf{H_2}$-CH_2), 1.70 (h, J = 7.5 Hz, 2H, CH_2-C$\mathbf{H_2}$-CH_3), 0.97 (t, J = 7.4 Hz, 3H, CH_2-C$\mathbf{H_3}$).

$^{13}C\{^{1}H\}$ NMR (101 MHz, $CDCl_3$) (δ): 164.24 – 164.07 (m), 161.53 (d, J = 5.1 Hz), 159.60 (dd, J = 252.0, 8.4 Hz), 152.82 (t, J = 14.5 Hz), 150.13, 136.86 (t, J = 10.5 Hz), 130.44, 128.99, 125.72, 114.27 (dd, J = 21.0, 3.0 Hz), 108.99, 107.25 – 106.73 (m), 92.17 (t, J = 19.3 Hz), 38.16, 24.22, 13.74.

^{19}F NMR (376 MHz, $CDCl_3$) (δ): -103.72 (d, J = 8.9 Hz, 2F, Ar-**F**), -112.01 (d, J = 9.5 Hz, 2F, Ar-**F**).

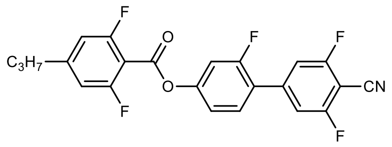

4 (**2.1.2**): 4’-Cyano-2,3’,6 trifluoro-[1,1’ biphenyl]4-yl 2,6 difluoro-4-propyl benzoate

Yield: (white powder) 319 mg, 74%

R_F (DCM): 0.83

^{1}H NMR (400 MHz, $CDCl_3$) (δ): 7.41 (t, *J* = 8.6 Hz, 1H, Ar-**H**), 7.26 – 7.08 (m, 5H, Ar-H)*, 6.80 ($d_{apparent}$, *J* = 10.1 Hz, 2H, Ar-**H**), 2.58 (t, *J* = 7.6 Hz, 2H, Ar-C**H₂**-CH₂), 1.61 (h, *J* = 7.4 Hz, 2H, CH₂-C**H₂**-CH₃), 0.90 (t, *J* = 7.3 Hz, 3H, CH₂-C**H₃**).

*Overlapping signals

^{13}C{^{1}H} NMR (101 MHz, $CDCl_3$) (δ): 164.37 (dd, J = 185.8, 5.2 Hz), 161.80, 161.44 (dd, J = 183.0, 5.5 Hz), 158.81 (d, J = 115.0 Hz), 152.21 (d, J = 11.1 Hz), 151.33 (t, J = 9.9 Hz), 143.04 (t, J = 9.7 Hz), 130.59 (d, J = 3.7 Hz), 123.19 (d, J = 12.8 Hz), 118.61 (d, J = 3.7 Hz), 112.75 (t, J = 3.9 Hz), 112.61 – 112.36 (m)*, 112.23 (d, J = 3.0 Hz) 111.02 (d, J = 25.9 Hz), 109.11, 106.53 (t, J = 16.3 Hz), 91.49 (t, J = 19.1 Hz), 37.90, 23.63, 13.56.

*Overlapping signals

^{19}F NMR (376 MHz, $CDCl_3$) (δ): -103.50 (d, J = 9.3 Hz, 2F, Ar-**F**), -109.27 (d, J = 10.4 Hz, 2F, Ar-**F**), -113.29 ($t_{apparent}$, J = 9.9 Hz, 1F, Ar-**F**).

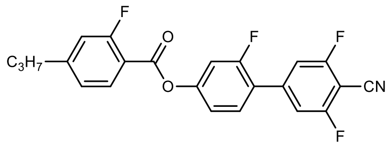

5 (**2.1.1**): 4’-Cyano-2,3’,6 trifluoro-[1,1’ biphenyl]4-yl 2 fluoro-4-propyl benzoate

Yield: (shiny white solid) 272 mg, 66%

R_F (DCM): 0.82

^{1}H NMR (400 MHz, $CDCl_3$) (δ): 8.00 (t, J = 7.8 Hz, 1H, Ar-**H**), 7.48 (t, J = 8.6 Hz, 1H, Ar-**H**), 7.32 – 7.27 (m, 1H, Ar-**H**)$^+$, 7.25 – 7.17 (m, 2H, Ar-**H**), 7.11 (dd, J = 8.1, 1.6 Hz, 1H, Ar-**H**), 7.05 (dd, J = 11.9, 1.6 Hz, 1H, Ar-**H**), 2.68 (t, J = 7.6 Hz, 2H, Ar-C**H₂**-CH₂), 1.70 (h, J = 7.4 Hz, 2H, CH₂-C**H₂**-CH₃), 0.98 (t, J = 7.3 Hz, 3H, CH₂-C**H₃**).

$^+$Overlapping with solvent peak.

$^{13}C\{^1H\}$ NMR (101 MHz, $CDCl_3$) (δ): 162.68 (dd, J = 259.0, 25.7 Hz), 161.80 (d, J = 4.1 Hz), 159.67 (dd, J = 251.2, 8.5 Hz), 152.74 (d, J = 8.6 Hz), 151.87 (t, J = 14.3 Hz), 136.02 (d, J = 8.6 Hz), 133.24, 132.43, 126.88 (d, J = 2.7 Hz), 124.62 (d, J = 3.2 Hz), 118.49 (d, J = 20.9 Hz), 117.15 (d, J = 21.7 Hz), 114.14 (d, J = 9.3 Hz), 113.72, 113.44, 106.82 (dd, J = 27.5, 2.6 Hz), 101.24 (d, J = 15.4 Hz), 77.36, 77.24, 77.04, 76.72, 37.88, 23.86, 13.65.

^{19}F NMR (376 MHz, $CDCl_3$) (δ): -103.52 (d, J = 9.3 Hz, 2F, Ar-**F**), -108.16 (dd, J = 12.0, 7.5 Hz, 1F, Ar-**F**), -113.48 (t, J = 9.9 Hz, 1F, Ar-**F**).

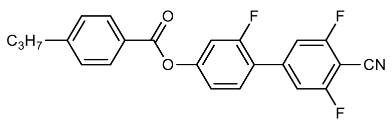

6 (**2.1.0**): *4'-Cyano-2,3',6 trifluoro-[1,1' biphenyl]4-yl 4-propyl benzoate*

Yield: (white solid) 296 mg, 75%

R_F (DCM): 0.90

^1H NMR (400 MHz, $CDCl_3$) (δ): (d $_{(apparent)}$, J = 8.1 Hz, 2H, Ar-**H**), 7.48 (t, J = 8.6 Hz, 1H, Ar-**H**), 7.34 (d $_{(apparent)}$, J = 8.0 Hz, 2H, Ar-**H**), 7.30 – 7.24 (m, 2H, Ar-**H**)$^{*+}$, 7.23 – 7.14 (m, 1H, Ar-**H**), 2.70 (t, J = 7.6 Hz, 2H, Ar-C**H_2**-CH_2), 1.70 (h, J = 7.4 Hz, 2H, CH_2-C**H_2**-CH_3), 0.97 (t, J = 7.3 Hz, 3H, CH_2-C**H_3**).

*Overlapping signals, $^+$overlapping solvent peak.

$^{13}C\{^1H\}$ NMR (101 MHz, $CDCl_3$) (δ): 164.59, 163.11 (dd, J = 260.5, 4.9 Hz), 161.81, 158.29, 153.04 (d, J = 11.2 Hz), 149.87, 143.19 (t, J = 9.9 Hz), 130.50 (d, J = 3.6 Hz), 130.39, 128.92, 126.11, 118.76 (d, J = 3.7 Hz), 112.60 (dt, J = 20.9, 3.7 Hz), 111.14 (d, J = 25.5 Hz), 109.15, 38.16, 24.24, 13.75.

^{19}F NMR (376 MHz, $CDCl_3$) (δ): -103.55 (d, J = 9.3 Hz, 2F, Ar-**F**), -113.55 (t, J = 9.9 Hz, 1F, Ar-**F**).

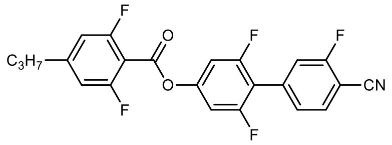

7 (**1.2.2**): 4'-Cyano-2,3',5' trifluoro-[1,1' biphenyl]4-yl 2,6 difluoro-4-propyl benzoate

Yield: (shiny white solid) 258 mg, 60%

R_F (DCM): 0.82

^{1}H NMR (400 MHz, $CDCl_3$) (δ): 7.66 (t, J = 6.9 Hz, 1H, Ar-**H**), 7.36 – 7.27 (m, 2H, Ar-**H**)*, 7.01 – 6.93 (m, 2H, Ar-**H**), 6.84 – 6.75 (m, 2H, Ar-**H**), 2.58 (t, J = 7.4 Hz, 2H, Ar-C**H₂**-CH₂), 1.61 (h, J = 7.5 Hz, 2H, CH₂-C**H₂**-CH₃), 0.90 (t, J = 7.3 Hz, 3H, CH₂-C**H₃**).

*overlapping signals

^{13}C{^{1}H} NMR (101 MHz, $CDCl_3$) (δ): 164.11 (d, J = 260.0 Hz), 162.57 (dd, J = 157.7, 5.8 Hz), 161.13 (d, J = 247.9 Hz), 159.18 (dd, J = 150.5, 5.7 Hz), 151.83 – 151.23 (m)*, 135.91 (d, J = 8.6 Hz), 133.26, 126.87, 118.50 (d, J = 21.0 Hz), 113.71, 112.38 (dd, J = 21.6, 3.3 Hz), 106.74 (dd, J = 27.7, 2.7 Hz), 101.32 (d, J = 15.4 Hz), 37.92, 23.62, 13.56.

*overlapping signals

^{19}F NMR (376 MHz, $CDCl_3$) (δ): -106.10 (t, J = 6.6 Hz, 1F, Ar-**F**), -109.07 (d, J = 10.4 Hz, 2F, Ar-**F**), -111.96 (d, J = 9.0 Hz, 2F, Ar-**F**).

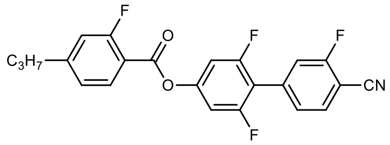

8 (**1.2.1**): 4'-Cyano-2,3',5' trifluoro-[1,1' biphenyl]4-yl 2 fluoro-4-propyl benzoate

Yield: (white solid) 318 mg, 77%

R_F (DCM): 0.82

^{1}H NMR (400 MHz, $CDCl_3$) (δ): 7.99 (t, J = 7.8 Hz, 1H, Ar-**H**), 7.72 (t, J = 6.6 Hz, 1H, Ar-**H**), 7.44 – 7.34 (m, 2H, Ar-**H**), 7.11 (dd, J = 8.1, 1.6 Hz, 1H, Ar-**H**), 7.08 – 6.98 (m, 3H, Ar-**H**)*, 2.68 (t, J = 6.8 Hz, 2H, Ar-C**H₂**-CH₂), 1.70 (h, J = 7.3 Hz, 2H, CH₂-C**H₂**-CH₃), 0.97 (t, J = 7.3 Hz, 3H, C**H₂**-CH₃).

*Overlapping signals

^{13}C{^{1}H} NMR (101 MHz, $CDCl_3$) (δ): 162.68 (dd, J = 259.0, 25.7 Hz), 161.80 (d, J = 4.1 Hz), 159.67 (dd, J = 251.2, 8.5 Hz), 152.74 (d, J = 8.6 Hz), 151.87 (t, J = 14.3 Hz), 136.02 (d, J = 8.6 Hz), 133.24, 132.43, 126.88 (d, J = 2.7 Hz), 124.62 (d, J = 3.2 Hz), 118.49 (d, J = 20.9 Hz), 117.15 (d, J = 21.7 Hz), 114.14 (d, J = 9.3 Hz), 106.82 (dd, J = 27.5, 2.6 Hz), 101.24 (d, J = 15.4 Hz), 37.88, 23.86, 13.65.

^{19}F NMR (376 MHz, $CDCl_3$) (δ): -106.17 (t, J = 6.8 Hz, 1F, Ar-**H**), -107.97 (dd, J = 11.9, 7.6 Hz, 1F, Ar-**H**), -112.25 (d, J = 9.2 Hz, 2F, Ar-**H**).

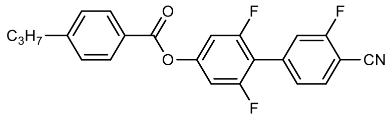

9 (**1.2.0**): 4'-Cyano-2,3',5' trifluoro-[1,1' biphenyl]4-yl 4-propyl benzoate

Yield: (white solid) 277 mg, 70%

R_F (DCM): 0.85

^{1}H NMR (400 MHz, $CDCl_3$) (δ): 8.09 (ddd, J = 8.4, 2.0, 1.8 Hz, 2H, Ar-**H**), 7.72 (t, J = 7.0 Hz, 1H, Ar-**H**), 7.45 – 7.36 (m, 2H, Ar-**H**), 7.35 (ddd, J = 8.6, 1.8, 1.6 Hz, 2H, Ar-**H**), 7.06 – 6.97 (m, 2H, Ar-**H**), 2.70 (t, J = 7.4 Hz, 2H, Ar-C**H_2**-CH_3), 1.70 (h, J = 7.4 Hz, 2H, CH_2-C**H_2**-CH_3), 0.97 (t, J = 7.3 Hz, 3H, CH_2-C**H_3**).

^{13}C{^{1}H} NMR (101 MHz, $CDCl_3$) (δ): 164.21 (d, J = 18.0 Hz), 161.54, 160.90 (dd, J = 242.2, 8.4 Hz), 152.29 (t, J = 14.2 Hz), 150.05, 136.12 (d, J = 8.9 Hz), 133.24, 130.43, 128.97, 126.88, 125.83, 118.50 (d, J = 21.7 Hz), 113.74, 106.85 (dd, J = 28.2, 2.4 Hz), 101.31, 38.16, 24.22, 13.74.

^{19}F NMR (376 MHz, $CDCl_3$) (δ): -106.15 (t, J = 8.0 Hz, 1F, Ar-**F**), -112.33 (d, J = 9.1 Hz, 2F, Ar-**F**).

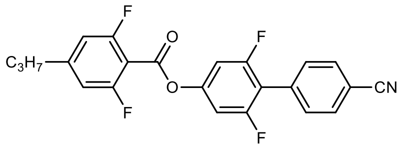

10 (**0.2.2**): *4'-Cyano-3',5' difluoro-[1,1' biphenyl]4-yl 2,6 difluoro-4-propyl benzoate*

Yield: (fluffy white solid) 335 mg, 80%

R_F (DCM): 0.79

^{1}H NMR (400 MHz, $CDCl_3$) (δ): 7.76 (ddd, J = 8.2, 1.7, 1.5 Hz, 2H, Ar-**H**), 7.60 (ddd, J = 8.4, 1.5, 1.5 Hz, 3H, Ar-**H**), 7.07 – 6.98 (m, 2H, Ar-**H**), 6.87 (d $_{\text{(apparent)}}$, J = 9.9 Hz, 2H, Ar-**H**), 2.65 (t, J = 7.4 Hz, 2H, Ar-C**H_2**-CH_2), 1.69 (h, J = 7.6 Hz, 2H, CH_2-C**H_2**-CH_3), 0.97 (t, J = 7.3 Hz, 3H, CH_2-C**H_3**).

^{13}C{^{1}H} NMR (101 MHz, $CDCl_3$) (δ): 161.78 (dd, J = 154.0, 7.4 Hz), 159.23 (dd, J = 137.9, 6.1 Hz), 159.16, 158.57, 158.48, 151.45 (t, J = 9.7 Hz), 150.91 (t, J = 14.4 Hz), 133.42, 132.12, 131.11, 118.57, 114.82 (t, J = 18.4 Hz), 112.47 (d, J = 3.2 Hz), 112.25 (d, J = 3.2 Hz), 106.98 – 106.24 (m)*, 37.91, 23.62, 13.56.

*overlapping signals.

^{19}F NMR (376 MHz, $CDCl_3$) (δ): -109.14 (d, J = 10.4 Hz, 2F, Ar-**F**), -112.28 (d, J = 8.8 Hz, 2F, Ar-**F**).

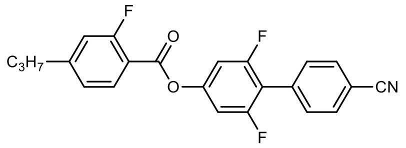

11 (**0.2.1**): 4'-Cyano-3',5' difluoro-[1,1' biphenyl]4-yl 2 fluoro-4-propyl benzoate

Yield: (white powder) 213 mg, 54%

R_F (DCM): 0.84

^1H NMR (400 MHz, $CDCl_3$) (δ): 7.99 (t, J = 7.8 Hz, 1H, Ar-**H**), 7.76 (ddd, J = 8.6, 1.9, 1.6 Hz, 2H, Ar-**H**), 7.60 (ddd, J = 8.6, 1.4, 1.4 Hz, 2H, Ar-**H**), 7.11 (dd, J = 8.0, 1.6 Hz, 1H, Ar-**H**), 7.04 (dd, J = 12.0, 1.4 Hz, 1H, Ar-**H**), 7.02 – 6.97 (m, 2H, Ar-**H**), 2.68 (t, J = 6.8 Hz, 2H, Ar-C$\mathbf{H_2}$-CH_2), 1.70 (h, J = 7.3 Hz, 2H, CH_2-C$\mathbf{H_2}$-CH_3), 0.98 (t, J = 7.4 Hz, 3H, CH_2-CH_3).

$^{13}C\{^1H\}$ NMR (101 MHz, $CDCl_3$) (δ): 163.84 (d, J = 261.7 Hz), 161.92 (d, J = 3.8 Hz), 160.97, 159.77 (dd, J = 250.5, 8.8 Hz), 152.63 (d, J = 8.7 Hz), 151.39 (t, J = 14.3 Hz), 133.52, 132.44, 132.11, 131.12, 124.60 (d, J = 3.3 Hz), 118.59, 117.25, 117.03, 114.25 (d, J = 9.2 Hz), 112.21, 106.94 – 106.44 (m)., 37.88, 23.86, 13.66.

^{19}F NMR (376 MHz, $CDCl_3$) (δ): -108.03 (dd, J = 12.0, 7.5 Hz, 1F, Ar-**F**), -112.56 (d, J = 8.8 Hz, 2F, Ar-**F**).

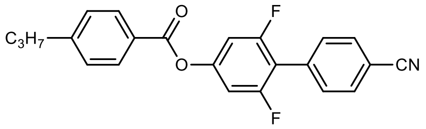

12 (**0.2.0**): *4'-Cyano-3',5' difluoro-[1,1' biphenyl]4-yl 4-propyl benzoate*

Yield: (fluffy white solid) 230 mg, 61%

R_F (DCM): 0.89

^1H NMR (400 MHz, $CDCl_3$) (δ): 8.10 (ddd, J = 8.4, 1.7, 1.6 Hz, 2H, Ar-**H**), 7.76 (ddd, J = 8.7, 1.8, 1.4 Hz, 2H, Ar-**H**), 7.60 (ddd, J = 8.5, 1.4, 1.2 Hz, 2H, Ar-**H**), 7.35 (ddd, J = 8.5, 1.8, 1.6 Hz, 2H, Ar-**H**), 7.04 – 6.93 ($m_{apparent}$, 2H, Ar-**H**), 2.70 (t, J = 7.3 Hz, 2H, Ar-C$\mathbf{H_2}$-CH_2), 1.70 (h, J = 7.5 Hz, 2H, CH_2-C$\mathbf{H_2}$-CH_3), 0.97 (t, J = 7.3 Hz, 3H, CH_2-C$\mathbf{H_3}$).

$^{13}C\{^1H\}$ NMR (101 MHz, $CDCl_3$) (δ): 164.37, 159.79 (dd, J = 250.3, 8.9 Hz), 151.83 (t, J = 14.3 Hz), 149.97, 133.57, 132.10, 131.12, 130.42, 128.95, 125.94, 118.60, 114.37 (t, J = 18.5 Hz), 112.19, 107.04 – 106.42 ($m_{apparent}$), 38.16, 24.23, 13.75.

^{19}F NMR (376 MHz, $CDCl_3$) (δ): -112.65 (d, J = 8.8 Hz, 2F, Ar-**F**).

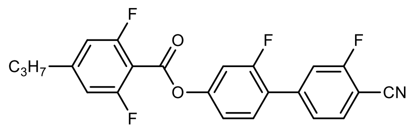

13 (**1.1.2**): 4'-Cyano-2,3' difluoro-[1,1' biphenyl]4-yl 2,6 difluoro-4-propyl benzoate

Yield: (white solid) 198 mg, 48%

R_F (DCM): 0.83

^1H NMR (400 MHz, $CDCl_3$) (δ): 7.71 (t, J = 7.1 Hz, 1H, Ar-**H**), 7.53 – 7.40 ($m_{apparent}$, 3H, Ar-**H**)*, 7.24 – 7.16 ($m_{apparent}$, 2H)*, 6.91 – 6.84 ($m_{apparent}$, 2H), 2.65 (t, J = 7.4 Hz, 2H, Ar-C**H₂**-CH₂), 1.68 (h, J = 7.4 Hz, 2H, CH_2-C$\mathbf{H_2}$-CH_3), 0.97 (t, J = 7.3 Hz, 3H, CH_2-C$\mathbf{H_3}$).

*overlapping signals.

$^{13}C\{^1H\}$ NMR (101 MHz, $CDCl_3$) (δ): 164.35, 161.77 (dd, J = 258.6, 6.1 Hz), 161.7, 159.47 (d, J = 252.6 Hz), 159.4, 151.75 (d, J = 11.1 Hz), 151.22 (t, J = 9.9 Hz), 142.29 (d, J = 8.4 Hz), 130.77 (d, J = 4.0 Hz), 125.33 (t, J = 3.3 Hz), 124.08 (d, J = 12.8 Hz), 118.42 (d, J = 3.7 Hz), 116.92 (dd, J = 20.7, 3.9 Hz), 113.85, 112.32 (dd, J = 21.7, 3.2 Hz), 110.85 (d, J = 25.8 Hz), 106.64, 100.60 (d, J = 15.6 Hz), 37.89, 23.63, 13.56.

^{19}F NMR (376 MHz, $CDCl_3$) (δ): -106.08 ($t_{apparent}$, J = 7.9 Hz, 1F, Ar-**F**), -109.34 (d, J = 10.3 Hz, 2F, Ar-**F**), -113.76 ($t_{apparent}$, J = 9.8 Hz, 1F, Ar-**F**).

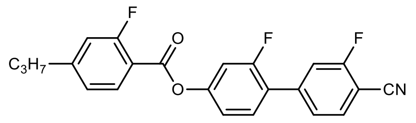

14 (**1.1.1**): *4'-Cyano-2,3' difluoro- [1,1' biphenyl]4-yl 2 fluoro-4-propyl benzoate*

Yield: (shiny white solid) 245 mg, 62%

R_F (DCM): 0.85

^1H NMR (400 MHz, $CDCl_3$) (δ): 7.94 (t, J = 8.3 Hz, 1H, Ar-**H**), 7.64 (t, J = 7.2 Hz, 1H, Ar-**H**), 7.39 (m, 3H, Ar-**H**)*, 7.12 (m, 2H, Ar-**H**)*, 7.04 ($d_{apparent}$, J = 7.9 Hz, 1H, Ar-**H**), 6.98 ($d_{apparent}$, J = 11.8 Hz, 1H, Ar-**H**), 2.61 (t, J = 7.6 Hz, 2H, Ar-C$\mathbf{H_2}$-CH_2), 1.63 (h, J = 7.4 Hz, 2H, CH_2-C$\mathbf{H_2}$-CH_3), 0.91 (t, J = 7.3 Hz, 3H, CH_2-C$\mathbf{H_3}$).

^{19}F NMR (376 MHz, $CDCl_3$) (δ): -104.73 (t, J = 9.4 Hz, 1F, Ar-**F**), -108.23 (t, J = 10.1 Hz, 1F, Ar-**F**), -113.96 (t, J = 10.3 Hz, 1F, Ar-**F**).

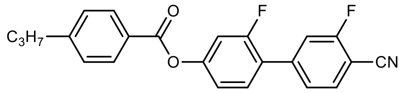

15 (**1.1.0**): 4’-Cyano-2,3’ difluoro-[1,1’ biphenyl]4-yl-4-propyl benzoate

Yield: (white solid) 204 mg,54 %

R_F (DCM): 0.91

^{1}H NMR (400 MHz, $CDCl_3$) (δ): 8.11 (ddd, J = 8.3, 1.9, 1.7 Hz, 2H, Ar-**H**), 7.71 (t, J = 6.9 Hz, 1H, Ar-**H**), 7.52 – 7.41 (m, 3H, Ar-**H**)*, 7.34 (ddd, J = 8.4, 1.6, 1.5 Hz, 2H, Ar-**H**), 7.20 – 7.13 (m, 2H, Ar-**H**)*, 2.70 (t, J = 6.8 Hz, 2H, Ar-C**H₂**-CH₂), 1.70 (h, J = 7.4 Hz, 2H, CH₂-C**H₂**-CH₃), 0.97 (t, J = 7.3 Hz, 3H, CH₂-CH₃).

*overlapping signals.

^{13}C{^{1}H} NMR (101 MHz, $CDCl_3$) (δ): 164.68, 163.08 (d, J = 259.0 Hz), 159.56 (d, J = 251.9 Hz), , 152.56 (d, J = 11.2 Hz), 149.78, 142.45 (d, J = 8.4 Hz), 133.47, 130.68 (d, J = 4.0 Hz), 130.38, 128.90, 126.21, 125.31 ($t_{apparent}$, J = 3.5 Hz), 123.64 (d, J = 12.8 Hz), 118.58 (d, J = 3.7 Hz), 116.99 (d, J = 3.4 Hz), 116.80 (d, J = 3.5 Hz), 113.89, 111.10, 110.85, 100.52 (d, J = 15.7 Hz), 38.16, 24.24, 13.75.

^{19}F NMR (376 MHz, $CDCl_3$) (δ): -106.09 ($t_{apparent}$, J = 8.1 Hz, 1F, Ar-**F**), -114.02 ($t_{apparent}$, J = 9.7 Hz, 1F, Ar-**F**).

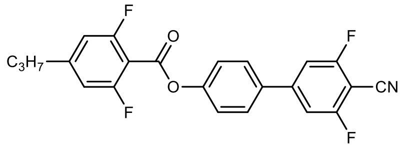

16 (**2.0.2**): *4’-Cyano-3’,5’ difluoro-[1,1’ biphenyl]4-yl 2,6 difluoro-4-propyl benzoate*

Yield: (fluffy white solid) 314 mg, 76%

R_F (DCM): 0.82

^{1}H NMR (400 MHz, $CDCl_3$) (δ): 7.63 ($d_{apparent}$, J = 8.2 Hz, 2H, Ar-**H**), 7.40 ($d_{apparent}$, J = 8.4 Hz, 2H, Ar-**H**), 7.32 – 7.23 ($m_{apparent}$, 2H, Ar-**H**)$^+$, 6.87 ($d_{apparent}$, J = 10.1 Hz, 2H, Ar-**H**), 2.65 (t, J = 7.6 Hz, 2H, Ar-C**H₂**-CH₂), 1.69 (h, J = 7.4 Hz, 2H, CH₂-C**H₂**-CH₃), 0.98 (t, J = 7.3 Hz, 3H, CH₂-C**H₃**).

$^+$overlapping with solvent peak

^{13}C{^{1}H} NMR (101 MHz, $CDCl_3$) (δ): 164.71 (dd, J = 261.5, 5.3 Hz), 162.61 – 159.66 (m)*, 151.77, 150.95 ($t_{apparent}$, J = 9.8 Hz), 148.40 ($t_{apparent}$, J = 10.6 Hz), 135.13, 128.40, 122.75, 112.27 (dd, J = 24.1, 2.6 Hz), 110.61 (dd, J = 20.1, 3.4 Hz), 109.27, 106.96 ($t_{apparent}$, J = 16.5 Hz), 90.90 ($t_{apparent}$, J = 19.9 Hz), 37.88, 23.65, 13.56.

*overlapping signals.

^{19}F NMR (376 MHz, $CDCl_3$) (δ): -103.23 ($d_{apparent}$, J = 9.5 Hz, 2F, Ar-**F**), -109.57 ($d_{apparent}$, J = 10.2 Hz, 2F, Ar-**F**).

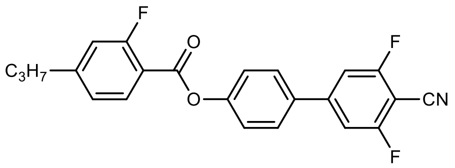

17 (**2.0.1**): *4'-Cyano-3',5' difluoro-[1,1' biphenyl]4-yl 2 fluoro-4-propyl benzoate*

Yield: (white solid) 225 mg, 57%

R_F (DCM): 0.83

^{1}H NMR (400 MHz, $CDCl_3$) (δ): 7.93 (t, J = 7.8 Hz, 1H, Ar-**H**), 7.54 (ddd, J = 8.8, 2.7, 2.1 Hz, 2H, Ar-**H**), 7.29 (ddd, J = 8.8, 3.0, 2.3 Hz, 2H, Ar-**H**), 7.19 (ddd, J = 8.0, 1.8, 1.8 Hz, 2H, Ar-**H**), 7.02 (dd, J = 8.1, 1.6 Hz, 1H, Ar-**H**), 6.96 (dd, J = 11.9, 1.6 Hz, 1H, Ar-**H**), 2.60 (t, J = 6.8 Hz, 2H, Ar-C**H₂**-CH₂), 1.62 (h, J = 7.3 Hz, 2H, CH₂-C**H₂**-CH₃), 0.89 (t, J = 7.3 Hz, 3H, CH₂-C**H₃**).

$^{13}C\{^{1}H\}$ NMR (101 MHz, $CDCl_3$) (δ): 164.71 (dd, J = 221.0, 5.2 Hz), 162.50 (d, J = 261.2 Hz), 162.12 (d, J = 5.2 Hz), 152.23 (d, J = 8.6 Hz), 152.11, 148.49 ($t_{apparent}$, J = 9.8 Hz), 134.79 ($t_{apparent}$, J = 2.8 Hz), 132.41, 128.33, 124.53, 124.50, 122.85, 124.51 (d, J = 3.3 Hz), 117.06 (d, J = 21.8 Hz), 114.78 (d, J = 9.5 Hz), 110.53 (dd, J = 20.5, 3.3 Hz), 109.30, 90.78 ($t_{apparent}$, J = 19.4 Hz), 37.85, 23.89, 13.67.

^{19}F NMR (376 MHz, $CDCl_3$) (δ): -103.33 (d, J = 9.4 Hz, 2F, Ar-**F**), -108.44 (dd, J = 12.0, 7.7 Hz, 1F, Ar-**F**).

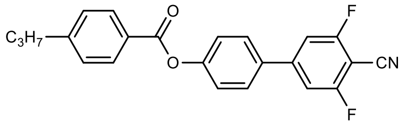

18 (**2.0.0**): *4'-Cyano-3',5' difluoro-[1,1' biphenyl]4-yl-4-propyl benzoate*

Yield: (white solid) 230 mg, 61%

R_F (DCM): 0.87

^{1}H NMR (400 MHz, $CDCl_3$) (δ): 8.05 (ddd, J = 8.3, 1.9, 1.7 Hz, 2H, Ar-**H**), 7.55 (ddd, J = 8.7, 2.7, 2.3 Hz, 2H, Ar-**H**), 7.31 – 7.23 (m, 4H, Ar-**H**)*⁺, 7.19 ($d_{apparent}$, J = 8.5 Hz, 2H, Ar-**H**), 2.62 (t, J = 6.8 Hz, 2H, Ar-C**H₂**-CH₂), 1.63 (h, J = 7.4 Hz, 2H, CH₂-C**H₂**-CH₃), 0.90 (t, J = 7.3 Hz, 3H, CH₂-C**H₃**).

*overlapping peaks, ⁺overlapping with solvent peak

$^{13}C\{^1H\}$ NMR (101 MHz, $CDCl_3$) (δ): 165.00, 163.43 (dd, J = 260.8, 5.4 Hz), 152.48, 149.59, 148.54 ($t_{apparent}$, J = 9.8 Hz), 134.67 ($t_{apparent}$, J = 2.0 Hz), 130.34, 128.86, 128.34, 126.51, 122.90, 110.52 (dd, J = 20.3, 3.4 Hz), 109.31, 90.69 ($t_{apparent}$, J = 19.1 Hz), 38.15, 24.26, 13.76.

^{19}F NMR (376 MHz, $CDCl_3$) (δ): -103.31 ($d_{apparent}$, J = 9.3 Hz, 2F, Ar-**F**).

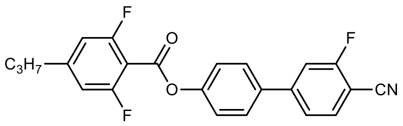

19 (**1.0.2**): *4'-Cyano-3' fluoro-[1,1' biphenyl]4-yl 2,6 difluoro-4-propyl benzoate*

Yield: (shiny white solid) 280 mg, 71%

R_F (DCM): 0.79

^1H NMR (400 MHz, $CDCl_3$) (δ): 7.70 (t, J = 7.8 Hz, 1H, Ar-**H**), 7.64 (ddd, J = 8.6, 2.6, 1.9 Hz, 2H, Ar-**H**), 7.48 (dd, J = 8.1, 1.7 Hz, 1H, Ar-**H**), 7.43 (dd, J = 10.2, 1.3 Hz, 1H, Ar-**H**), 7.39 (ddd, J = 8.7, 2.9, 1.9 Hz, 2H), 6.86 ($d_{apparent}$, J = 9.9 Hz, 2H, Ar-**H**), 2.65 (t, J = 7.6 Hz, 2H, Ar-C**H₂**-CH₂), 1.68 (h, J = 7.4 Hz, 2H, CH_2-C$\mathbf{H_2}$-CH_3), 0.97 (t, J = 7.3 Hz, 3H, CH_2-C$\mathbf{H_3}$).

$^{13}C\{^1H\}$ NMR (101 MHz, $CDCl_3$) (δ): 164.76 (d, J = 259.7 Hz), 162.19 (dd, J = 258.3, 5.8 Hz), 150.83, 150.78 ($t_{apparent}$, J = 10.1 Hz), 147.59 (d, J = 7.9 Hz), 136.07 (d, J = 1.5 Hz), 133.84, 128.45, 123.40 (d, J = 3.3 Hz), 122.58, 114.86 (d, J = 20.3 Hz), 114.00, 112.22 (dd, J = 21.1, 2.7 Hz), 100.06 (d, J = 15.8 Hz), 37.88, 23.66, 13.57.

^{19}F NMR (376 MHz, $CDCl_3$) (δ): -105.91 ($t_{apparent}$, J = 7.4 Hz, 1F, Ar-**F**), -109.64 (d, J = 10.1 Hz, 2F, Ar-**F**).

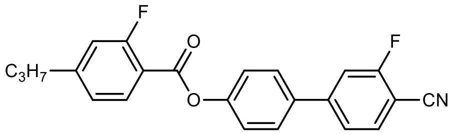

20 (**1.0.1**): *4'-Cyano-3' fluoro-[1,1' biphenyl]4-yl 2 fluoro-4-propyl benzoate*

Yield: (White solid) 155 mg, 41%

R_F (DCM): 0.84

^1H NMR (400 MHz, $CDCl_3$) (δ): 8.02 (t, J = 7.8 Hz, 1H, Ar-**H**), 7.69 (dd, J = 8.1, 6.6 Hz, 1H, Ar-**H**), 7.63 (ddd, J = 8.8, 2.7, 2.1 Hz, 2H, Ar-**H**), 7.48 (dd, J = 8.1, 1.7 Hz, 1H, Ar-**H**), 7.43 (dd, J = 10.1, 1.6 Hz, 1H, Ar-**H**), 7.40 – 7.33 (m, 2H, Ar-**H**), 7.10 (dd, J = 8.0, 1.6 Hz, 1H, Ar-**H**), 7.04 (dd, J = 11.8, 1.6 Hz, 1H, Ar-**H**), 2.68 (t, J = 6.8 Hz, 2H, Ar-C$\mathbf{H_2}$-CH_2), 1.70 (h, J = 7.3 Hz, 2H, CH_2-C$\mathbf{H_2}$-CH_3), 0.98 (t, J = 7.3 Hz, 3H, CH_2-C$\mathbf{H_3}$).

$^{13}C\{^{1}H\}$ NMR (101 MHz, $CDCl_3$) (δ): 164.76 (d, J = 258.9 Hz), 162.63 (d, J = 4.1 Hz), 162.39 (d, J = 261.5 Hz), 152.11 (d, J = 8.7 Hz), 151.65, 147.67 (d, J = 8.1 Hz), 135.77 (d, J = 1.7 Hz), 133.82, 132.41, 128.39, 124.48 (d, J = 3.2 Hz), 123.38 (d, J = 3.2 Hz), 122.68, 114.93 (d, J = 9.2 Hz), 114.79 (d, J = 20.5 Hz), 114.93 (d, J = 9.2 Hz), 114.79 (d, J = 20.5 Hz), 99.97 (d, J = 15.6 Hz), 37.85, 23.89, 13.67.

^{19}F NMR (376 MHz, $CDCl_3$) (δ): -105.97 (dd, J = 10.1, 6.6 Hz, 1F, Ar-**F**), -108.50 (dd, J = 11.8, 7.5 Hz, 1F, Ar-**F**).

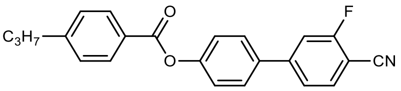

21 (**1.0.0**): *4'-Cyano-3' fluoro-[1,1' biphenyl]4-yl-4-propyl benzoate*

Yield: (white solid) 233 mg, 65%

R_F (DCM): 0.87

^{1}H NMR (400 MHz, $CDCl_3$) (δ): 8.13 (ddd, J = 8.4, 1.8, 1.6 Hz, 2H, Ar-**H**), 7.69 (t, J = 8.1 Hz, 1H, Ar-**H**), 7.63 (ddd, J = 8.8, 2.6, 2.3 Hz, 2H, Ar-**H**), 7.48 (dd, J = 8.1, 1.7 Hz, 1H, Ar-**H**), 7.43 (dd, J = 10.1, 1.6 Hz, 1H, Ar-**H**), 7.37 – 7.31 (m, 4H, Ar-**H**)*, 2.70 (t, J = 7.6 Hz, 2H, Ar-C**H₂**-CH₂), 1.71 (h, J = 7.5 Hz, 2H, CH_2-C**H₂**-CH_3), 0.98 (t, J = 7.3 Hz, 3H, CH_2-C**H₃**).

*overlapping peaks

$^{13}C\{^{1}H\}$ NMR (101 MHz, $CDCl_3$) (δ): 165.08, 163.48 (d, J = 258.7 Hz), 152.02, 149.51, 147.70 (d, J = 8.1 Hz), 135.60 (d, J = 1.9 Hz), 133.82, 130.33, 128.85, 128.39, 123.37 (d, J = 3.2 Hz), 122.73, 114.78 (d, J = 20.3 Hz), 114.05, 100.00, 99.92 (d, J = 15.6 Hz), 38.15, 24.27, 13.77.

^{19}F NMR (376 MHz, $CDCl_3$) (δ): -105.99 ($t_{apparent}$, J = 7.6 Hz, 1F, Ar-**F**).

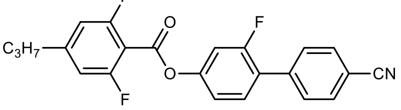

22 (**0.1.2**): *4'-Cyano-2 fluoro-[1,1' biphenyl]4-yl 2,6 difluoro-4-propyl benzoate*

Yield: (shiny white solid) 256 mg, 65%

R_F (DCM): 0.83

^{1}H NMR (400 MHz, $CDCl_3$) (δ): 7.74 (ddd, J = 8.6, 1.9, 1.6 Hz, 2H, Ar-**H**), 7.68 – 7.63 ($m_{apparent}$, 2H, Ar-**H**), 7.48 (t, J = 8.6 Hz, 1H, Ar-**H**), 7.22 – 7.14 (m, 2H, Ar-**H**)*, 6.87 ($d_{apparent}$, J = 9.4 Hz,

2H, Ar-**H**), 2.65 (t, J = 7.3 Hz, 2H, Ar-C**H**$_2$-CH$_2$), 1.68 (h, J = 7.5 Hz, 2H, CH$_2$-C**H**$_2$-CH$_3$), 0.97 (t, J = 7.3 Hz, 3H, CH$_2$-C**H**$_3$).

*overlapping peaks

^{13}C{^{1}H} NMR (101 MHz, CDCl$_3$) (δ): 162.48 (dd, J = 258.1, 6.0 Hz), 159.58, 159.55 (d, J = 251.1 Hz), 151.56 – 150.89 (m), 139.69, 132.34, 130.90 (d, J = 4.1 Hz), 129.67 (d, J = 3.2 Hz), 125.24 (d, J = 13.1 Hz), 118.71, 118.24 (d, J = 3.7 Hz), 112.30 (dd, J = 21.6, 3.1 Hz), 111.63, 110.68 (d, J = 26.0 Hz), 106.76 ($t_{apparent}$, J = 16.5 Hz), 37.89, 23.64, 13.57.

^{19}F NMR (376 MHz, CDCl$_3$) (δ): -109.42 (d, J = 10.2 Hz, 2F, Ar-**F**), -114.23 ($t_{apparent}$, J = 9.8 Hz, 1F, Ar-**F**).

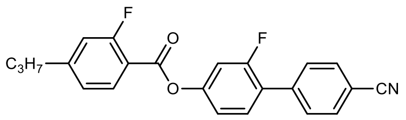

23 (**0.1.1**): *4'-Cyano-2 fluoro-[1,1' biphenyl]4-yl 2 fluoro-4-propyl benzoate*

Yield: (white solid) 203 mg, 54%

R_F (DCM): 0.84

^{1}H NMR (400 MHz, CDCl$_3$) (δ): 8.01 (t, J = 7.8 Hz, 1H, Ar-**H**), 7.75 (ddd, J = 8.6, 1.8, 1.3 Hz, 2H, Ar-**H**), 7.69 – 7.63 ($m_{apparent}$, 2H, Ar-**H**), 7.48 (t, J = 8.6 Hz, 1H, Ar-**H**), 7.19 – 7.13 (m, 2H, Ar-**H**)*, 7.10 (dd, J = 8.1, 1.6 Hz, 1H, Ar-**H**), 7.04 (dd, J = 11.9, 1.6 Hz, 1H, Ar-**H**), 2.68 (t, J = 6.8 Hz, 2H, Ar-C**H**$_2$-CH$_2$), 1.69 (h, J = 7.3 Hz, 2H, CH$_2$-C**H**$_2$-CH$_3$), 0.97 (t, J = 7.4 Hz, 3H, CH$_2$-C**H**$_3$).

*overlapping peaks

^{13}C{^{1}H} NMR (101 MHz, CDCl$_3$) (δ): 162.44 (d, J = 261.5 Hz), 162.29, 161.21, 159.56 (d, J = 251.3 Hz), 152.32 (d, J = 8.7 Hz), 151.67 (d, J = 11.0 Hz), 139.78, 132.42, 132.33, 130.83 (d, J = 4.1 Hz), 129.66 (d, J = 3.2 Hz), 124.95 (d, J = 13.1 Hz), 124.53 (d, J = 3.2 Hz), 118.74, 118.35 (d, J = 3.7 Hz), 117.09 (d, J = 21.7 Hz), 114.62 (d, J = 9.5 Hz), 111.57, 110.77 (d, J = 25.9 Hz), 37.86, 23.88, 13.66.

^{19}F NMR (376 MHz, CDCl$_3$) (δ): -108.29 (dd, J = 12.0, 7.7 Hz, 1F, Ar-**F**), -114.45 ($t_{apparent}$, J = 9.7 Hz, 1F, Ar-**F**).

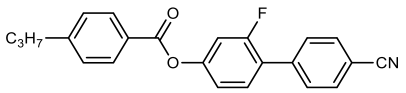

24 (**0.1.0**): *4'-Cyano-2 fluoro-[1,1' biphenyl]4-yl-4-propyl benzoate*

Yield: (shiny white solid) 220 mg, 61%

R_F (DCM): 0.87

^{1}H NMR (400 MHz, $CDCl_3$) (δ): 8.12 (ddd, J = 8.3, 1.8, 1.8 Hz, 2H, Ar-**H**), 7.74 (ddd, J = 8.5, 1.9, 1.8 Hz, 2H, Ar-**H**), 7.69 – 7.64 (m, 2H, Ar-**H**)*, 7.47 (t, J = 8.3 Hz, 1H, Ar-**H**), 7.34 (ddd, J = 8.3, 1.9, 1.9 Hz, 2H, Ar-**H**), 7.18 – 7.10 ($m_{apparent}$, 1H, Ar-**H**), 2.70 (t, J = 7.5 Hz, 2H, Ar-C$\mathbf{H_2}$-CH_2), 1.70 (h, J = 7.2 Hz, 2H, CH_2-C$\mathbf{H_2}$-CH_3), 0.98 (t, J = 7.3 Hz, 3H, CH_2-C$\mathbf{H_3}$).

*overlapping peaks

^{13}C{^{1}H} NMR (101 MHz, $CDCl_3$) (δ): 164.77, 159.58 (d, J = 251.1 Hz), 152.08 (d, J = 11.1 Hz), 149.69, 139.82, 132.33, 130.82 (d, J = 4.2 Hz), 130.37, 129.66 (d, J = 3.3 Hz), 128.89, 124.79 (d, J = 13.2 Hz), 124.85, 124.72, 118.75, 118.40 (d, J = 3.7 Hz), 111.54, 110.80 (d, J = 25.7 Hz), 38.15, 24.25, 13.77.

^{19}F NMR (376 MHz, $CDCl_3$) (δ): -114.50 ($t_{apparent}$, *J* = 9.8 Hz, 1F, Ar-**F**).

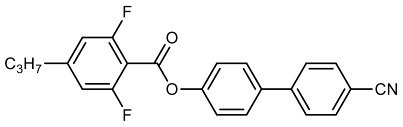

25 (**0.0.2**): *4'-Cyano-[1,1' biphenyl]4-yl 2,6 difluoro-4-propyl benzoate*

Yield: (white solid) 271 mg, 72%

R_F (DCM): 0.80

^{1}H NMR (400 MHz, $CDCl_3$) (δ): 7.76 – 7.66 ($m_{apparent}$, 2H, Ar-**H**), 7.64 (ddd, J = 8.7, 2.8, 2.1 Hz, 2H, Ar-**H**), 7.37 (ddd, J = 8.6, 2.7, 2.1 Hz, 2H, Ar-**H**), 6.86 ($d_{apparent}$, J = 9.3 Hz, 2H, Ar-**H**), 2.64 (t, J = 7.6 Hz, 2H, Ar-C$\mathbf{H_2}$-CH_2), 1.68 (h, J = 7.6 Hz, 2H, CH_2-C$\mathbf{H_2}$-CH_3), 0.97 (t, J = 7.4 Hz, 3H, CH_2-C$\mathbf{H_3}$).

^{13}C{^{1}H} NMR (101 MHz, $CDCl_3$) (δ): 160.91 (dd, J = 257.5, 6.9 Hz), 160.01, 150.88, 150.73 ($t_{apparent}$, J = 10.4 Hz), 144.73, 137.26, 132.68, 128.45, 127.75, 122.39, 118.86, 112.23 (dd, J = 21.8, 3.1 Hz), 111.14, 107.20 ($t_{apparent}$, J = 16.9 Hz)., 37.87, 23.66, 13.57.

^{19}F NMR (376 MHz, $CDCl_3$) (δ): -109.71 ($d_{apparent}$, J = 10.0 Hz, 2F, Ar-**F**).

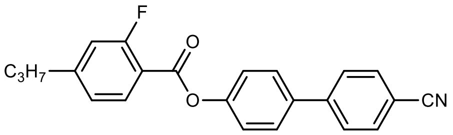

26 (**0.0.1**): *4'-Cyano-[1,1' biphenyl]4-yl 2 fluoro-4-propyl benzoate*

Yield: (shiny white solid) 248 mg, 69%

R_F (DCM): 0.80

^{1}H NMR (400 MHz, $CDCl_3$) (δ): 8.02 (t, J = 7.8 Hz, 1H, Ar-**H**), 7.77 – 7.66 (m, 4H, Ar-**H**)*, 7.64 (ddd, J = 8.8, 2.8, 2.1 Hz, 2H, Ar-**H**), 7.35 (ddd, J = 8.7, 2.7, 2.1 Hz, 2H, Ar-**H**), 7.10 (dd, J = 8.1, 1.6 Hz, 1H, Ar-**H**), 7.04 (dd, J = 11.8, 1.6 Hz, 1H, Ar-**H**), 2.68 (t, J = 6.8 Hz, 2H, Ar-C$\mathbf{H_2}$-CH_2), 1.70 (h, J = 7.5 Hz, 2H, CH_2-C$\mathbf{H_2}$-CH_3), 0.98 (t, J = 7.3 Hz, 3H, CH_2-C$\mathbf{H_3}$).

*overlapping peaks

$^{13}C\{^1H\}$ NMR (101 MHz, $CDCl_3$) (δ): 162.71 (d, J = 4.4 Hz), 162.48 (d, J = 261.4 Hz), 152.01 (d, J = 8.7 Hz), 151.19, 144.82, 136.97, 132.68, 132.41, 128.39, 127.73, 124.46 (d, J = 3.3 Hz), 122.50, 118.89, 117.14 (d, J = 22.1 Hz), 115.01 (d, J = 9.4 Hz), 111.07, 37.85, 23.90, 13.67.

^{19}F NMR (376 MHz, $CDCl_3$) (δ): -108.55 (dd, J = 11.8, 7.5 Hz, 1F, Ar-**F**).

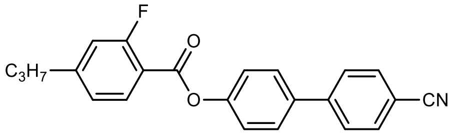

27 (**0.0.0**) *4’-Cyano-[1,1’ biphenyl]4-yl-4-propyl benzoate*

Yield: (shiny white solid) 255 mg, 75%

R_F (DCM): 0.85

^{1}H NMR (400 MHz, $CDCl_3$) (δ): 8.13 (ddd, J = 8.3, 1.7, 1.7 Hz, 2H, Ar-**H**), 7.79 – 7.66 (m, 4H, Ar-**H**)*, 7.64 (ddd, J = 8.5, 2.8, 2.8 Hz, 2H, Ar-**H**), 7.38 – 7.29 (m, 4H, Ar-**H**)*, 2.69 (t, J = 7.7 Hz, 2H, Ar-C$\mathbf{H_2}$-CH_2), 1.70 (h, J = 7.2 Hz, 2H, CH_2-C$\mathbf{H_2}$-CH_3), 0.97 (t, J = 7.3 Hz, 3H, CH_2-C$\mathbf{H_3}$).

*overlapping peaks

$^{13}C\{^1H\}$ NMR (101 MHz, $CDCl_3$) (δ): 165.19, 151.54, 149.42, 144.88, 136.83, 132.68, 130.31, 128.82, 128.40, 127.72, 126.72, 122.54, 118.89, 111.03, 38.14, 24.27, 13.76.

3.4 Example Structural Characterisation

Below are example ^{1}H, $^{13}C\{^1H\}$, and ^{19}F NMR spectra. Full analysed and raw data for all **1-27** is openly available from the University of Leeds Data Repository at https://doi.org/10.5518/1571.

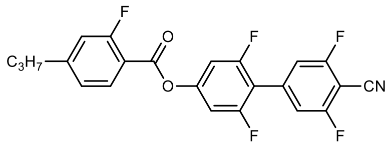
F
O
C3H7
O
F
F
F
F
CN

2 (2.2.1)

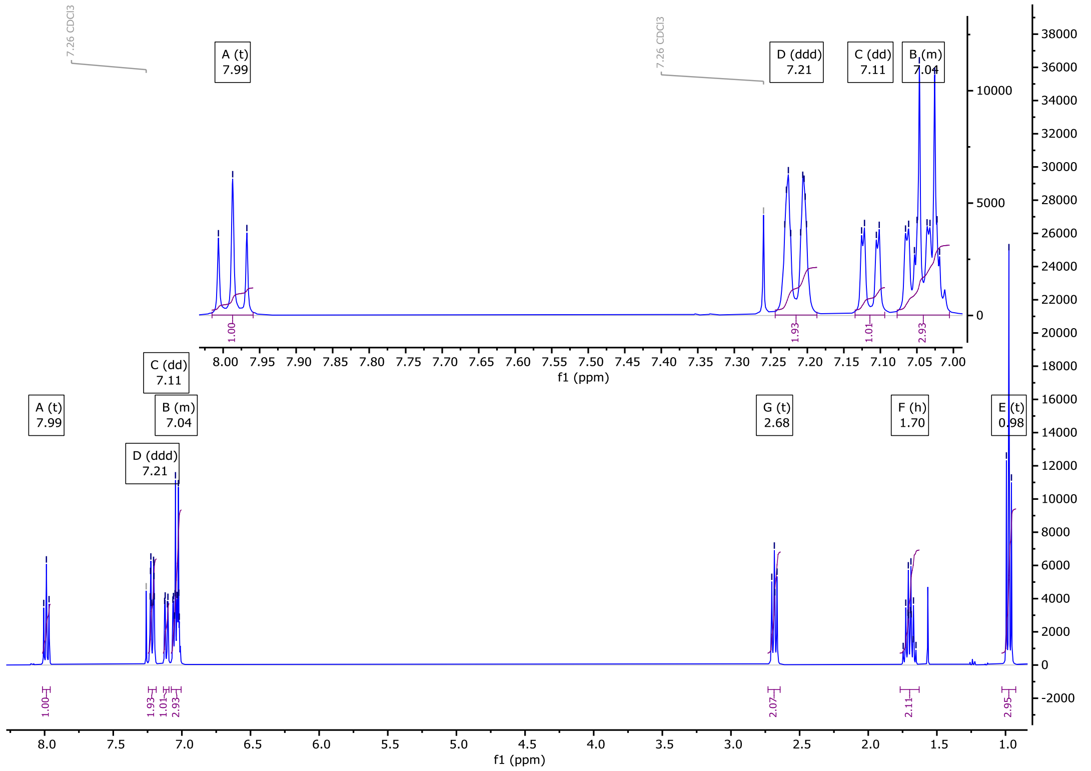
7.26 CDCl3
A (t)
7.99
D (ddd)
7.21
C (dd)
7.11
B (m)
7.04
G (t)
2.68
F (h)
1.70
E (t)
0.98
f1 (ppm)

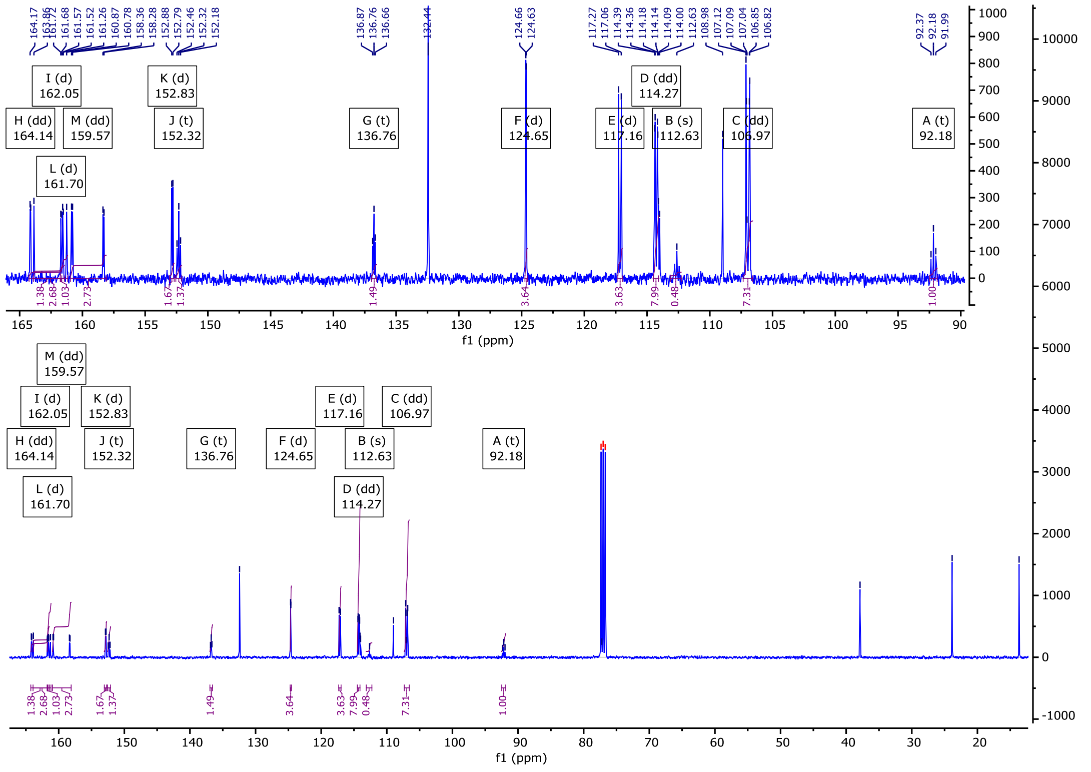
H (dd)
164.14
I (d)
162.05
L (d)
161.70
M (dd)
159.57
K (d)
152.83
J (t)
152.32
G (t)
136.76
F (d)
124.65
E (d)
117.16
D (dd)
114.27
B (s)
112.63
C (dd)
106.97
A (t)
92.18
f1 (ppm)

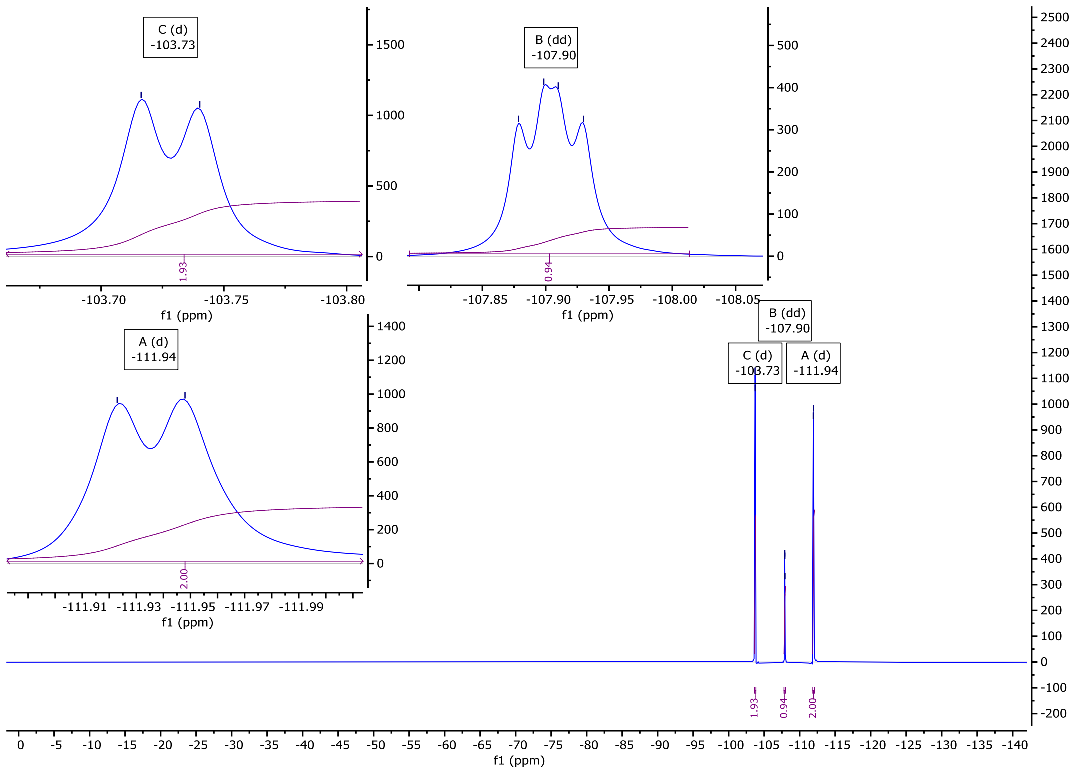

Figure S6: Chemical structure, NMR spectra (^{1}H (top), $^{13}C[1H]$ (middle), and ^{19}F (bottom)) spectra for **2 (2.2.1)**.

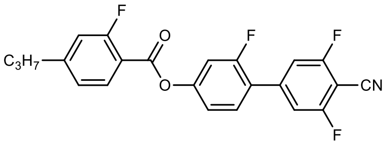
F
O
C3H7
O
F
F
CN
F

5 (2.1.1)

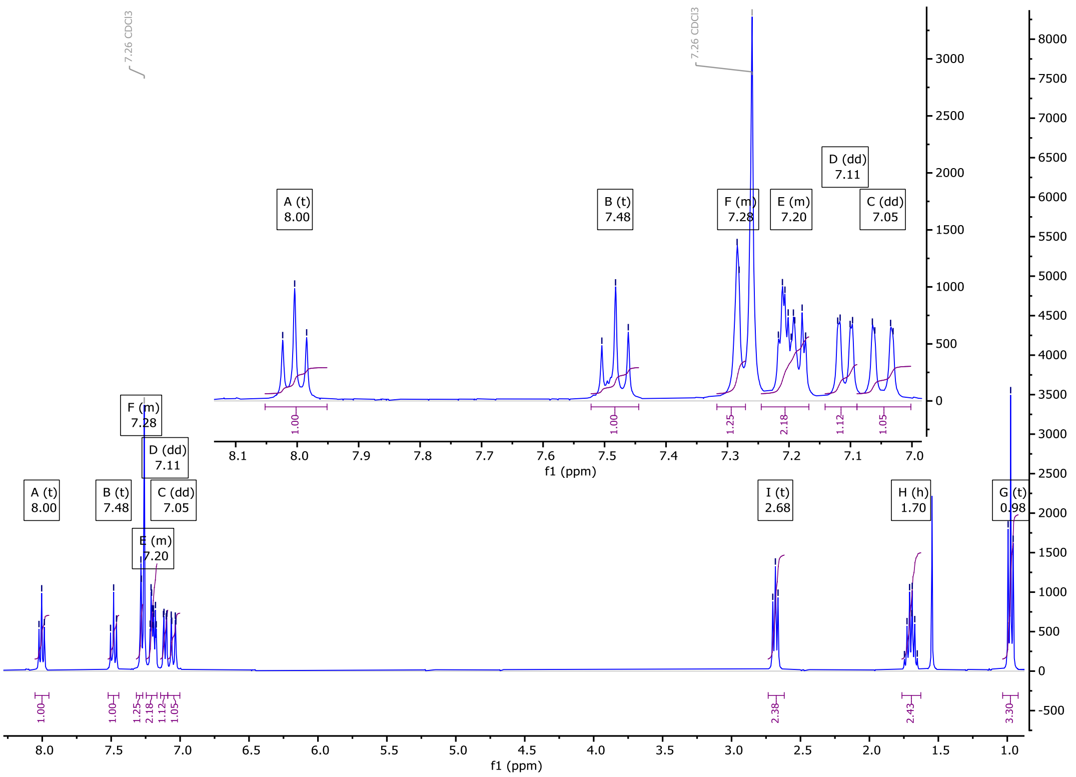
A (t) 8.00
B (t) 7.48
F (m) 7.28
E (m) 7.20
D (dd) 7.11
C (dd) 7.05
I (t) 2.68
H (h) 1.70
G (t) 0.98
7.26 CDCl3
f1 (ppm)

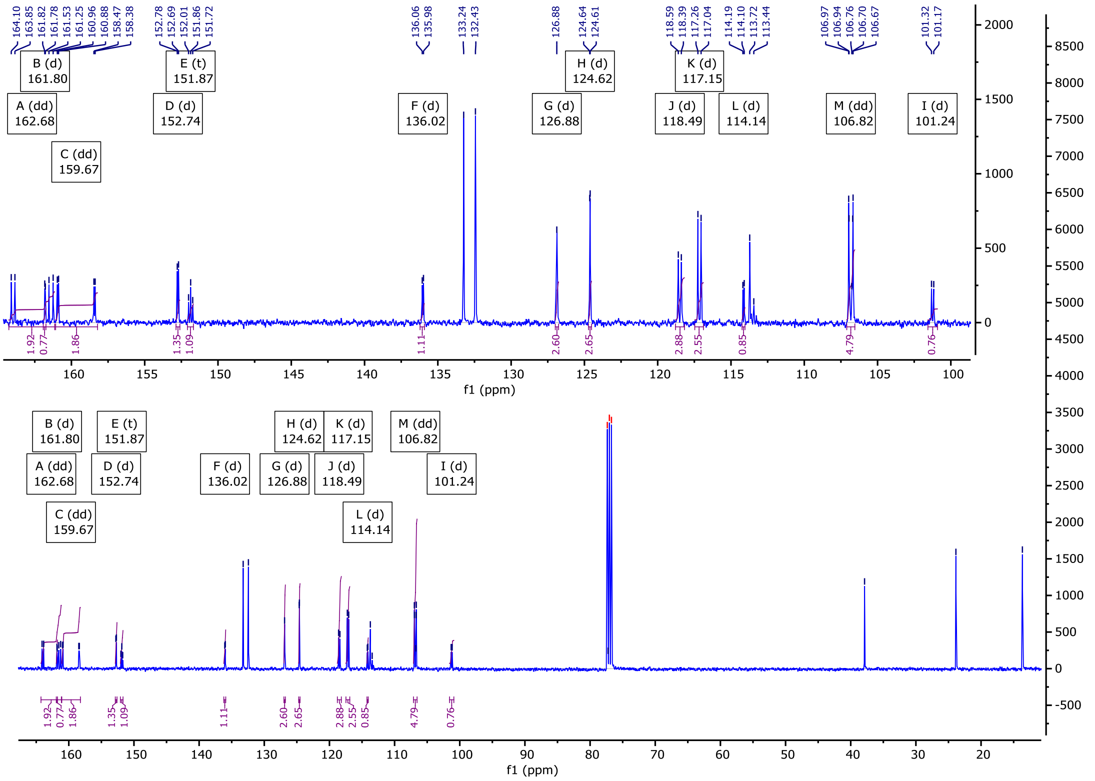
A (dd) 162.68
B (d) 161.80
C (dd) 159.67
D (d) 152.74
E (t) 151.87
F (d) 136.02
G (d) 126.88
H (d) 124.62
J (d) 118.49
K (d) 117.15
L (d) 114.14
M (dd) 106.82
I (d) 101.24
f1 (ppm)

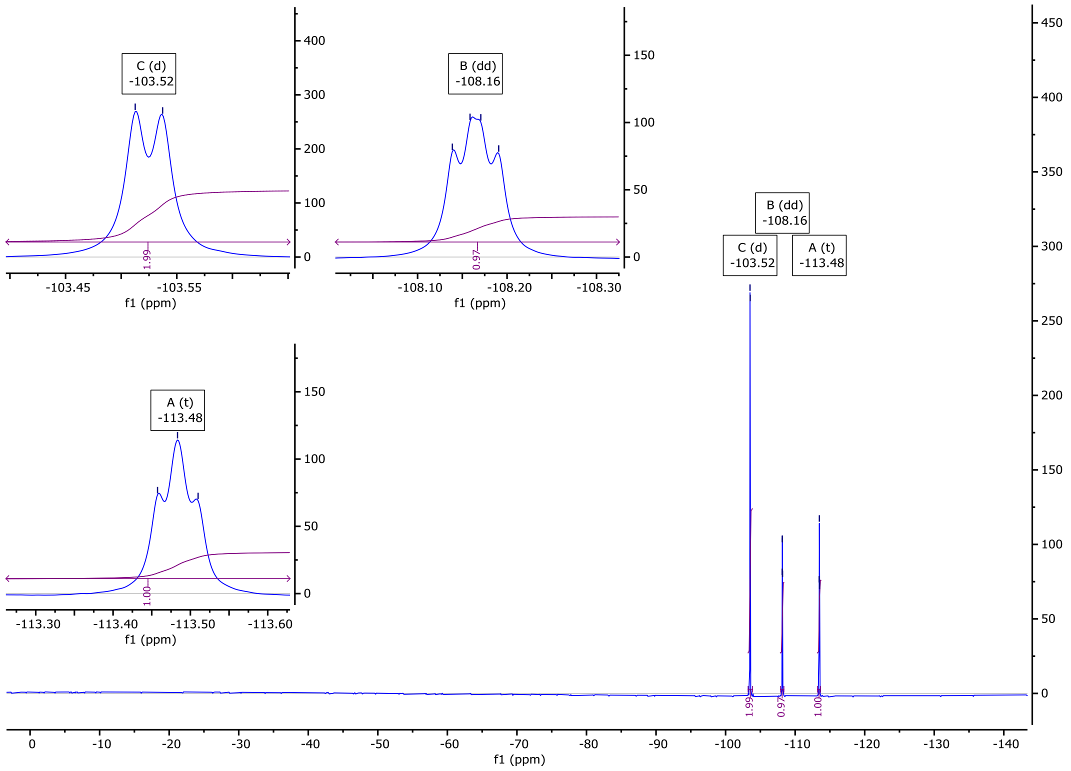

Figure S7: Chemical structure, NMR spectra (^{1}H (top), $^{13}C[1H]$ (middle), and ^{19}F (bottom)) spectra for **5 (2.1.1)**.

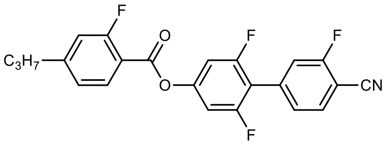
F
O
O
F
F
F
CN
C_3H_7

8 (1.2.1)

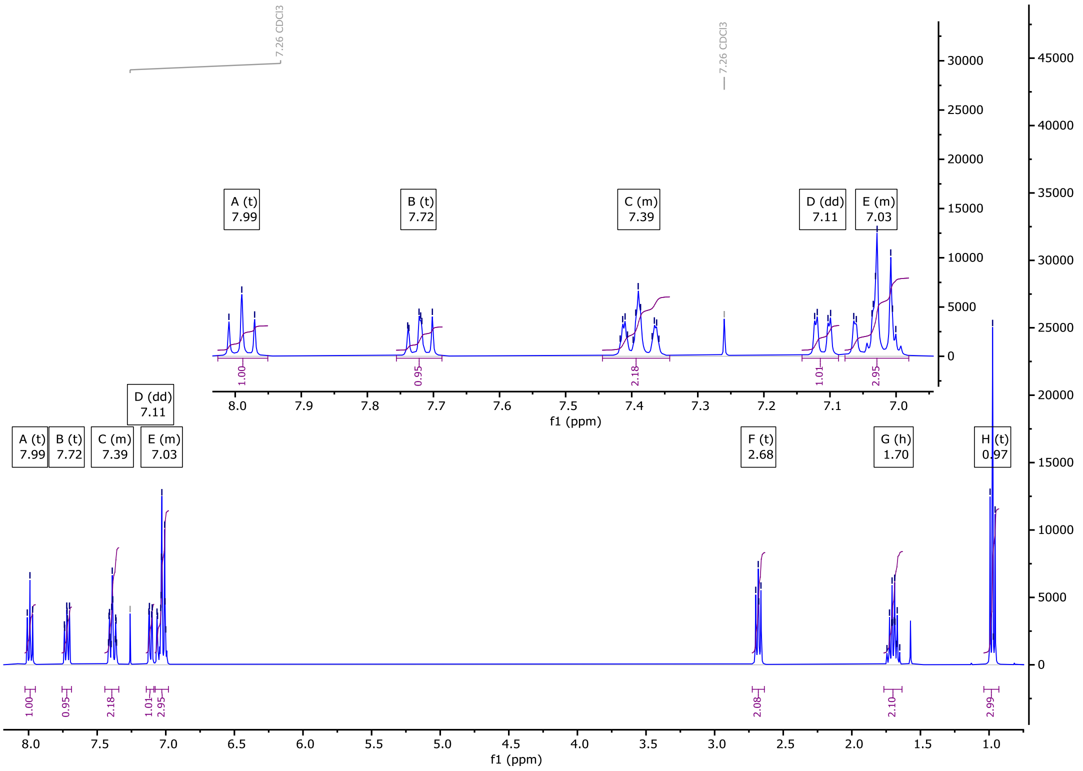
7.26 CDCl3
A (t) 7.99
B (t) 7.72
C (m) 7.39
D (dd) 7.11
E (m) 7.03
F (t) 2.68
G (h) 1.70
H (t) 0.97
f1 (ppm)

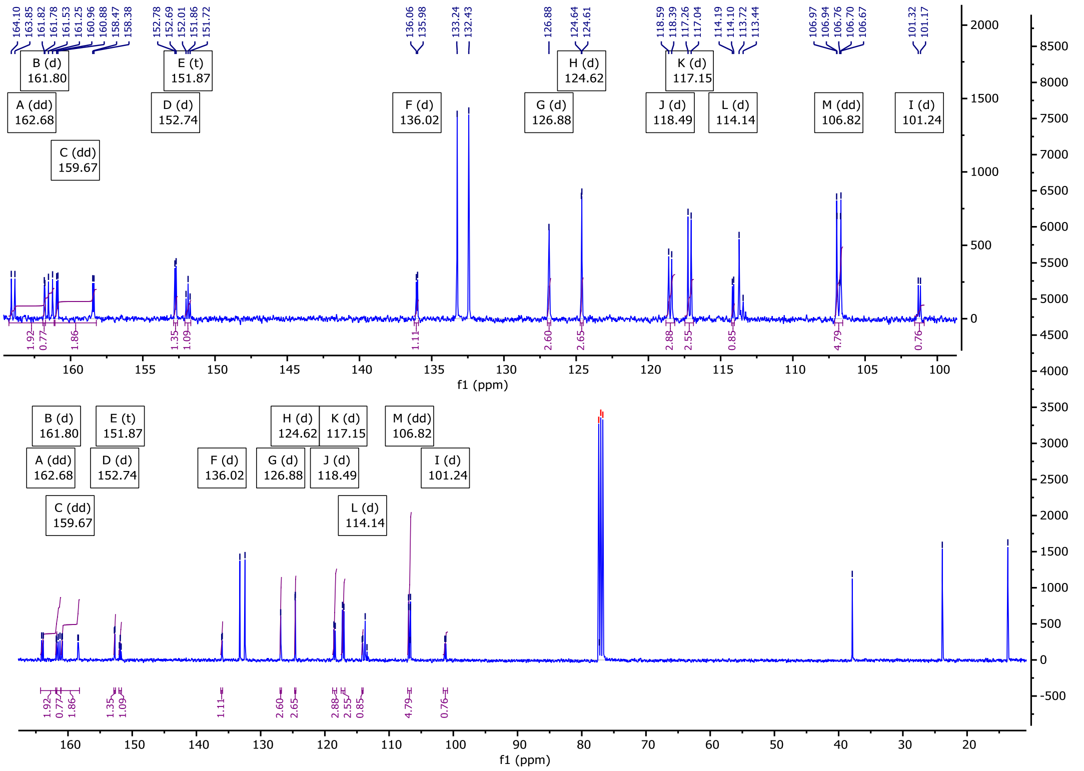
A (dd) 162.68
B (d) 161.80
C (dd) 159.67
D (d) 152.74
E (t) 151.87
F (d) 136.02
G (d) 126.88
H (d) 124.62
J (d) 118.49
K (d) 117.15
L (d) 114.14
M (dd) 106.82
I (d) 101.24
f1 (ppm)

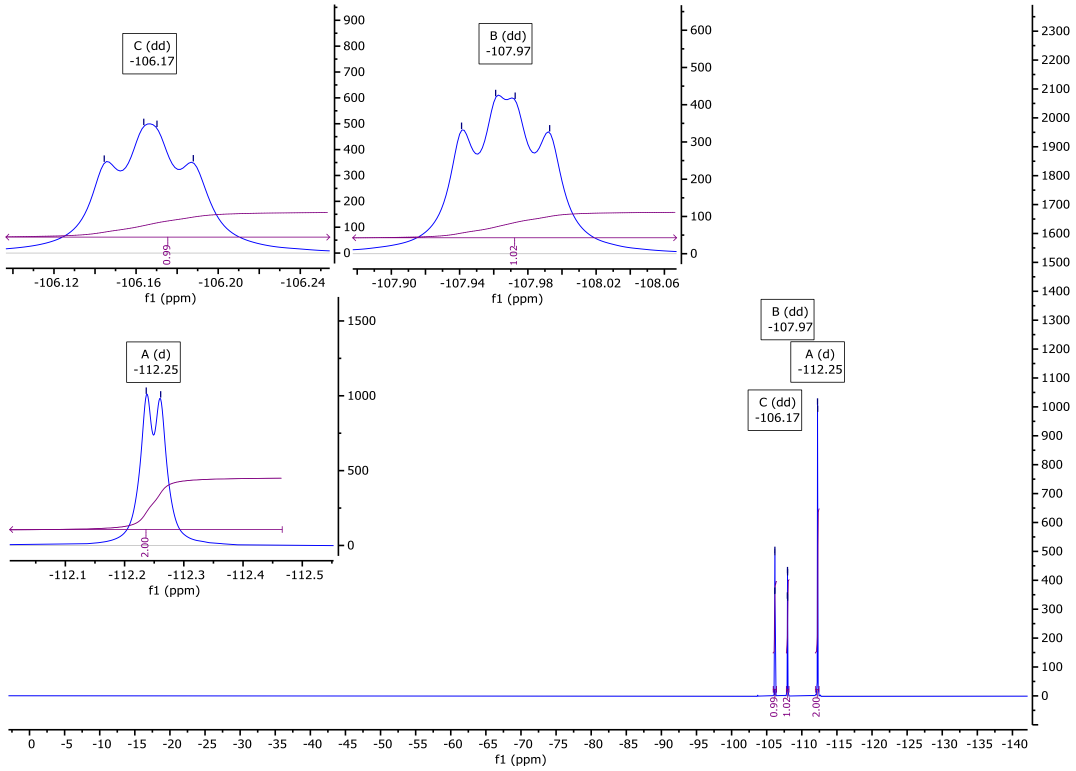

Figure S8: Chemical structure, NMR spectra (^{1}H (top), $^{13}C[1H]$ (middle), and ^{19}F (bottom)) spectra for **8 (1.2.1)**.

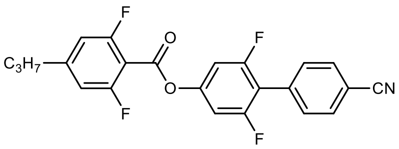
F
O
F
C_3H_7
O
CN
F
F

11 (**2.1.1**)

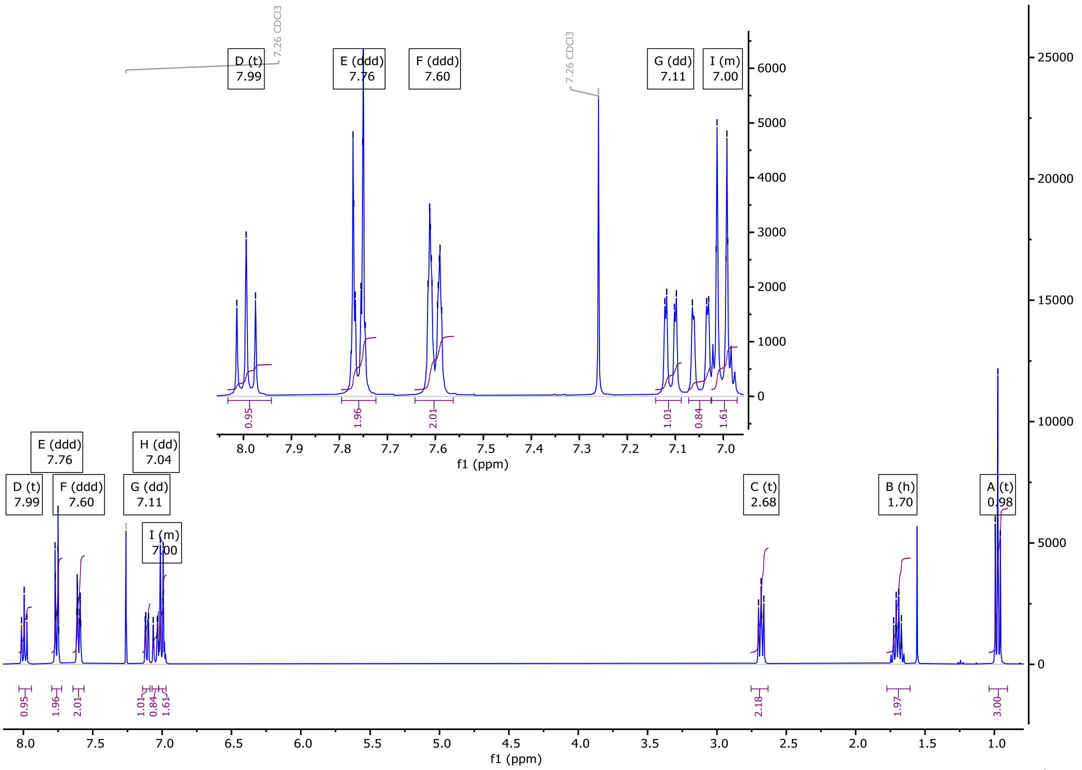
D (t) 7.99
E (ddd) 7.76
F (ddd) 7.60
G (dd) 7.11
H (dd) 7.04
I (m) 7.00
C (t) 2.68
B (h) 1.70
A (t) 0.98
7.26 CDCl3
f1 (ppm)

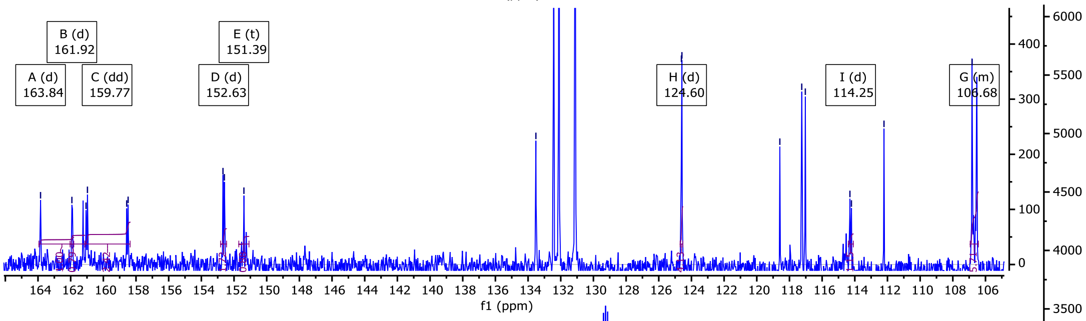
A (d) 163.84
B (d) 161.92
C (dd) 159.77
D (d) 152.63
E (t) 151.39
H (d) 124.60
I (d) 114.25
G (m) 106.68
f1 (ppm)

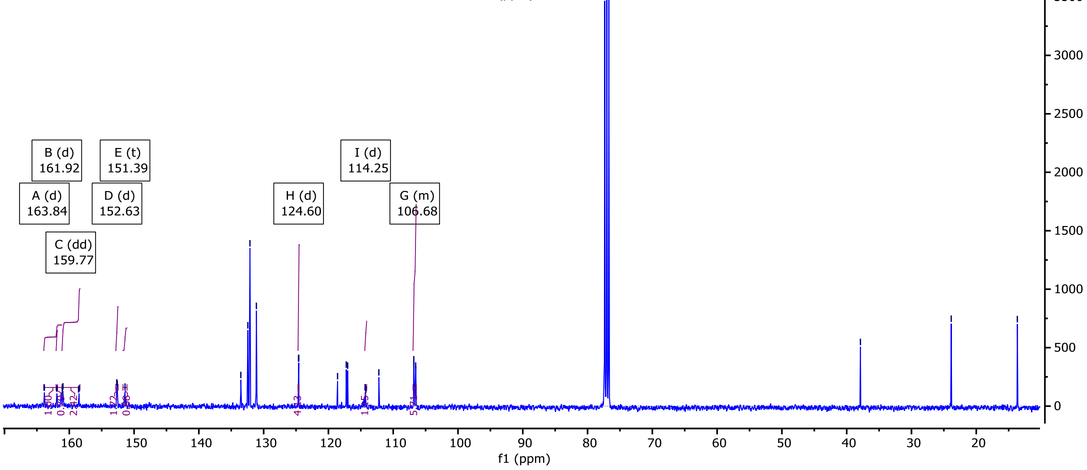
A (d) 163.84
B (d) 161.92
C (dd) 159.77
D (d) 152.63
E (t) 151.39
H (d) 124.60
I (d) 114.25
G (m) 106.68
f1 (ppm)

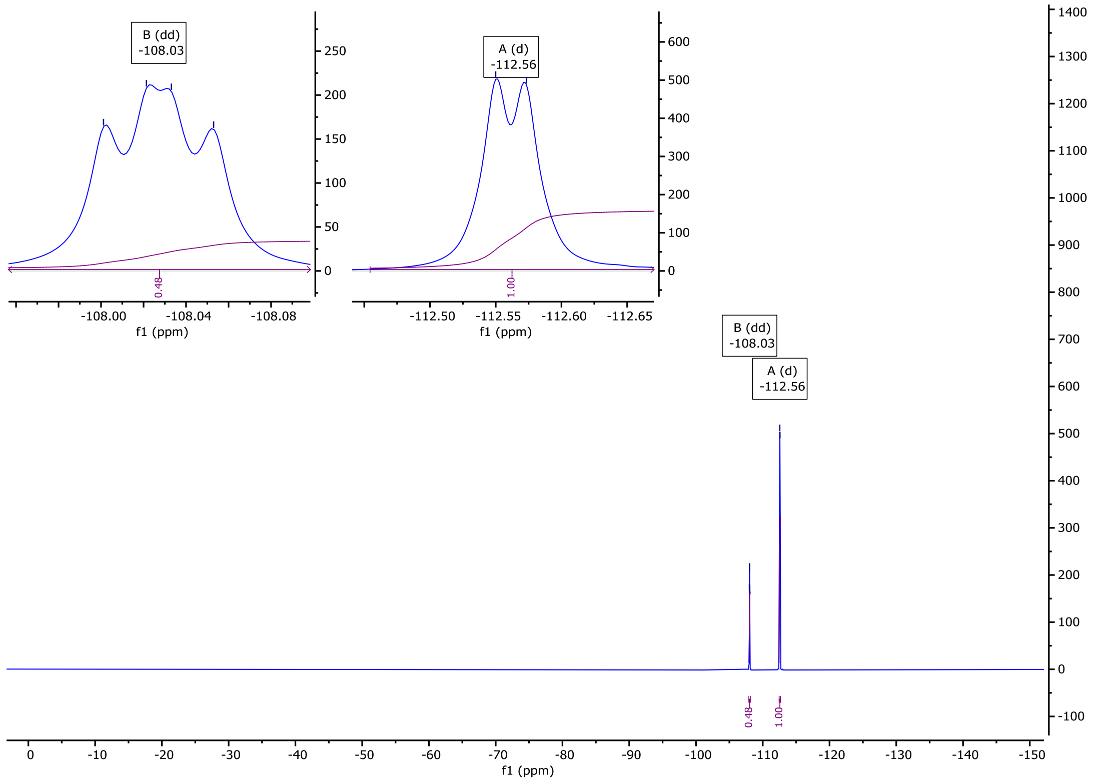

Figure S9: Chemical structure, NMR spectra (^{1}H (top), ^{13}C[1H] (middle), and ^{19}F (bottom)) spectra for **11 (0.2.1)**.

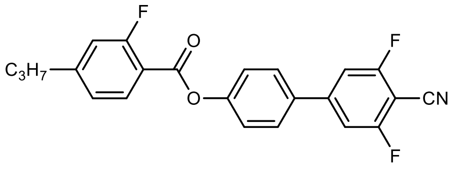

17 (**2.0.1**)

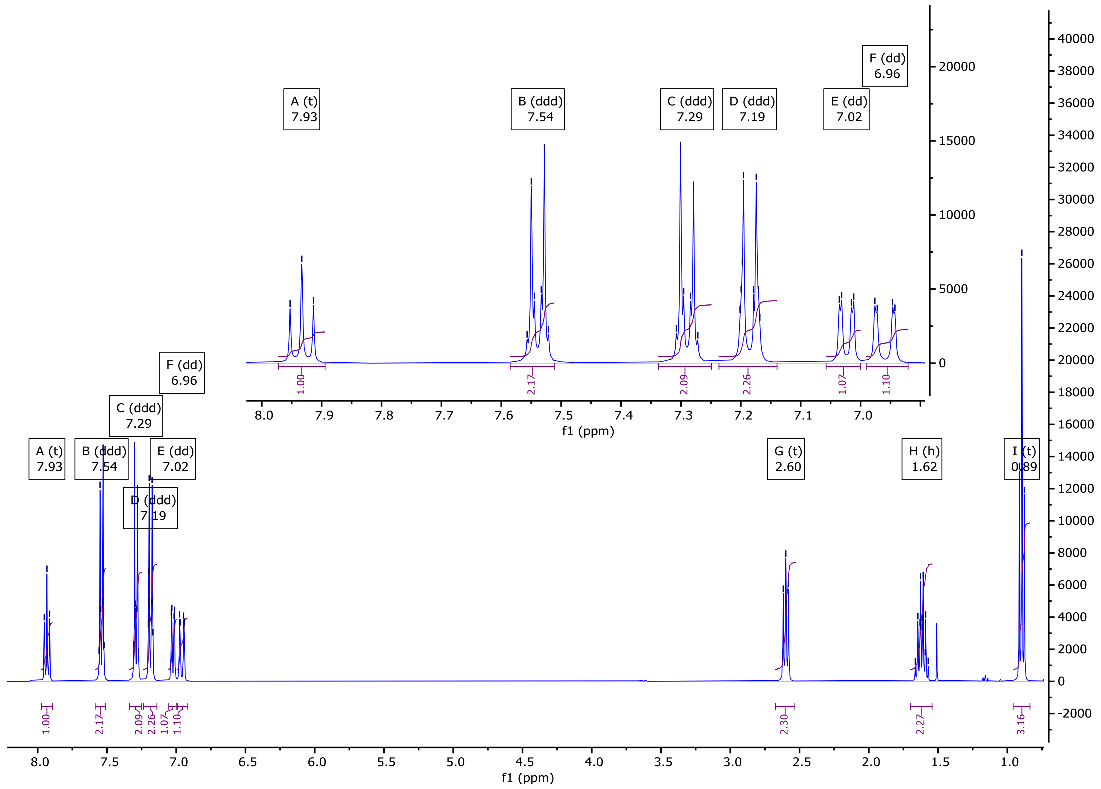

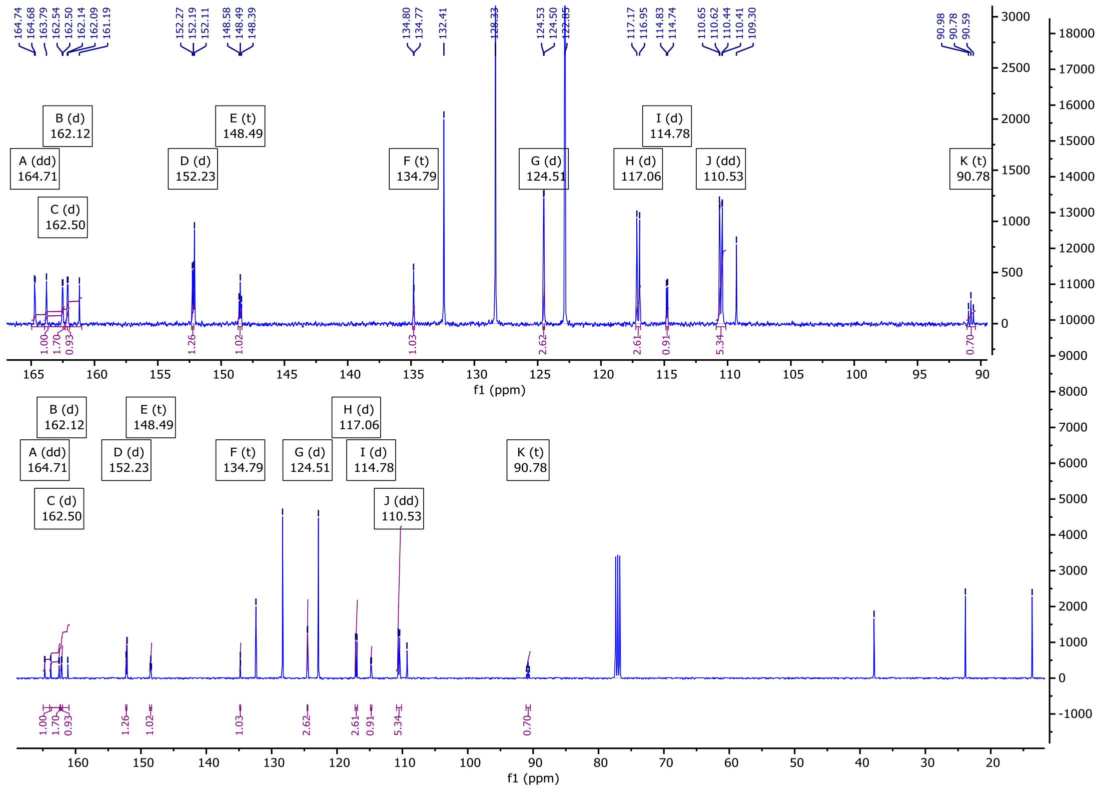

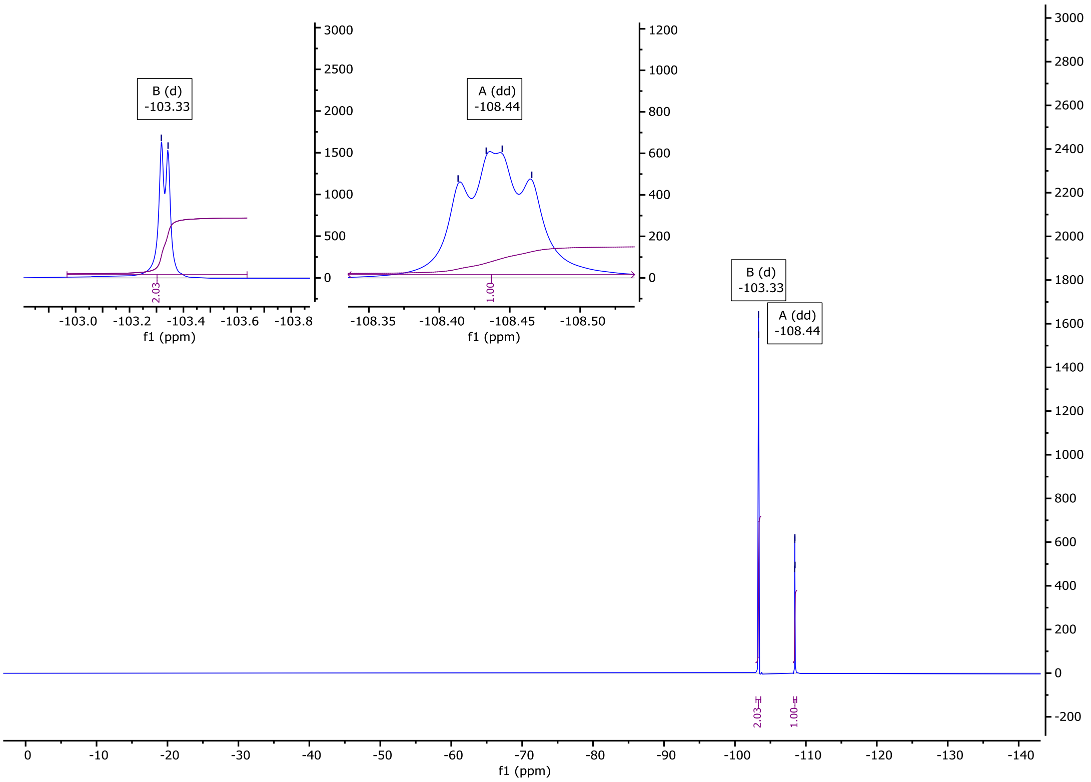

Figure S10: Chemical structure, NMR spectra (^{1}H (top), $^{13}C[1H]$ (middle), and ^{19}F (bottom)) spectra for **17 (2.0.1)**.

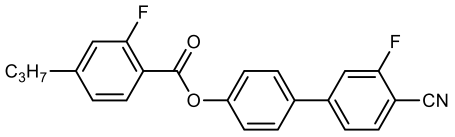

20 (1.0.1)

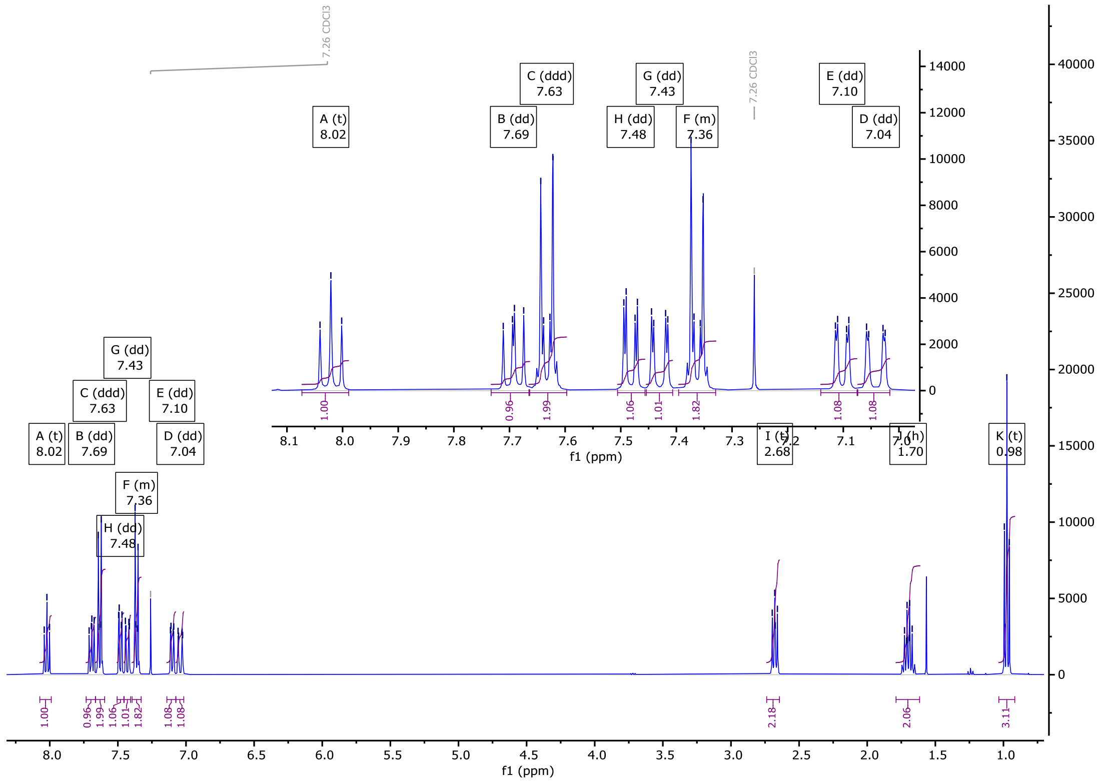

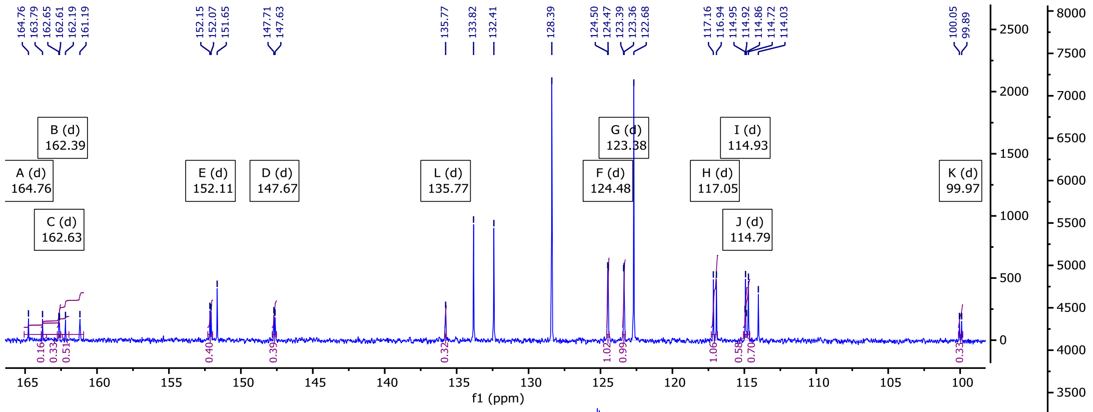

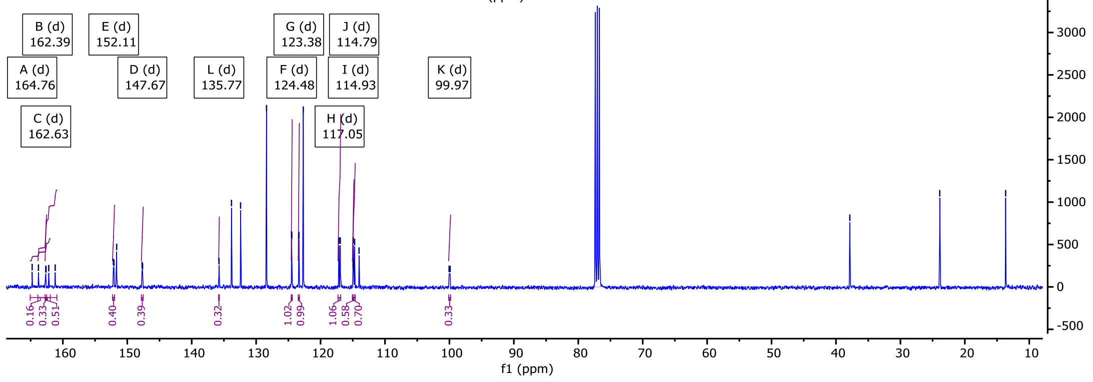

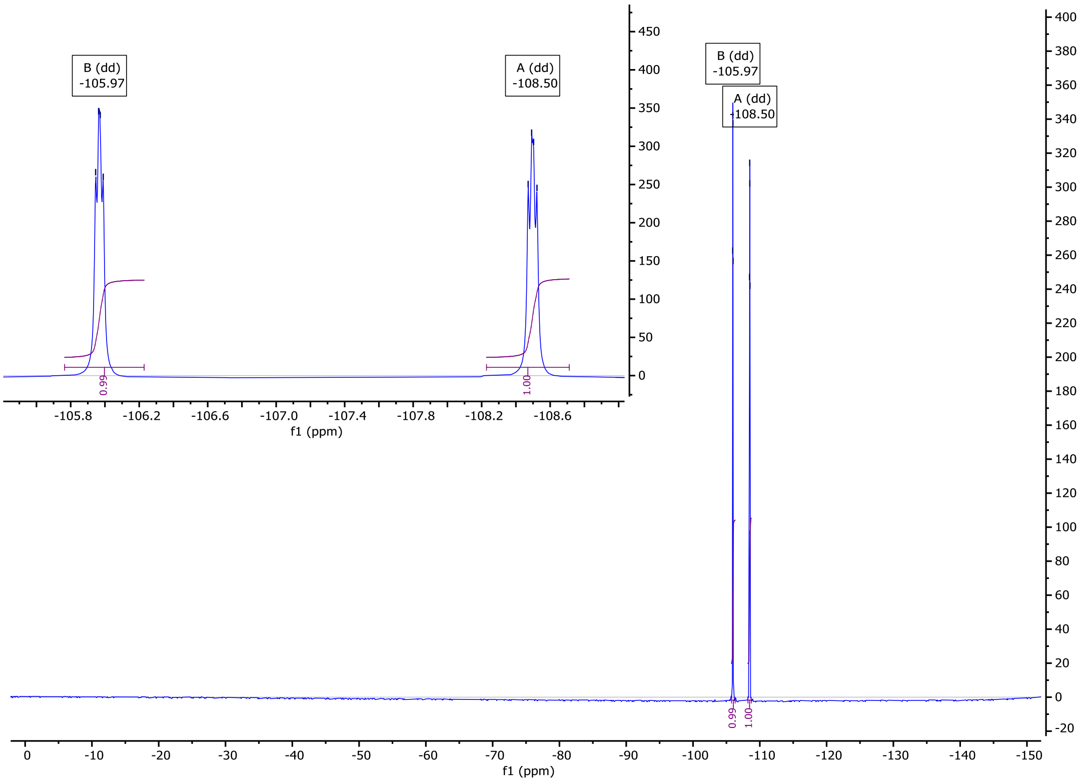

Figure S11: Chemical structure, NMR spectra (^{1}H (top), $^{13}C[1H]$ (middle), and ^{19}F (bottom)) spectra for **20 (1.0.1)**.

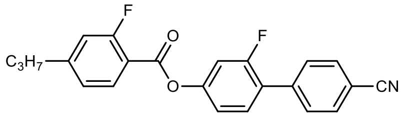
F
O
F
C3H7
O
CN

23 (0.1.1)

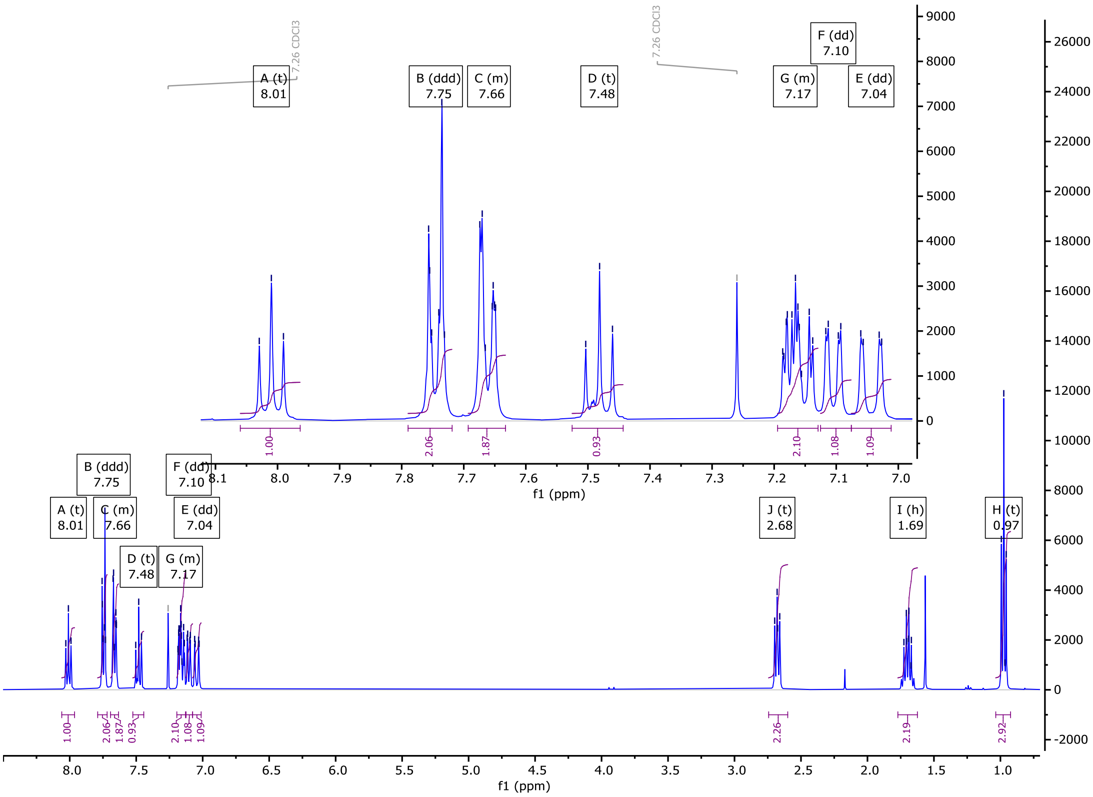
A (t) 8.01
B (ddd) 7.75
C (m) 7.66
D (t) 7.48
E (dd) 7.04
F (dd) 7.10
G (m) 7.17
H (t) 0.97
I (h) 1.69
J (t) 2.68
7.26 CDCl3
f1 (ppm)

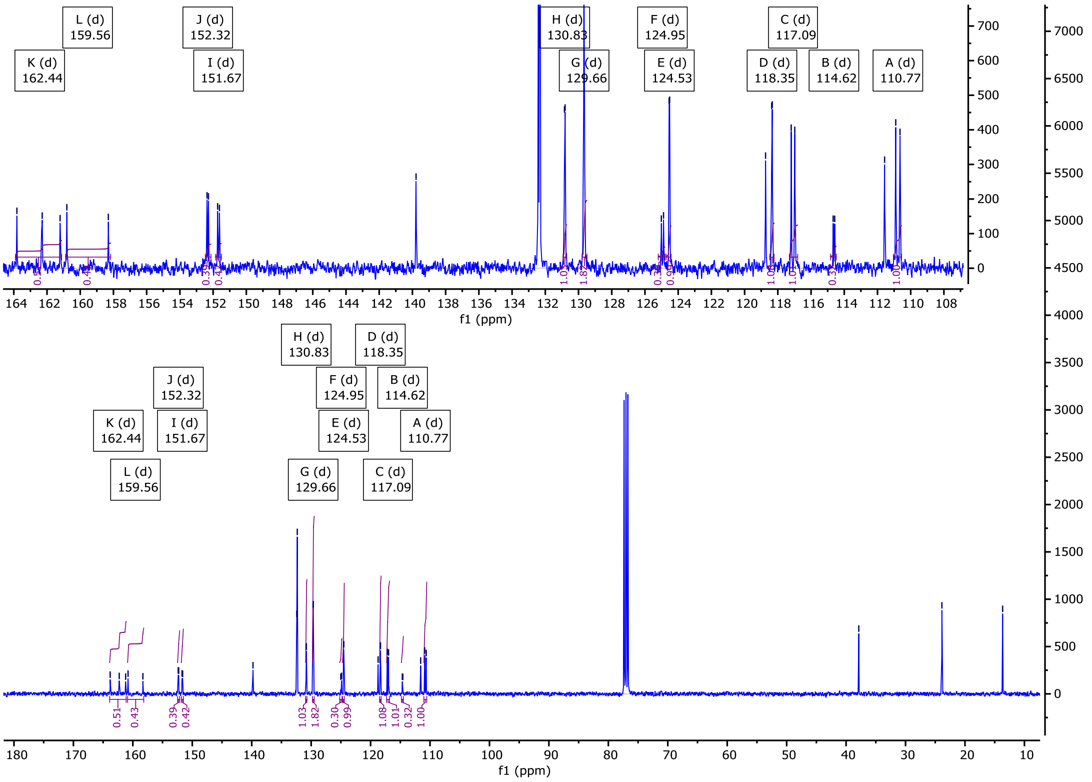
A (d) 110.77
B (d) 114.62
C (d) 117.09
D (d) 118.35
E (d) 124.53
F (d) 124.95
G (d) 129.66
H (d) 130.83
I (d) 151.67
J (d) 152.32
K (d) 162.44
L (d) 159.56
f1 (ppm)

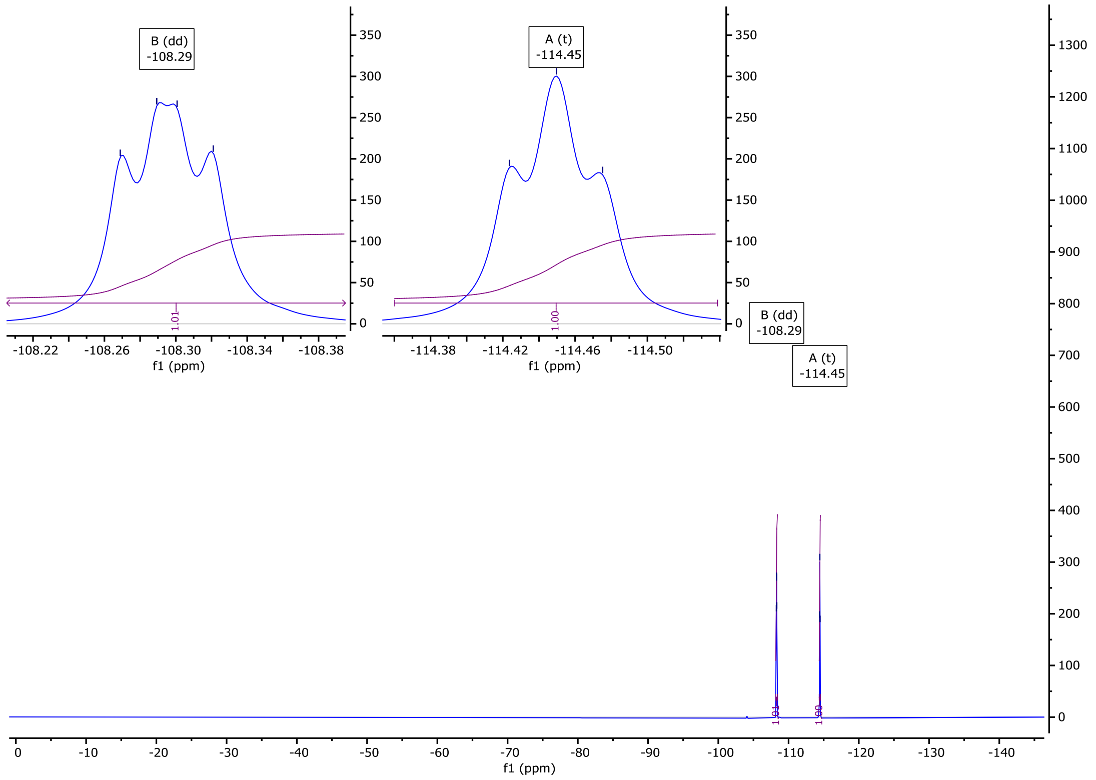

Figure S12: Chemical structure, NMR spectra (^{1}H (top), $^{13}C[1H]$ (middle), and ^{19}F (bottom)) spectra for **23 (0.1.1)**.

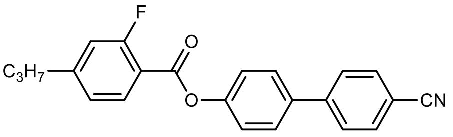
F
O
C_3H_7
O
CN

26 (0.0.1)

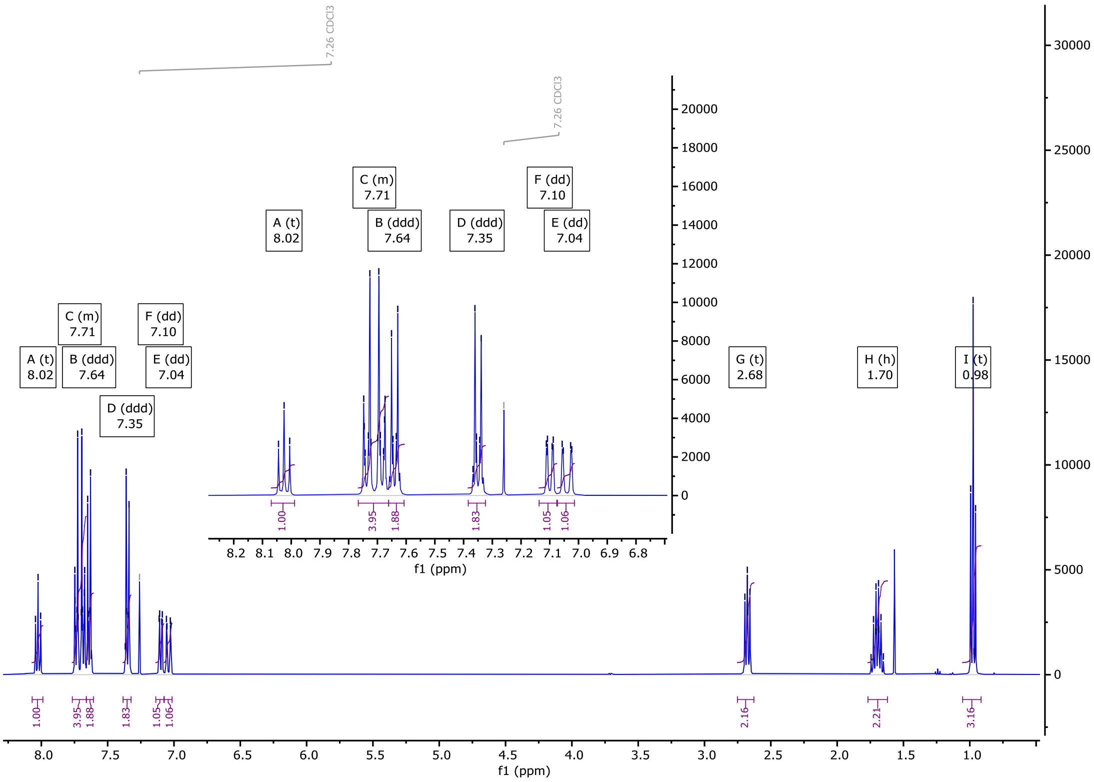
7.26 CDCl3
A (t) 8.02
B (ddd) 7.64
C (m) 7.71
D (ddd) 7.35
E (dd) 7.04
F (dd) 7.10
G (t) 2.68
H (h) 1.70
I (t) 0.98
f1 (ppm)

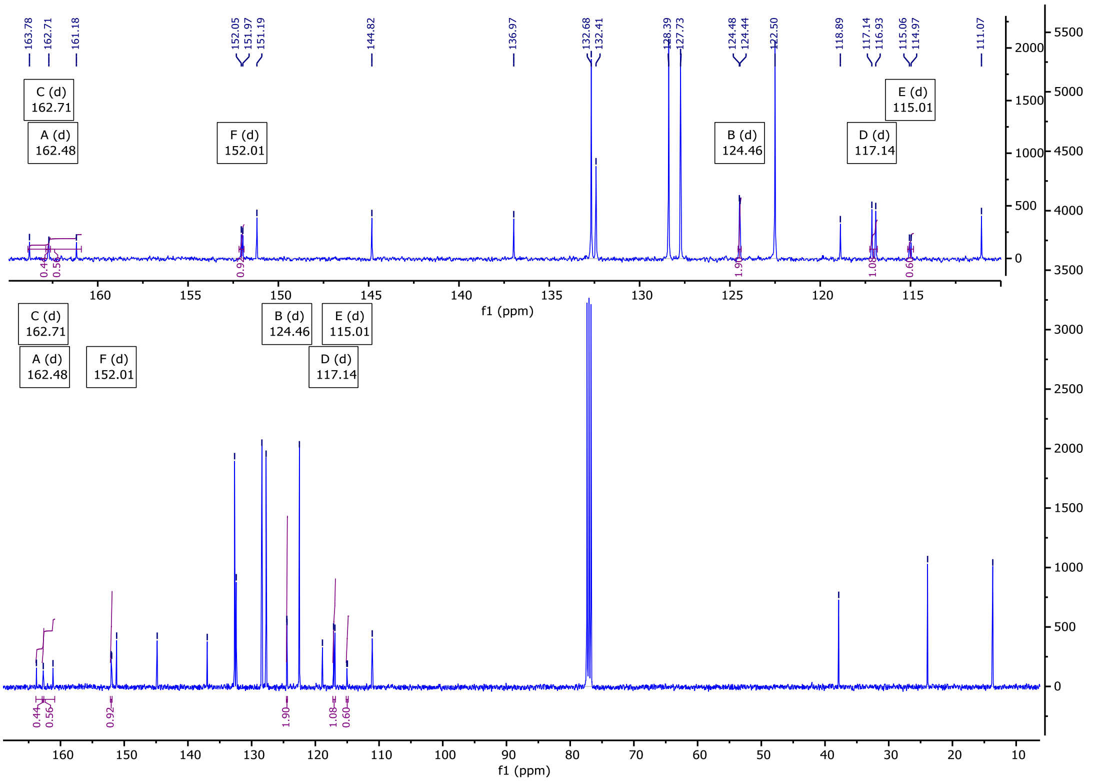
C (d) 162.71
A (d) 162.48
F (d) 152.01
B (d) 124.46
E (d) 115.01
D (d) 117.14
f1 (ppm)

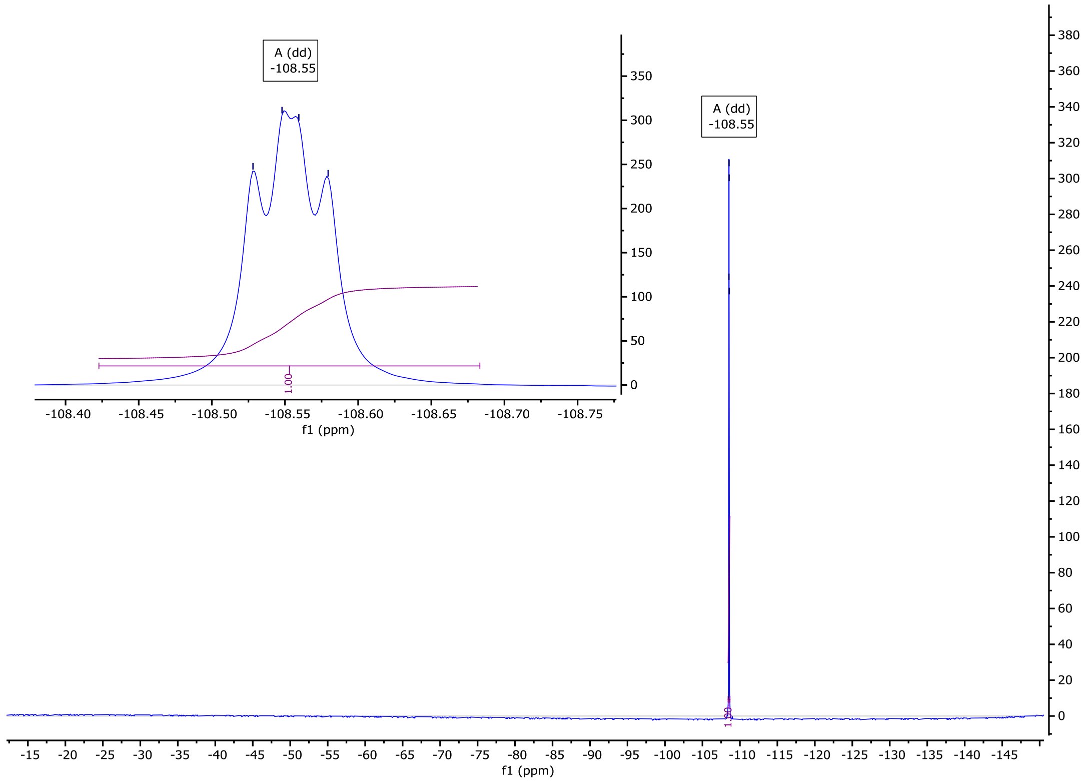

Figure S13: Chemical structure, NMR spectra (^{1}H (top), $^{13}C[1H]$ (middle), and ^{19}F (bottom)) spectra for **26 (0.0.1)**.

4 Supplemental references

[1] Martinot-Lagarde Ph., Direct electrical measurement of the permanent polarization of a ferroelectric chiral smectic C liquid crystal, J. Physique Lett. 38, 17 (1977).

[2] K. Miyasato, S. Abe, H. Takezoe, A. Fukuda, and E. Kuze, Direct Method with Triangular Waves for Measuring Spontaneous Polarization in Ferroelectric Liquid Crystals, Jpn J Appl Phys 22, L661 (1983).

[3] X. Chen et al., The smectic ZA phase: Antiferroelectric smectic order as a prelude to the ferroelectric nematic, PNAS 120, (2023).

[4] M. J. Frisch, G. W. Trucks, H. B. Schlegel, G .E. Scuseria, M. A. Robb, J. R. Cheeseman, G. Scalmani, V. Barone, B. Mennucci, G. A. Petersson, H. Nakatsuji, M. Caricato, X. Li, H. P. Hratchian, A. F. Izmaylov, J. Bloino, G. Zheng, J. L. Sonnenberg, M. Hada, M. Ehara, K. Toyota, R. Fukuda, J. Hasegawa, M. Ishida, T. Nakajima, Y. Honda, O. Kitao, H. Nakai, T. Vreven, J. A. Montgomery Jr., J. E. Peralta, F. Ogliaro, M. J. Bearpark, J. Heyd, E. N. Brothers, K. N. Kudin, V. N. Staroverov, R. Kobayashi, J. Normand, K. Raghavachari, A. P. Rendell, J. C. Burant, S. S. Iyengar, J. Tomasi, M. Cossi, N. Rega, N. J. Millam, M. Klene, J. E. Knox, J. B. Cross, V. Bakken, C. Adamo, J. Jaramillo, R. Gomperts, R. E. Stratmann, O. Yazyev, A. J. Austin,R. Cammi, C. Pomelli, J. W. Ochterski, R. L. Martin, K. Morokuma, V. G. Zakrzewski, G. A. Voth, P. Salvador, J. J. Dannenberg, S. Dapprich, A. D. Daniels, Ö. Farkas, J. B. Foresman ,J. V. Ortiz, J. Cioslowskiand, D. J. Fox, Gaussian 016, Revision E.01, Gaussian, Inc., Wallingford CT, 2016

[5] A. D. Becke, Density-functional thermochemistry. III. The role of exact exchange, Journal of Chemical Physics 98, 5648 (1993).

[6] C. Lee, W. Yang, and R. G. Parr, Development of the Colle-Salvetti correlation-energy formula into a functional of the electron density, Phys Rev B 37, 785 (1988).

[7] B. Hess, H. Bekker, H. J. C. Berendsen, and J. G. E. M. Fraaije, LINCS: A Linear Constraint Solver for molecular simulations, J Comput Chem 18, 1463 (1997).

[8] T. Lu and Q. Chen, Interaction Region Indicator: A Simple Real Space Function Clearly Revealing Both Chemical Bonds and Weak Interactions, Chemistry–Methods 1, 231 (2021).

[9] J. Hobbs, C. J. Gibb, and R. J. Mandle, Emergent Antiferroelectric Ordering and the Coupling of Liquid Crystalline and Polar Order, Small Science 2400189 (2024).

[10] Y. Sang, S. Han, S. Han, C. Pannecouque, E. De Clercq, C. Zhuang, and F. Chen, Follow on-based optimization of the biphenyl-DAPYs as HIV-1 nonnucleoside reverse

transcriptase inhibitors against the wild-type and mutant strains, Bioorg Chem 89, 102974 (2019).

[11] G. W. Gray, M. Hird, D. Lacey, and K. J. Toyne, The Synthesis and Transition Temperatures of Some Fluoro-Substituted 4-Cyanophenyl and 4-Cyanobiphenyl-4'-yl 4-Pentyl- and 4-Butoxy-Benzoates, Molecular Crystals and Liquid Crystals Incorporating Nonlinear Optics 172, 165 (1989).

[12] P. Kirsch, E. Montenegro, L. D. Farrand, D. Pauluth, M. Heckmeier, Liquid crystal materials characterised by the chemical structure of the liquid crystal components, e.g. by a specific unit the structure containing one or more specific, optionally substituted ring or ring systems. WO2005019378A1, 2010.

[13] H. Mingjun, X. Xiaochen, S. Yahohao, L. Jinxing, W. Zhidong, X. Runli, Optically active compound, liquid crystal composition containing the same and liquid crystal device using the composition. JP2002226451A, 2002.
